# Human tau mutations in cerebral organoids induce a progressive dyshomeostasis of cholesterol

**DOI:** 10.1016/j.stemcr.2022.07.011

**Published:** 2022-08-18

**Authors:** Stella M.K. Glasauer, Susan K. Goderie, Jennifer N. Rauch, Elmer Guzman, Morgane Audouard, Taylor Bertucci, Shona Joy, Emma Rommelfanger, Gabriel Luna, Erica Keane-Rivera, Steven Lotz, Susan Borden, Aaron M. Armando, Oswald Quehenberger, Sally Temple, Kenneth S. Kosik

**Affiliations:** 1 Neuroscience Research Institute and Department of Molecular, Cellular and Developmental Biology, University of California, Santa Barbara, Santa Barbara, CA 93106, USA; 2 Neural Stem Cell Institute, Rensselaer, NY 12144, USA; 3 Department of Pharmacology, University of California, San Diego, San Diego, CA 92093, USA

**Keywords:** tau, MAPT mutation, tauopathy, neurodegeneration, disease model, brain organoid, single-cell RNA sequencing, astrocyte, cholesterol

## Abstract

Mutations in the *MAPT* gene that encodes tau lead to frontotemporal dementia (FTD) with pathology evident in both cerebral neurons and glia. Human cerebral organoids (hCOs) from individuals harboring pathogenic tau mutations can reveal the earliest downstream effects on molecular pathways within a developmental context, generating interacting neurons and glia. We found that in hCOs carrying the V337M and R406W tau mutations, the cholesterol biosynthesis pathway in astrocytes was the top upregulated gene set compared with isogenic controls by single-cell RNA sequencing (scRNA-seq). The 15 upregulated genes included *HMGCR*, *ACAT2*, *STARD4*, *LDLR*, and *SREBF2*. This result was confirmed in a homozygous R406W mutant cell line by immunostaining and sterol measurements. Cholesterol abundance in the brain is tightly regulated by efflux and cholesterol biosynthetic enzyme levels in astrocytes, and dysregulation can cause aberrant phosphorylation of tau. Our findings suggest that cholesterol dyshomeostasis is an early event in the etiology of neurodegeneration caused by tau mutations.

## Highlights

• Cerebral organoid models of tauopathy caused by *MAPT* mutations
• Upregulated cholesterol and fatty acid biosynthesis genes in *MAPT* mutant astrocytes
• Elevation of cholesterol and its precursors in *MAPT* mutant cerebral organoids

## Graphical abstract

**Figure:**
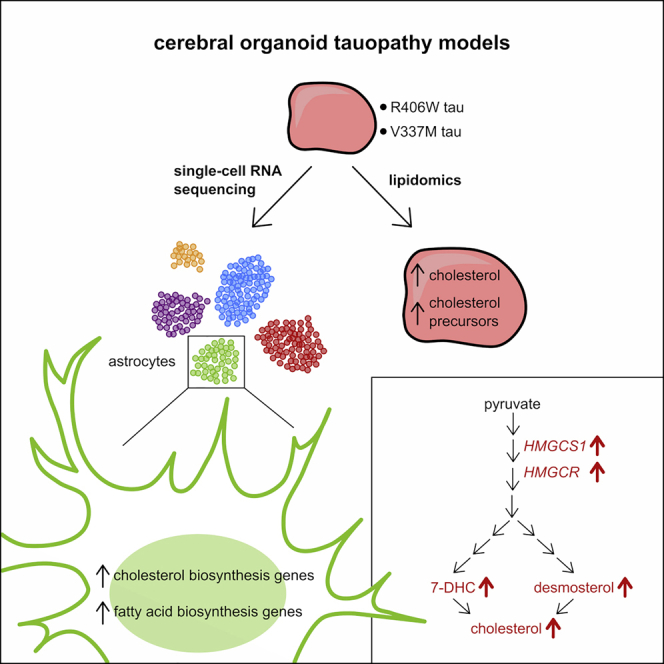

## Teaser

In this article, Kosik and colleagues present single-cell RNA sequencing data of cerebral organoids with *MAPT* mutations causing neurodegeneration. Genes encoding cholesterol biosynthesis enzymes were upregulated in astrocytes of *MAPT* mutant organoids. Cholesterol and its precursors become elevated as a consequence of *MAPT* mutations during organoid maturation, suggesting that cholesterol is dysregulated early in disease etiology.

## Introduction

Among individuals with frontotemporal dementia (FTD) due to tauopathies, those with tau mutations represent paradigmatic exemplars of this entire disease category because the mutations clearly position the tau gene as the underlying cause of the disease. With the implication of tau mutations as the clear initiator of the disease process, the current challenge is to track the downstream molecular pathways that ultimately lead to the complex pathological and clinical phenotypes collectively referred to as FTD. The preparation of human cerebral organoids (hCOs) from induced pluripotent stem cells (iPSCs) harvested from mutation carriers and their isogenic controls is a potentially informative approach to detect the effects of mutations on cell types over development and maturation. Numerous studies indicate the emergence of cell populations with at least some degree of maturation including synaptic structures and neural network activity (Sidhaye and Knoblich, 2021).

In support of this approach, hCOs with *APP* and *PSEN1* mutations, hCOs generated from Down syndrome iPSCs, and hCOs carrying the Alzheimer disease (AD) APOE4 risk allele show AD hallmarks, including amyloid-beta accumulation and tau hyperphosphorylation (Raja et al., 2016; Gonzalez et al., 2018; Lee et al., 2016). hCOs with the *MAPT* V337M mutation have been reported to have increased tau phosphorylation, glutamatergic dysfunction, and glutamatergic neuron loss (Bowles et al., 2021). Importantly, the effects of tau mutations appear to begin during development, an inference that molecular pathology is evident before tau inclusions appear (Hernandez et al., 2019; Jiang et al., 2018).

Nonneuronal cells are increasingly recognized as critical players in neurodegenerative disease, including tauopathies (De Strooper and Karran 2016). Astrocytes can affect disease onset and progression (Matias et al. 2019) but have been less characterized than neurons in neurodegeneration. In FTD, astrogliosis and astrocytic degeneration are observed even at early disease stages (Broe et al., 2004), suggesting that molecular changes in astrocytes are early events in the etiology of FTD.

Using a guided approach for hCO production (Yoon et al., 2019), we generated a diversity of cell types with a focus on the astrocyte population. We performed single-cell RNA sequencing (scRNA-seq) on a set of hCOs from human iPSC lines with tau mutations and their isogenic controls to search for dysregulation of molecular pathways. The cholesterol synthetic pathway emerged as dysregulated in astrocytes from hCOs with tau mutations. Astrocytes are the predominant cell type that produce cholesterol in the adult brain (Saher and Stumpf, 2015). We validated the findings with immunohistochemistry and lipidomics. Accompanying the astrocytic changes, we observed decreased expression of glycolytic and GABA receptor genes in pyramidal neurons, showing that both neurons and astrocytes are affected by *MAPT* mutations. As cholesterol metabolism regulates aberrant tau phosphorylation (van der Kant et al., 2019), our study reveals astrocytic cholesterol biosynthesis as a disease-relevant pathway affected early on in the etiology of neurodegeneration.

## Results

### Cellular diversity of hCOs derived from *MAPT* mutant carriers and isogenic controls

**Figure 1:**
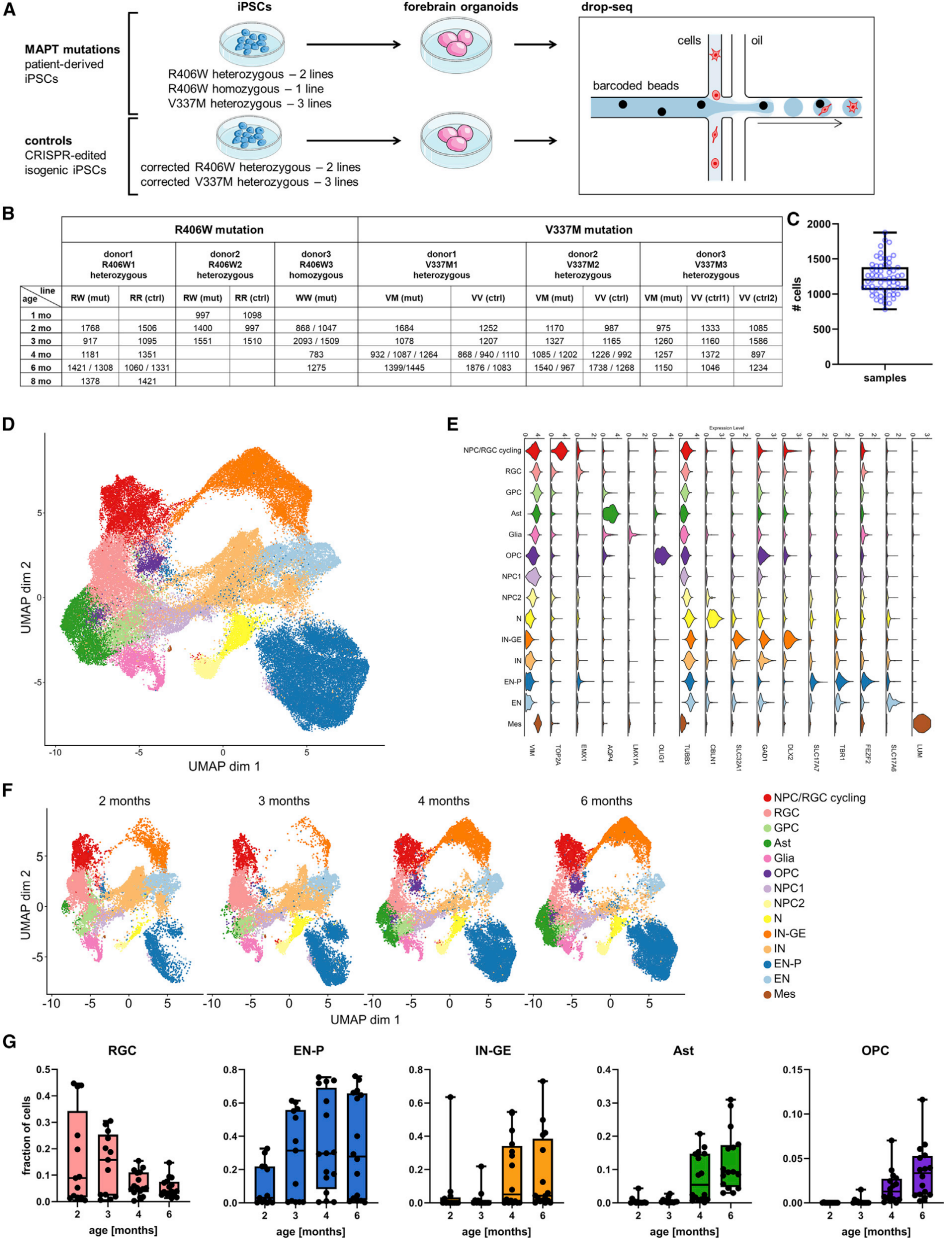
Composition of the human cerebral organoid (hCO) single-cell RNA dataset (A) iPSCs from 6 donors with *MAPT* mutations and 5 isogenic controls were grown into hCOs and subjected to drop-seq. (B) Overview of single-cell (sc) dataset and numbers of cells sequenced. Entries with more than one number indicate replicates from independent differentiation experiments, each number representing the number of cells sequenced for a replicate. For full line names, see supplemental experimental procedures. (C) Cell numbers sequenced per sample (1,228 cells, mean ± 239.6 SD). Box and whiskers plots represent median (line in box center), first and third quartile (lower and upper box border, respectively), and minimum and maximum values (whiskers). (D) UMAP of entire dataset, 62 samples and 76,111 cells. Colors represent cell types. (E) Expression of selected canonical markers. (F) UMAP subsetted to ages in months. (G) Relative abundances (n cells in a cluster divided by total n of cells in a sample) of cell populations over hCO development. Box and whiskers plots represent median (line in box center), first and third quartile (lower and upper box border, respectively), and minimum and maximum values (whiskers). Ast, astrocytes; Glia, glia expressing markers of choroid plexus, ependymal cells and Wnt/Bmp signaling molecules; EN, excitatory neurons; EN-P, pyramidal neurons; GPC, glial progenitor cells; IN, inhibitory neurons; IN-GE, inhibitory neurons derived from ganglionic eminences; Mes, mesenchymal cells; N, neurons; NPC1, neuronal progenitors 1; NPC2, neuronal progenitors 2; NPC/RGC-cycling, cycling neural progenitors and radial glia; RGC, radial glia; OPC, oligodendrocyte progenitors. See also Figure S1.

Tau pathology in FTD and AD massively affects the cerebral cortex (Arendt et al., 2016). To model the effect of pathogenic *MAPT* mutations in the human cerebral cortex, we relied on hCOs generated by directed differentiation (Yoon et al., 2019). iPSCs from two heterozygous *MAPT* R406W carriers, one homozygous R406W carrier and three heterozygous V337M, and CRISPR-corrected isogenic control for each heterozygous line were used for hCO production (Figures 1A and 1B; see experimental procedures for full line names and abbreviations). We used drop-seq (Macosko et al., 2015) to obtain sc transcriptomes for a total of 62 hCO samples, each consisting of three to four pooled hCOs, and 76,111 total cells post-filtering, with a mean of 1,228 cells ± 239.6 SD per sample (Figures 1B and 1C).

Clustering and uniform manifold approximation and projection (UMAP) of the dataset resulted in 14 major cell populations (Figure 1D; see experimental procedures). Marker genes for each cell population were computed (Table S1), manually probed for cell-type-specific genes found in the literature and in the PanglaoDB database (Franzén et al., 2019) (Figures 1E and S1A), and compared with published sc datasets (Giandomenico et al., 2019; Polioudakis et al., 2019; Quadrato et al., 2017; Velmeshev et al., 2019). The manually annotated identities were further confirmed by Gene Ontology (GO) enrichment analysis of the marker gene lists (Table S2).

The following neuronal populations were identified (Figures 1D and 1E): excitatory neurons expressing pyramidal cell (EN-P) markers (expressing, e.g., *BCL11B*, *SOX5*, *TBR1*, *SATB2*, *FEZF2*, *SLCC17A7*); GABAergic neurons expressing markers of the ventral forebrain and, more specifically, the lateral ganglionic eminences and dorsal portion of the caudal ganglionic eminences according to Long et al. (2009) (IN-GEs; expressing, e.g., *GAD1*, *GAD2*, *DLX1*,*2*,*5*,*6, ETV1*, *PBX3*, *ARX*, *SP9*); largely unidentified excitatory neurons (ENs; expressing, e.g., *MAPT*, *SLC17A6*, *SCN2A*, *ANKD3*); largely unidentified GABAergic neurons (INs; expressing, e.g., *GAD1*, *GAD2*); and other unidentified neurons (Ns; expressing, e.g., synaptic genes *SYT1*, *CPLX2*, *SYNJ2*). Two populations were identified as presumed neuronal progenitors based on low levels of both glial and neuronally expressed genes (neural progenitor cell [NPC1] and NPC2). Furthermore, we identified glial populations: radial glia (radial glia cells [RGCs]; expressing, e.g., *VIM*, *SOX2*, *PAX6*, *EMX2*); actively proliferating cells (RGC/NPC cycling; expressing, e.g., *VIM*, *EMX2*, *DLX1*, *TOP2A*, *CENPF*); largely unidentified glia expressing markers of choroid plexus, ependymal cells, and signaling molecules secreted by cortical hem and rhombic lip (glia; e.g., *TTR*, *HTR2C*, *TM4SF1*, *EFNB3*, *WNT2B*, *BMP7*); oligodendrocyte progenitors (OPCs; expressing, e.g., *OLIG1*, *OLIG2*, *PDGFRA*); astrocytes (Asts; expressing, e.g., *SLC1A2*, *GFAP*, *S100B*, *AQP4*); and a presumptive glial progenitor population (glial progenitor cells [GPCs]) expressing astrocyte markers (*SLC1A3*, *GFAP*, *S100B*) at levels intermediate between RGCs and Asts. A very small cluster of putative mesenchymal cells (Mess), similar to a population described in Camp et al. (2015), was identified (expressing, e.g., *LUM*, *DCN*, and multiple genes encoding collagens). Although the proportion of inhibitory neurons was larger than that reported in Yoon et al. (2019), the EN-P population was the predominant cell type in our dataset (Figure 1D).

The abundances of EN-Ps, IN-GEs, OPCs, and Asts increased over time whereas NPCs/RGCs decreased (Figures 1F, 1G, and S1C), as expected during hCO maturation (Lancaster et al., 2013, Paşca et al., 2015, Quadrato et al., 2017, Renner et al., 2017, Sloan et al., 2017) . However, a small minority of hCO preparations did not show abundant EN-Ps expected in hCOs (Figure S1B; supplemental experimental procedures) but either a high abundance of IN-GEs or unidentified neurons (Ns, INs, ENs) (Figure S1B). Such preparations were excluded from the analysis of astrocytes as described in the results. We did not detect significant effects of the *MAPT* mutations on the relative abundances of the 14 major cell populations (Figure S2D). However, given the variation in cellular composition (Figures S1B–S1D), it seems unlikely that we would be able to detect potentially subtle effects of *MAPT* mutations on cell type abundances.

### Downregulation of the glycolytic pathway and GABA receptor genes in pyramidal neurons

**Figure 2:**
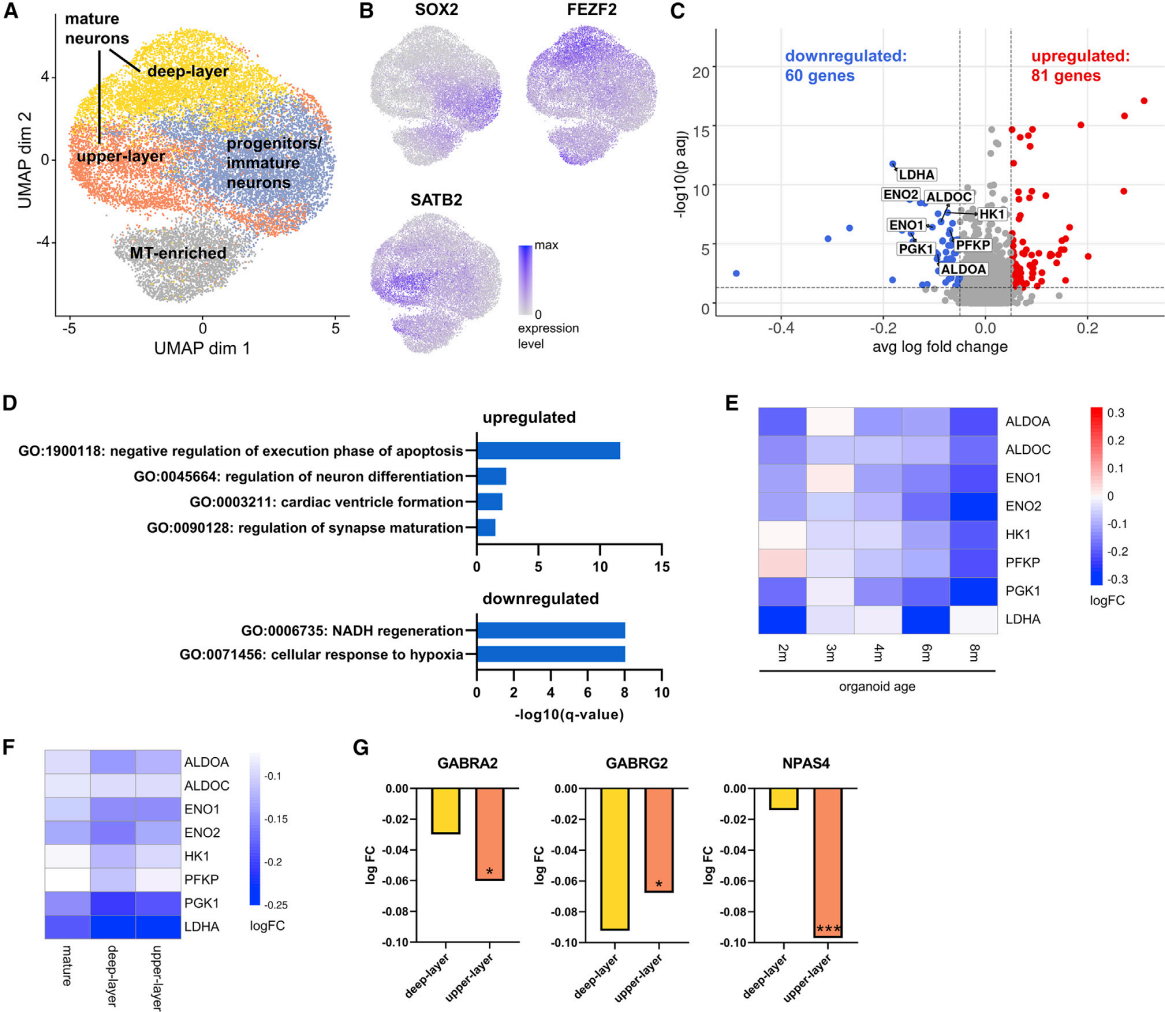
Effects of *MAPT* mutations on pyramidal glutamatergic neurons (A) UMAP and subclustering of EN-Ps. (B) Expression of selected subcluster markers. (C) Volcano plot of differential gene expression (DGE) results of *MAPT* mutant versus control mature neurons, combining isogenic pairs with ≥30 mature neurons in each sample (n = 10 isogenic pairs from 5 hCO batches, 4,044 cells). Differentially expressed genes (DEGs) were identified using the MAST test (Finak et al., 2015) with a logFC cutoff of 0.05 and Benjamini-Hochberg (BH)-corrected p values <0.05. DEGs involved in glycolysis are highlighted. (D) Gene Ontology (GO) enrichment of genes up- (top) and downregulated (bottom) in *MAPT* mutant mature EN-Ps. (E) Heatmap of logFC of glycolysis-related genes identified in (C) across different ages. (F) Heatmap of logFC of glycolysis-related genes identified in (C), and logFC in deep- and upper-layer neurons. (G) Downregulation of GABA receptor genes *GABRA2* and *GABRG2*, and the immediate-early gene *NPAS4* in upper-layer neurons. Negative logFC indicates decreased gene expression in heterozygous mutant compared with control, positive logFC the opposite. ^∗^p ≤ 0.05, ^∗∗∗^p ≤ 0.001. See also Figures S2 and S3.

Having identified the major cell types, we focused on effects of *MAPT* mutations on the population corresponding to pyramidal neurons (EN-Ps), as they are heavily impaired in neurodegenerative diseases (Fu et al., 2018). The EN-P cluster consists of distinctive subgroups (Figures 2A, 2B, S2A, and S2C): an intermediate progenitor and immature neuron cluster, two clusters enriched in deep- and upper-layer neurons, respectively, and a cluster characterized by high expression of mitochondrially encoded genes indicating cell damage or high metabolic demand (Figures 2A, 2B, and S2C). No statistically significant enrichment of mutant cells in any subcluster was found (Figures S2B and S2D).

Differential gene expression (DGE) in the clusters containing upper- and deep-layer-enriched clusters combined (“mature neurons”; Figure 2A) in ten mutant-isogenic pairs (from 5 hCO batches) with 4,044 cells yielded 60 downregulated genes (Figure 2C; Table S3), associated with the two top enriched GO terms (“NADH regeneration” and “cellular response to hypoxia”) (Figure 2D), both containing multiple enzymes in glycolysis (*ALDOA*, *ALDOC*, *ENO1*, *ENO2*, *HK1*, *PFKP*, *PGK1*) and *LDHA*, converting pyruvate into lactate (Figures 2C and 2E). Expression levels of these genes were decreased in the *MAPT* mutant across hCO ages (Figure 2E) and across isogenic pairs (Figure S3A). Decreased glucose metabolism has been reported from AD brains preceding memory deficits and during normal aging, while an increase in glycolysis can be neuroprotective (Goyal et al., 2017, Nordberg et al., 2010, Tang, 2020). The top GO term of the 81 upregulated genes (“negative regulation of execution phase of apoptosis”) (Figures 2C and 2D) predominantly included homologs of the mitochondrial *MT-RNR2* gene (*MTRNR2L* genes). Although their functions are poorly understood, upregulation of *MTRNR2L* genes has also been reported in a sc sequencing study of AD brains (Mathys et al., 2019). Glycolysis-related genes were also downregulated in mutant cells when analyzing upper- and deep-layer clusters separately (Figures S3B and S3C; Table S3), and the glycolytic genes identified in the analysis of mature neurons (Figures 2C and 2D) showed negative changes in both deep- and upper-layer clusters (Figure 2F).

Downregulated genes in upper-layer neurons were enriched in “inhibitory synapse assembly,” including *GABRA2*, *GABRG2*, and *NPAS4*, (Figures 2G and S3C), consistent with downregulation of GABA receptor genes in *MAPT* R406W iPSC-derived neurons and brains of *MAPT* R406W carriers (Jiang et al., 2018). A subset of GABA receptor and glycolytic genes was also downregulated in the homozygous R406W line compared with controls (Figure S3D; Table S3). These results show that *MAPT* mutations induce transcriptional changes in the glycolysis pathway and GABA receptors, both of which have been shown to be impacted in neurodegeneration. We next investigated how astrocytes participate in this disease environment set by *MAPT* mutations.

### The cholesterol biosynthesis pathway is upregulated in *MAPT* mutant astrocytes

**Figure 3:**
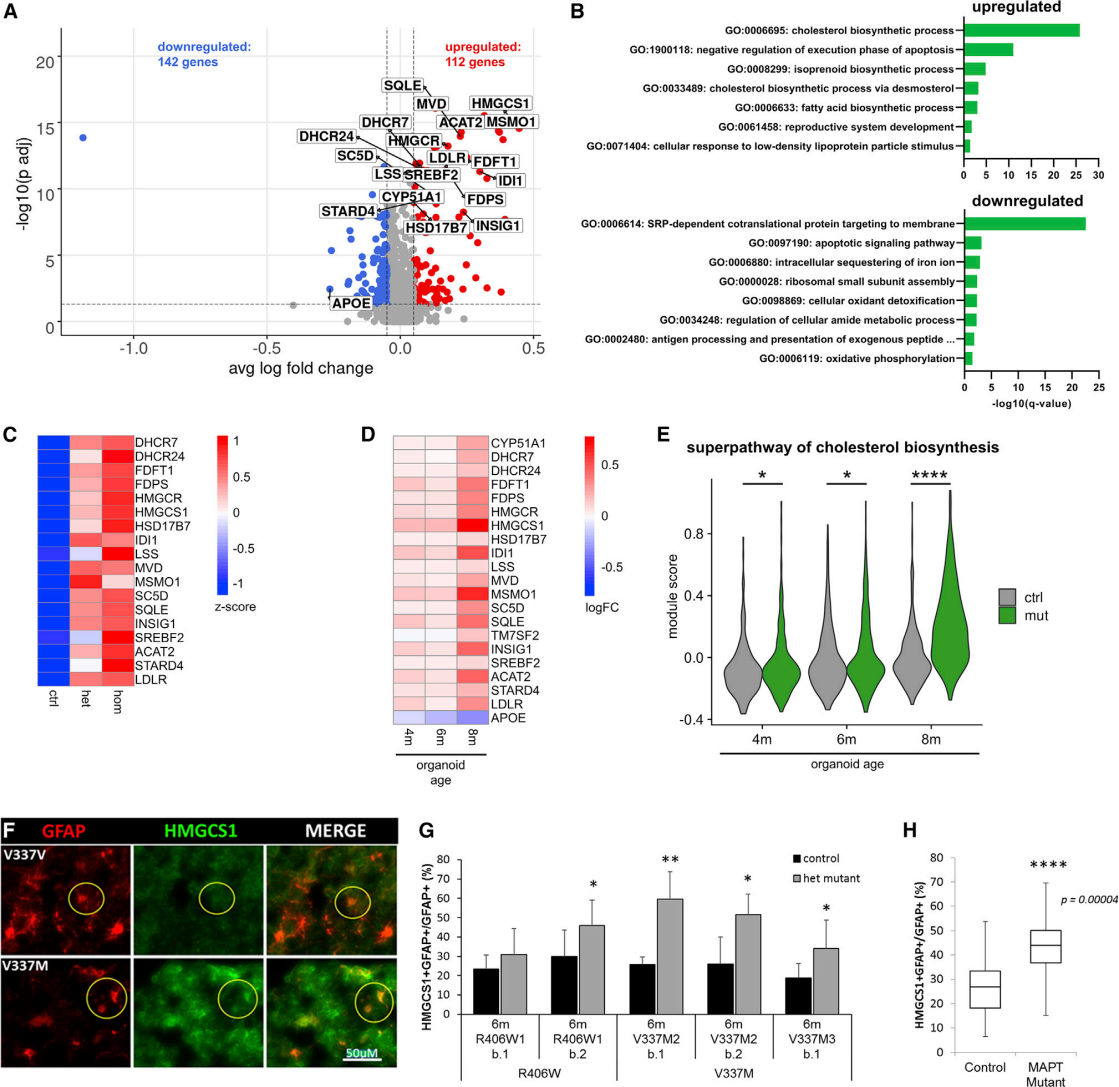
Effects of *MAPT* mutations on astrocytes (A) Volcano plot of DGE in astrocytes of isogenic pairs with >5% EN-Ps and <50% INs, ENs, and Ns in each sample (n = 6 isogenic pairs from 4 hCO batches, 1,802 cells) using the MAST test with a logFC cutoff of 0.05 and BH-corrected p values <0.05. Cholesterol-related DEGs are highlighted. (B) GO enrichment of genes up- (top) and downregulated (bottom) in *MAPT* mutant astrocytes including isogenic pairs with at least 30 mature astrocytes in each sample (n = 11 isogenic pairs from 7 hCO batches, 3,398 cells). (C) Expression levels (*Z* scores) of cholesterol-related genes in astrocytes from control, heterozygous R406W and V337M mutant, homozygous R406W mutant. (D) Heatmap of logFC of cholesterol-related genes identified in (A) across hCO ages. Positive logFC indicates increased gene expression in mutant (heterozygous and homozygous combined) compared with control, negative logFC the opposite. (E) Violin plots showing gene signature enrichment scores of a cholesterol biosynthetic gene set (“superpathway of cholesterol biosynthesis” deposited in the Human Cyc database, 25 genes) across ages (4 months: n = 247 control cells, 254 mutant cells; 6 months, n = 286 control cells, 424 mutant cells; 8 months, n = 375 control cells, 375 mutant cells) and genotypes (mut, homozygous and heterozygous combined; ctrl, control cells). Statistical significance was determined using two-sided Wilcoxon rank-sum test and BH-corrected p values for 3 comparisons. (F) Representative image of HMGCS1 and GFAP co-labeling in 6-month-old V337M mutant hCOs and isogenic control. (G) Percentages of HMGCS1-positive astrocytes (GFAP+) from two R406W and three V337M isogenic pairs across three hCO prep dates at 6 months. Bars and error bars represent means ± SD. (H) Percentages of HMGCS1+ astrocytes (GFAP+) from all three isogenic *MAPT* mutant and control lines. Box and whiskers plots represent median (line in box center), first and third quartile (lower and upper box border, respectively), and minimum and maximum values (whiskers). Student’s t test was used to determine significance. ^∗^p ≤ 0.1, ^∗∗^p ≤ 0.01, ^∗∗∗∗^p ≤ 0.0001. See also Figure S4.

A subset of the hCO preparations showed unexpected cellular composition. We did not include these samples, but restricted our analysis to isogenic pairs that had >5% EN-Ps and <50% unidentified neurons (INs, ENs, Ns) in each sample, since the neuronal environment and regional identity affect astrocytes’ transcriptional profiles (Morel et al., 2017; Clarke et al., 2021). Five of the 22 samples containing sufficient astrocytes for analysis (supplemental experimental procedures) did not meet these criteria, resulting in the removal of five isogenic pairs from astrocyte analyses (Figure S1B). No statistically significant enrichment of *MAPT* mutant cells was found among astrocyte subclusters (Figures S4A–S4C), suggesting no population expansion or constriction effects of *MAPT* mutations. We next performed DGE analysis of astrocytes from hCO ages 4, 6, and 8 months combined, identifying 112 up- and 142 downregulated genes (Figure 3A; Table S3). “Cholesterol biosynthesis” was the top enriched GO term for upregulated genes in *MAPT* mutant astrocytes (Figure 3B). Fifteen of these genes encode enzymes of the cholesterol synthesis pathway, including its rate-limiting enzyme HMGCR (Figure 3A). Also, *ACAT2*, encoding an enzyme converting cholesterol to its storage form cholesteryl esters, *STARD4*, encoding an intracellular cholesterol transporter, *LDLR*, encoding an important receptor for cholesterol uptake, and *SREBF2*, encoding a transcriptional activator of cholesterol biosynthesis enzymes, were significantly upregulated (Figure 3A).

The increased expression of cholesterol biosynthesis genes was further confirmed in hCOs from the R406W homozygous line compared with control samples (Figure S4D; Table S3). GO enrichment analysis of the upregulated genes again revealed the cholesterol biosynthesis pathway as a top category (Figure S4E). Eighteen out of the 21 cholesterol-related genes identified as differentially expressed (DE) in the heterozygous mutants were also DE in the homozygous R406W mutant (Figure 3C).

We next identified age effects across 4–8 months (i.e., stages when astrocytes are present), revealing positive log fold changes (FC) of the previously identified DE cholesterol-related genes (Figure 3A) across ages (heterozygous and homozygous combined; Figure 3D). Furthermore, examination of a cholesterol biosynthesis signature (“superpathway of cholesterol biosynthesis,” consisting of 25 genes; Caspi et al., 2018) in genotypes and across ages revealed significant increases in the expression of this signature in mutant (heterozygous and homozygous combined) versus control astrocytes at 4, 6, and 8 months of age, with the largest size effect and significance level at 8 months (Figure 3E).

The transcription factor SREBF2 activates cholesterol biosynthesis genes and genes acting in long-chain fatty acid synthesis (Madison 2016). GO terms related to fatty acid synthesis were enriched in the genes upregulated in *MAPT* mutant astrocytes (Figure 3B), including the fatty acid synthase gene *FASN* (Figure S4F), modulating neurodegeneration-related toxicities (Ates et al., 2020). All eight upregulated genes included in the GO term “fatty acid biosynthetic process” were also upregulated in the homozygous R406W mutant line (Figure S5F).

Concomitantly, we identified downregulation of *APOE* in *MAPT* mutant astrocytes (Figure 3A). *APOE* encodes an apolipoprotein important for brain cholesterol transport and is a prominent risk gene for AD (Corder et al., 1993). Genes involved in cholesterol synthesis and the *APOE* gene show the same directionalities of change in lines with APOE3/3 (2 lines), APOE2/3 (1 line), and APOE4/4 (1 line) genotypes (Figure S4G; supplemental experimental procedures), indicating that these changes may occur irrespective of the APOE variant.

To rule out the possibility that our filtering had artificially produced the finding of upregulated cholesterol biosynthetic enzymes, we performed DGE testing on all isogenic pairs (Figure S4H; Table S3). This approach still resulted in the cholesterol biosynthesis pathway as upregulated according to GO enrichment terms (Figure S4I) and upregulated *HMGCR* (Figure S4H). However, the directionality of change of the previously identified cholesterol-related DE genes (Figure 3A) was more consistently positive in the isogenic pairs that did meet the criteria stated above (Figure S4G). No population expansion or restriction effects were present in either approach (Figures S4C and S4J). *MAPT* was expressed in mutant and control astrocytes, albeit at lower levels than in neurons, (Figure S4K), opening up the possibility of cell-autonomous effects of *MAPT* mutations on astrocytes.

We validated upregulation of a cholesterol biosynthetic enzyme with immunohistochemistry: co-labeling of HMGCS1, encoded by the cholesterol-related gene with the highest logFC in our analysis, and the astrocyte marker GFAP revealed a higher proportion of HMGCS1-positive astrocytes in mutant hCOs compared with control.

### Age-dependent elevation of cholesterol and its precursors in *MAPT* mutant organoids

**Figure 4:**
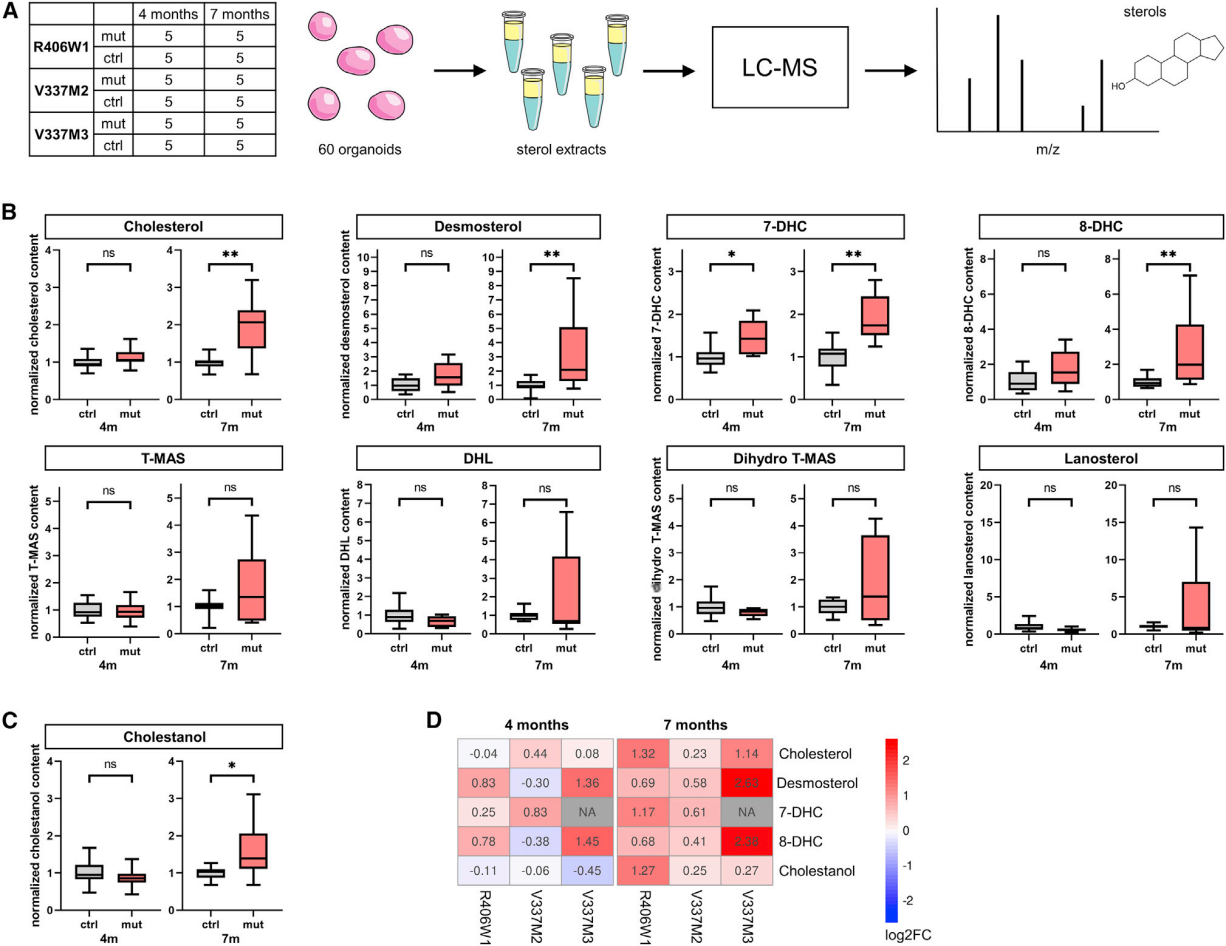
Sterol quantification in *MAPT* mutant hCOs (A) Mutant and control hCOs from three isogenic lines were subjected to sterol analysis with liquid chromatography-mass spectrometry (LC-MS). Five individual hCOs from each line were analyzed at 4 and 7 months hCO age. (B and C) LC-MS data of all three isogenic lines combined. Metabolites detected in >50% hCO samples at each time point were analyzed. n = 15 from 3 hCO batches for each control and mutant at each time point except for 7-DHC (n = 10 from 2 hCO batches for each group at each time point), T-MAS (n = 10 from 2 hCO batches for each group at each time point), and Dihydro T-MAS (4 months control n = 14 from 3 hCO batches, 4 months mutant n = 15 from 3 hCO batches, 7 months n = 10 from 2 hCO batches for each group); see also Figure S5C. Mutant values were normalized to control. Box and whiskers plots represent median (line in box center), first and third quartile (lower and upper box border, respectively), and minimum and maximum values (whiskers). Significance was determined using Wilcoxon rank-sum test. BH-corrected p values (for 22 multiple comparisons) <0.05 are reported as statistically significant. ^∗^p ≤ 0.05, ^∗∗^p ≤ 0.01. (D) Heatmap of log2FC for the three isogenic pairs. Positive log2FC indicates increased concentrations in mutant hCOs compared with control, negative log2FC the opposite. See also Figure S5.

To investigate whether the observed changes in the cholesterol biosynthesis pathway were accompanied by altered levels of cholesterol and its biosynthesis intermediates, we utilized liquid chromatography-mass spectrometry (LC-MS)-based lipidomics. hCOs from one R406W heterozygous line (R406W1) and two V337M heterozygous lines (V337M2, V337M3) and their respective isogenic control were analyzed for sterols at ages 4 and 7 months. We analyzed five hCOs per age group and cell line and therefore a total of 60 individual hCOs (Figure 4A). The sterol panel included cholesterol and 11 of its precursors (Figure S5A). For statistical analysis, we included metabolites that were detected in at least 50% of the hCO samples in each of the age groups (Figures 4B, 4C, S5B, and S5C), resulting in a set of cholesterol and 7 of its precursors (Figure 4B), the cholesterol derivative cholestanol (Figure 4C), and two phytosterols (Figure S5A). Other cholesterol biosynthesis intermediates were not detected in most samples, likely due to small size of hCOs and therefore low sample input.

A combined analysis of all hCOs within each time point revealed a significant increase of cholesterol, its precursors desmosterol, 7-dehydrocholesterol (7-DHC), 8-DHC, and the cholesterol derivative cholestanol at 7 months (Figures 4B and 4C). Cholestanol accumulates in cerebrotendinous xanthomatosis, often involving neurological symptoms (Björkhem 2013). Each isogenic pair (R406W1, V337M2, V337M3) consistently showed increased cholesterol, desmosterol, 7-DHC, 8-DHC, and cholestanol at 7 months (Figures 4D and S5B). At 4 months, only 7-DHC was significantly increased (Figure 4B). Levels of precursors generated in earlier steps of cholesterol synthesis (T-MAS, DHL, Dihydro T-MAS, lanosterol) were not altered at either time point. This pattern may be explained by elevated cholesterol levels resulting in product inhibition (Cornish-Bowden, 2013) of the enzymes catalyzing the last steps of cholesterol biosynthesis.

**Figure 5:**
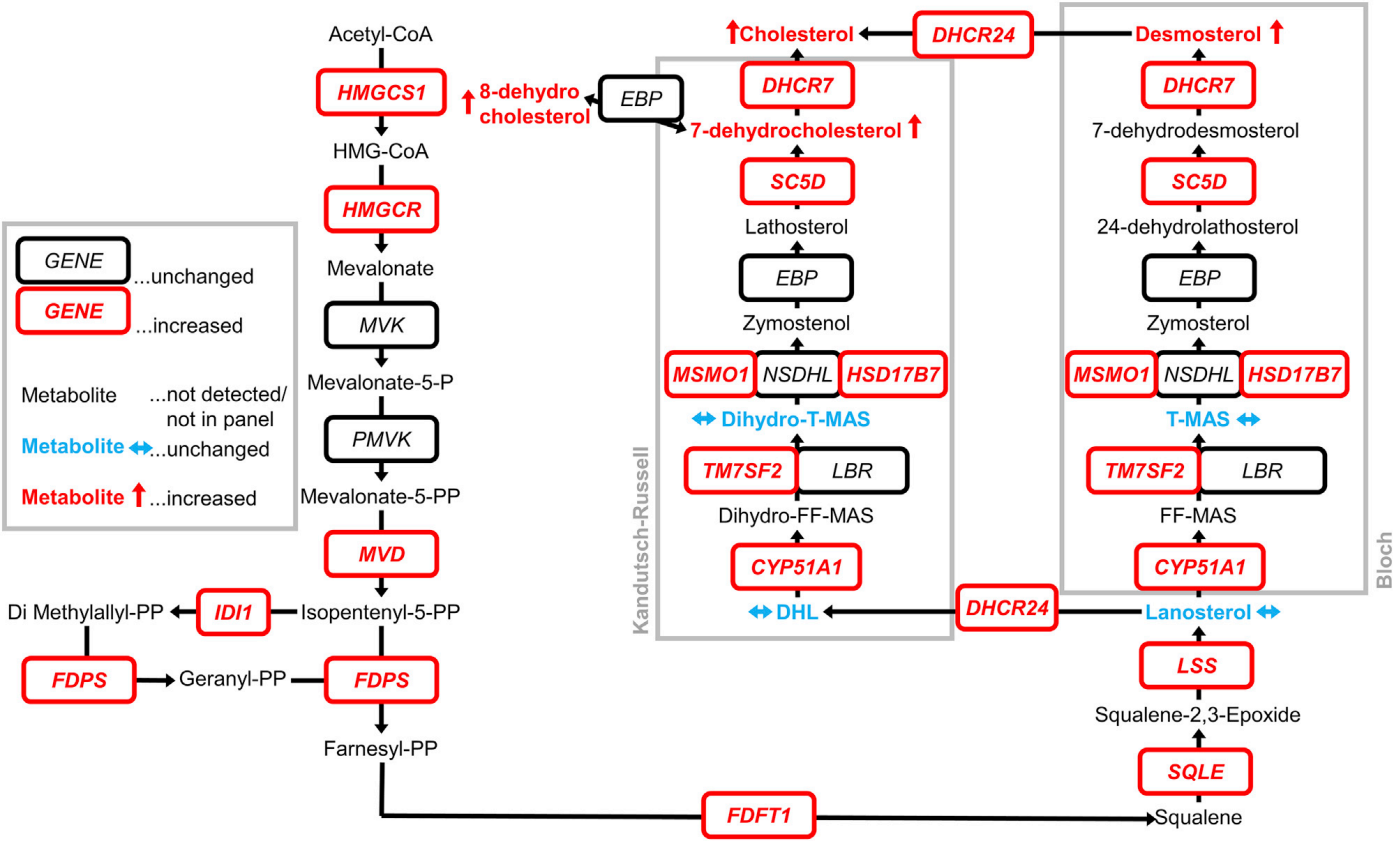
Summary of changes in cholesterol biosynthesis caused by *MAPT* mutations Boxed/italic, genes encoding cholesterol biosynthetic enzymes; red/boxed/italic, genes with increased expression in *MAPT* mutant astrocytes, determined by scRNA-seq; black/boxed/italic, genes with unaltered expression; unboxed, metabolites (cholesterol and its precursors); red/arrow up, metabolites with increased levels in *MAPT* mutant hCOs determined by LC-MS; blue/arrow sideways, metabolites with unaltered levels; black, metabolites not detected or not included in the sterol panel.

Summarized, lipidomics corroborate our transcriptomic and immunohistochemical findings of dysregulated cholesterol synthesis (Figure 5), and the increase of metabolites lags behind the increase in the relevant mRNAs.

## Discussion

The difficulty in distinguishing causes from consequences when evaluating tau pathology can be addressed by tracking the effects of pathological tau mutations from early in development across the variety of affected cells. Hence, the utility of hCOs. Our article links mutations of the *MAPT* gene to lipid dysregulation and therefore posits that the vector of the disease diathesis projects from tau mutations to dysregulation of the cholesterol pathway. We found increased expression of cholesterol biosynthetic genes in astrocytes and validated these results with lipidomics. Whether the effects of the *MAPT* mutations on astrocytes are indirect or cell autonomous remains open. On one hand, *MAPT* is highly expressed in neurons, making it plausible that the astrocytic change in cholesterol synthesis is a response to impaired neuronal function induced by mutant tau. On the other hand, we detected *MAPT* mRNA in astrocytes, in line with reports of tau protein or mRNA in astrocytes (Kovacs 2020), raising the possibility of cell-autonomous effects, as reported from N279K *MAPT* mutant astrocytes (Hallmann et al., 2017). As we did not observe altered numbers of astrocytes, neurons, or precursors in *MAPT* mutant hCOs (Figure S1D), it seems likely that upregulation of cholesterol biosynthesis in *MAPT* mutant astrocytes is a specific effect and not the by-product of a more general effect such as impaired maturation.

A growing body of evidence links dysregulation of lipid metabolism to AD (Arenas et al., 2017). The most direct link between cholesterol and neurodegeneration is impairment in lysosomal cholesterol transport causing Niemann-Pick Type C disease, characterized by accumulation of cholesterol, other lipids, and tau tangles (Auer et al., 1995; Suzuki et al., 1995). A recent report linked FTD and cholesterol metabolism by showing that plasma cholesterol is increased in FTD (Wang et al., 2020).

Our findings may also be relevant to the emerging role of hyperexcitability in tauopathies, an effect that has also been observed in *MAPT* V337M mutant iPSC-derived neurons (Sohn et al., 2019). As cholesterol can alter membrane properties and modulate synaptic signaling (Korinek et al., 2015, 2020), it is tempting to speculate that elevated cholesterol early on in disease etiology may contribute to hyperexcitability in FTD. Sterol measurements also revealed elevation of cholesterol precursors in *MAPT* mutant hCOs. It is conceivable that these precursors themselves or products derived from them contribute to pathogenesis.

Another top enriched category in genes upregulated in astrocytes of *MAPT* mutant hCOs was fatty acid biosynthesis. Several free polyunsaturated fatty acids, including arachidonic acid, increase tau polymerization (Wilson and Binder 1997; King et al., 2000). Interestingly, the upregulated genes encode three fatty acid desaturases: FADS1, FADS2, and SCD. FADS1 and FADS2 rate limit in fatty acid desaturation and catalyze conversion of linoleic to arachidonic acid. Recently, a genetic *FADS1* variant was shown to increase the levels of arachidonic acid and AD risk (Hammouda et al., 2020). Two other upregulated genes encode enzymes in long-chain saturated fatty acid synthesis: ELOVL6 and FASN. This is intriguing, as long-chain saturated fatty acids are secreted by neurotoxic astrocytes and induce cell death (Guttenplan et al., 2021). ELOVL6 alters the expression of endoplasmic reticulum stress genes (Matsuzaka et al., 2007), known to play a central role in neurodegeneration (Hetz and Saxena 2017).

Astrocytes of *MAPT* mutant hCOs did not show the inflammatory signature of astrocyte activation (Zamanian et al., 2012; Liddelow et al., 2017) or disease-associated astrocytes (Habib et al., 2020). However, *IFITM3*, the only upregulated gene with direct function in the immune response, disrupts cholesterol homeostasis (Amini-Bavil-Olyaee et al., 2013). Furthermore, increased astrocytic IFITM3 expression causes neuronal impairments (Ibi et al., 2013), making it a possibility that IFITM3 functionally connects astrocytic cholesterol dyshomeostasis and neuronal impairment in neurodegeneration.

An important line of evidence for a central role of cholesterol in neurodegeneration was the discovery of the APOE4 allele as the strongest genetic risk factor for late onset AD (Corder et al., 1993), with modulatory effects on tau pathology and tau-related neurodegeneration (Shi et al., 2017). We identified decreased *APOE* transcript levels in astrocytes of *MAPT* mutant hCOs. Expression of astrocytic *APOE* was also decreased in two sc studies of AD (Grubman et al., 2019; Mathys et al., 2019) and in iPSC-derived APOE4 astrocytes versus APOE3 (Lin et al., 2018). Interestingly, a recent study in cerebral hCOs has identified lipidomic changes as a result of *APOE* knockout (Zhao et al., 2021).

The mechanisms of how *MAPT* mutations lead to elevated cholesterol biosynthesis are yet to be determined. One interesting possibility is that the formation of tau oligomers from mutant tau might damage membranes (Flach et al., 2012), and increased cholesterol biosynthesis might be a compensatory response. According to a recent report (Tuck et al., 2022), such elevated membrane cholesterol might in turn protect neurons from tau spread, while cholesteryl esters, on the other hand, play a role in the formation of pathological tau (van der Kant et al., 2019), highlighting the multifaceted role of cholesterol in neurodegeneration. It is also conceivable that cholesterol biosynthesis is increased by toxic tau species via stress signaling.

In summary, our study identifies increased cholesterol biosynthesis downstream of tau mutations. Our findings suggest that astrocytic changes in cholesterol biosynthesis and transport occur in the absence of astrogliosis, severe loss of neurons, and tau inclusions, suggesting that elevated cholesterol biosynthesis precedes these events in the etiology of neurodegeneration caused by *MAPT* mutations. Perturbed cholesterol metabolism is emerging as a shared feature of neurodegenerative diseases, including AD, Parkinson disease, Niemann-Pick Type C, and FTD, and, given our findings, may be an early event in the disease process, warranting further mechanistic investigation with a goal toward early intervention.

## Experimental procedures

iPSCs used in this study were established from the Tau Consortium iPSC line collection (Karch et al., 2019), grown at NSCI core facility NeuraCell, and are available upon request (www.neuralsci.org/tau). hCOs were generated using described protocols (Yoon et al., 2019; Gregory et al., 2020). For scRNA-seq, three to four hCOs were pooled for dissociation, and drop-seq was performed as described (Macosko et al., 2015). Libraries were sequenced on an Illumina Nextseq500 instrument at 50,000 reads per cell. Counts matrices were generated using the Drop-seq tools package (Macosko et al., 2015). Downstream analysis was performed using Seurat 3.0 (Butler et al., 2018; Stuart et al., 2019). The filtered dataset had means of 1,499 transcripts and 930 genes per cell. The mean content of mitochondrially encoded genes was 2.1%. Data were imputed using SAVER (Huang et al., 2018). Free sterol/oxysterol analysis (Quehenberger et al., 2010) of individual cerebral hCOs was performed using LC/MS. See supplemental experimental procedures for details.

## Author contributions

Conceptualization, S.M.K.G., K.S.K., and S.T.; investigation, S.M.K.G., S.K.G., J.N.R., M.A., E.R., G.L., E.K.-R., and A.M.A; methodology, S.M.K.G., S.K.G., J.N.R., S.L., S.B., A.M.A., and O.Q.; formal analysis, S.M.K.G., T.B., E.G., S.J., and A.M.A.; visualization, S.M.K.G. and T.B.; resources, O.Q., S.T., and K.S.K.; software, S.M.K.G. and E.G; supervision, O.Q., S.T., and K.S.K.; project administration, T.B., S.T., and K.S.K.; funding acquisition, S.M.K.G., J.N.R., S.T., and K.S.K.; writing – original draft, S.M.K.G. and K.S.K.; writing – review and editing, J.N.R., M.A., E.G., T.B., S.J., A.M.A., O.Q., and S.T.

## Data Availability

scRNA-seq data are available in NCBI’s GEO under GEO: GSE208418 (https://www.ncbi.nlm.nih.gov/geo/query/acc.cgi?acc=GSE208418). The Seurat object and code used for data analysis were deposited on Dryad under https://doi.org/10.25349/D95898.

## Supplemental information

Document S1. Experimental procedures and Figures S1–S5

Table S1. Marker genes of identified cell populations, related to Figure 1 The reported marker genes were identified using the Wilcoxon rank sum test, with a logFC threshold of 0.25 and Bonferroni correction for multiple testing

Table S2. GO categories enriched in cell population markers, related to Figure 1 Results were obtained by performing GO enrichment analysis on the top 50 markers of each population

Table S3. Lists of differentially expressed genes in MAPT mutant versus control cells, related to Figures 2 and 3 For differential gene expression analyses, the MAST test was used. Genes with Benjamini-Hochberg corrected p-values < 0.05 and logFC > 0.05 or < -0.05 are reported

Document S2. Article plus supplemental information

## References

[bib1] Amini-Bavil-Olyaee S., Choi Y.J., Lee J.H., Shi M., Huang I.C., Farzan M., Jung J.U. (2013). The antiviral effector IFITM3 disrupts intracellular cholesterol homeostasis to block viral entry. Cell Host Microbe.

[bib2] Arenas F., Garcia-Ruiz C., Fernandez-Checa J.C. (2017). Intracellular cholesterol trafficking and impact in neurodegeneration. Front. Mol. Neurosci..

[bib76] Arendt T., Stieler J.T., Holzer M. (2016). Tau and tauopathies. Brain Res. Bull..

[bib3] Ates G., Goldberg J., Currais A., Maher P. (2020). CMS121, a fatty acid synthase inhibitor, protects against excess lipid peroxidation and inflammation and alleviates cognitive loss in a transgenic mouse model of Alzheimer’s disease. Redox Biol..

[bib4] Auer I.A., Schmidt M.L., Lee V.M., Curry B., Suzuki K., Shin R.W., Pentchev P.G., Carstea E.D., Trojanowski J.Q. (1995). Paired helical filament tau (PHFtau) in Niemann-Pick type C disease is similar to PHFtau in Alzheimer’s disease. Acta Neuropathol..

[bib5] Björkhem I. (2013). Cerebrotendinous xanthomatosis. Curr. Opin. Lipidol..

[bib6] Bowles K.R., Silva M.C., Whitney K., Bertucci T., Berlind J.E., Lai J.D., Garza J.C., Boles N.C., Mahali S., Strang K.H. (2021). ELAVL4, splicing, and glutamatergic dysfunction precede neuron loss in MAPT mutation cerebral organoids. Cell.

[bib7] Broe M., Kril J., Halliday G.M. (2004). Astrocytic degeneration relates to the severity of disease in frontotemporal dementia. Brain.

[bib8] Butler A., Hoffman P., Smibert P., Papalexi E., Satija R. (2018). Integrating single-cell transcriptomic data across different conditions, technologies, and species. Nat. Biotechnol..

[bib9] Camp J.G., Badsha F., Florio M., Kanton S., Gerber T., Wilsch-Bräuninger M., Lewitus E., Sykes A., Hevers W., Lancaster M. (2015). Human cerebral organoids recapitulate gene expression programs of fetal neocortex development. Proc. Natl. Acad. Sci. USA.

[bib78] Caspi R., Billington R., Fulcher C.A., Keseler I.M., Kothari A., Krummenacker M., Latendresse M., Midford P.E., Ong Q., Ong W.K., Paley S. (2018). The MetaCyc database of metabolic pathways and enzymes. Nucleic. Acids Res..

[bib10] Clarke B.E., Taha D.M., Tyzack G.E., Patani R. (2021). Regionally encoded functional heterogeneity of astrocytes in health and disease: a perspective. Glia.

[bib11] Corder E.H., Saunders A.M., Strittmatter W.J., Schmechel D.E., Gaskell P.C., Small G.W., Roses A.D., Haines J.L., Pericak-Vance M.A. (1993). Gene dose of apolipoprotein E type 4 allele and the risk of Alzheimer’s disease in late onset families. Science.

[bib12] Cornish-Bowden A. (2013).

[bib13] De Strooper B., Karran E. (2016). The cellular phase of Alzheimer’s disease. Cell.

[bib14] Finak G., McDavid A., Yajima M., Deng J., Gersuk V., Shalek A.K., Slichter C.K., Miller H.W., McElrath M.J., Prlic M. (2015). MAST: a flexible statistical framework for assessing transcriptional changes and characterizing heterogeneity in single-cell RNA sequencing data. Genome Biol..

[bib15] Flach K., Hilbrich I., Schiffmann A., Gärtner U., Krüger M., Leonhardt M., Waschipky H., Wick L., Arendt T., Holzer M. (2012). Tau oligomers impair artificial membrane integrity and cellular viability. J. Biol. Chem..

[bib16] Franzén O., Gan L.M., Björkegren J. (2019). PanglaoDB: a web server for exploration of mouse and human single-cell RNA sequencing data. Database.

[bib77] Fu H., Hardy J., Duff K.E. (2018). Selective vulnerability in neurodegenerative diseases. Nat. Neurosci..

[bib17] Giandomenico S.L., Mierau S.B., Gibbons G.M., Wenger L.M.D., Masullo L., Sit T., Sutcliffe M., Boulanger J., Tripodi M., Derivery E. (2019). Cerebral organoids at the air-liquid interface generate diverse nerve tracts with functional output. Nat. Neurosci..

[bib18] Gonzalez C., Armijo E., Bravo-Alegria J., Becerra-Calixto A., Mays C.E., Soto C. (2018). Modeling amyloid beta and tau pathology in human cerebral organoids. Mol. Psychiatry.

[bib79] Goyal M.S., Vlassenko A.G., Blazey T.M., Su Y., Couture L.E., Durbin T.J., Bateman R.J., Benzinger T.L.S., Morris J.C., Raichle M.E. (2017). Loss of brain aerobic glycolysis in normal human aging. Cell Metab..

[bib19] Gregory J.A., Hoelzli E., Abdelaal R., Braine C., Cuevas M., Halpern M., Barretto N., Schrode N., Akbalik G., Kang K. (2020). Cell type-specific in vitro gene expression profiling of stem cell-derived neural models. Cells.

[bib20] Grubman A., Chew G., Ouyang J.F., Sun G., Choo X.Y., McLean C., Simmons R.K., Buckberry S., Vargas-Landin D.B., Poppe D. (2019). A single-cell atlas of entorhinal cortex from individuals with Alzheimer’s disease reveals cell-type-specific gene expression regulation. Nat. Neurosci..

[bib21] Guttenplan K.A., Weigel M.K., Prakash P., Wijewardhane P.R., Hasel P., Rufen-Blanchette U., Münch A.E., Blum J.A., Fine J., Neal M.C. (2021). Neurotoxic reactive astrocytes induce cell death via saturated lipids. Nature.

[bib22] Habib N., McCabe C., Medina S., Varshavsky M., Kitsberg D., Dvir-Szternfeld R., Green G., Dionne D., Nguyen L., Marshall J.L. (2020). Disease-associated astrocytes in Alzheimer’s disease and aging. Nat. Neurosci..

[bib24] Hallmann A.L., Araúzo-Bravo M.J., Mavrommatis L., Ehrlich M., Röpke A., Brockhaus J., Missler M., Sterneckert J., Schöler H.R., Kuhlmann T. (2017). Astrocyte pathology in a human neural stem cell model of frontotemporal dementia caused by mutant TAU protein. Sci. Rep..

[bib25] Hammouda S., Ghzaiel I., Khamlaoui W., Hammami S., Mhenni S.Y., Samet S., Hammami M., Zarrouk A. (2020). Genetic variants in FADS1 and ELOVL2 increase level of arachidonic acid and the risk of Alzhei’er's disease in the Tunisian population. Prostaglandins Leukot. Essent. Fatty Acids.

[bib26] Hernandez I., Luna G., Rauch J.N., Reis S.A., Giroux M., Karch C.M., Boctor D., Sibih Y.E., Storm N.J., Diaz A. (2019). A farnesyltransferase inhibitor activates lysosomes and reduces tau pathology in mice with tauopathy. Sci. Transl. Med..

[bib27] Hetz C., Saxena S. (2017). ER stress and the unfolded protein response in neurodegeneration. Nat. Rev. Neurol..

[bib28] Huang M., Wang J., Torre E., Dueck H., Shaffer S., Bonasio R., Murray J.I., Raj A., Li M., Zhang N.R. (2018). SAVER: gene expression recovery for single-cell RNA sequencing. Nat. Methods.

[bib29] Ibi D., Nagai T., Nakajima A., Mizoguchi H., Kawase T., Tsuboi D., Kano S.I., Sato Y., Hayakawa M., Lange U.C. (2013). Astroglial IFITM3 mediates neuronal impairments following neonatal immune challenge in mice. Glia.

[bib30] Jiang S., Wen N., Li Z., Dube U., Del Aguila J., Budde J., Martinez R., Hsu S., Fernandez M.V., Cairns N.J. (2018). Integrative system biology analyses of CRISPR-edited iPSC-derived neurons and human brains reveal deficiencies of presynaptic signaling in FTLD and PSP. Transl. Psychiatry.

[bib32] Karch C.M., Kao A.W., Karydas A., Onanuga K., Martinez R., Argouarch A., Wang C., Huang C., Sohn P.D., Bowles K.R. (2019). A comprehensive resource for induced pluripotent stem cells from patients with primary tauopathies. Stem Cell Rep..

[bib33] King M.E., Gamblin T.C., Kuret J., Binder L.I. (2000). Differential assembly of human tau isoforms in the presence of arachidonic acid. J. Neurochem..

[bib34] Korinek M., Vyklicky V., Borovska J., Lichnerova K., Kaniakova M., Krausova B., Krusek J., Balik A., Smejkalova T., Horak M., Vyklicky L. (2015). Cholesterol modulates open probability and desensitization of NMDA receptors. J. Physiol..

[bib35] Korinek M., Gonzalez-Gonzalez I.M., Smejkalova T., Hajdukovic D., Skrenkova K., Krusek J., Horak M., Vyklicky L. (2020). Cholesterol modulates presynaptic and postsynaptic properties of excitatory synaptic transmission. Sci. Rep..

[bib36] Kovacs G.G. (2020). Astroglia and tau: new perspectives. Front. Aging Neurosci..

[bib37] Lancaster M.A., Renner M., Martin C.A., Wenzel D., Bicknell L.S., Hurles M.E., Homfray T., Penninger J.M., Jackson A.P., Knoblich J.A. (2013). Cerebral organoids model human brain development and microcephaly. Nature.

[bib38] Lee H.K., Velazquez Sanchez C., Chen M., Morin P.J., Wells J.M., Hanlon E.B., Xia W. (2016). Three dimensional human neuro-spheroid model of Alzhei’er's disease based on differentiated induced pluripotent stem cells. PLoS One.

[bib39] Liddelow S.A., Guttenplan K.A., Clarke L.E., Bennett F.C., Bohlen C.J., Schirmer L., Bennett M.L., Münch A.E., Chung W.S., Peterson T.C. (2017). Neurotoxic reactive astrocytes are induced by activated microglia. Nature.

[bib40] Lin Y.T., Seo J., Gao F., Feldman H.M., Wen H.L., Penney J., Cam H.P., Gjoneska E., Raja W.K., Cheng J. (2018). APOE4 causes widespread molecular and cellular Alterations associated with Alzhei’er's disease phenotypes in human iPSC-derived brain cell types. Neuron.

[bib41] Long J.E., Cobos I., Potter G.B., Rubenstein J.L.R. (2009). Dlx1&2 and Mash1 transcription factors control MGE and CGE patterning and differentiation through parallel and overlapping pathways. Cereb. Cortex.

[bib44] Macosko E.Z., Basu A., Satija R., Nemesh J., Shekhar K., Goldman M., Tirosh I., Bialas A.R., Kamitaki N., Martersteck E.M. (2015). Highly parallel genome-wide expression profiling of individual cells using nanoliter droplets. Cell.

[bib45] Madison B.B. (2016). Srebp2: a master regulator of sterol and fatty acid synthesis. J. Lipid Res..

[bib46] Mathys H., Davila-Velderrain J., Peng Z., Gao F., Mohammadi S., Young J.Z., Menon M., He L., Abdurrob F., Jiang X. (2019). Single-cell transcriptomic analysis of Alzhei’er's disease. Nature.

[bib47] Matias I., Morgado J., Gomes F.C.A. (2019). Astrocyte heterogeneity: impact to brain aging and disease. Front. Aging Neurosci..

[bib48] Matsuzaka T., Shimano H., Yahagi N., Kato T., Atsumi A., Yamamoto T., Inoue N., Ishikawa M., Okada S., Ishigaki N. (2007). Crucial role of a long-chain fatty acid elongase, Elovl6, in obesity-induced insulin resistance. Nat. Med..

[bib49] Morel L., Chiang M.S.R., Higashimori H., Shoneye T., Iyer L.K., Yelick J., Tai A., Yang Y. (2017). Molecular and functional properties of regional astrocytes in the adult brain. J. Neurosci..

[bib50] Nordberg A., Rinne J.O., Kadir A., Långström B. (2010). The use of PET in Alzheimer disease. Nat. Rev. Neurol..

[bib52] Paşca A.M., Sloan S.A., Clarke L.E., Tian Y., Makinson C.D., Huber N., Kim C.H., Park J.Y., O'Rourke N.A., Nguyen K.D. (2015). Functional cortical neurons and astrocytes from human pluripotent stem cells in 3D culture. Nat. Methods.

[bib53] Polioudakis D., de la Torre-Ubieta L., Langerman J., Elkins A.G., Shi X., Stein J.L., Vuong C.K., Nichterwitz S., Gevorgian M., Opland C.K. (2019). A single-cell transcriptomic atlas of human neocortical development during mid-gestation. Neuron.

[bib54] Quadrato G., Nguyen T., Macosko E.Z., Sherwood J.L., Min Yang S., Berger D.R., Maria N., Scholvin J., Goldman M., Kinney J.P. (2017). Cell diversity and network dynamics in photosensitive human brain organoids. Nature.

[bib55] Quehenberger O., Armando A.M., Brown A.H., Milne S.B., Myers D.S., Merrill A.H., Bandyopadhyay S., Jones K.N., Kelly S., Shaner R.L. (2010). Lipidomics reveals a remarkable diversity of lipids in human plasma. J. Lipid Res..

[bib56] Raja W.K., Mungenast A.E., Lin Y.T., Ko T., Abdurrob F., Seo J., Tsai L.H. (2016). Self-organizing 3D human neural tissue derived from induced pluripotent stem cells recapitulate Alzhei’er's disease phenotypes. PLoS One.

[bib59] Renner M., Lancaster M.A., Bian S., Choi H., Ku T., Peer A., Chung K., Knoblich J.A. (2017). Self-organized developmental patterning and differentiation in cerebral organoids. EMBO J..

[bib60] Saher G., Stumpf S.K. (2015). Cholesterol in myelin biogenesis and hypomyelinating disorders. Biochim. Biophys. Acta.

[bib62] Shi Y., Yamada K., Liddelow S.A., Smith S.T., Zhao L., Luo W., Tsai R.M., Spina S., Grinberg L.T., Rojas J.C. (2017). ApoE4 markedly exacerbates tau-mediated neurodegeneration in a mouse model of tauopathy. Nature.

[bib75] Sidhaye J., Knoblich J.A. (2021). Brain organoids: an ensemble of bioassays to investigate human neurodevelopment and disease. Cell Death Differ.

[bib63] Sloan S.A., Darmanis S., Huber N., Khan T.A., Birey F., Caneda C., Reimer R., Quake S.R., Barres B.A., Paşca S.P. (2017). Human astrocyte maturation captured in 3D cerebral cortical spheroids derived from pluripotent stem cells. Neuron.

[bib64] Sohn P.D., Huang C.T.L., Yan R., Fan L., Tracy T.E., Camargo C.M., Montgomery K.M., Arhar T., Mok S.A., Freilich R. (2019). Pathogenic tau impairs axon initial segment plasticity and excitability homeostasis. Neuron.

[bib65] Stuart T., Butler A., Hoffman P., Hafemeister C., Papalexi E., Mauck W.M., Hao Y., Stoeckius M., Smibert P., Satija R. (2019). Comprehensive integration of single-cell data. Cell.

[bib66] Suzuki K., Parker C.C., Pentchev P.G., Katz D., Ghetti B., D'Agostino A.N., Carstea E.D. (1995). Neurofibrillary tangles in Niemann-Pick disease type C. Acta Neuropathol..

[bib67] Tang B.L. (2020). Glucose, glycolysis, and neurodegenerative diseases. J. Cell. Physiol..

[bib68] Tuck B.J., Miller L.V.C., Katsinelos T., Smith A.E., Wilson E.L., Keeling S., Cheng S., Vaysburd M.J., Knox C., Tredgett L. (2022). Cholesterol determines the cytosolic entry and seeded aggregation of tau. Cell Rep..

[bib31] van der Kant R., Langness V.F., Herrera C.M., Williams D.A., Fong L.K., Leestemaker Y., Steenvoorden E., Rynearson K.D., Brouwers J.F., Helms J.B. (2019). Cholesterol metabolism is a druggable Axis that independently regulates tau and amyloid-β in iPSC-derived Alzhei’er's disease neurons. Cell Stem Cell.

[bib69] Velmeshev D., Schirmer L., Jung D., Haeussler M., Perez Y., Mayer S., Bhaduri A., Goyal N., Rowitch D.H., Kriegstein A.R. (2019). Single-cell genomics identifies cell type-specific molecular changes in autism. Science.

[bib70] Wang P., Zhang H., Wang Y., Zhang M., Zhou Y. (2020). Plasma cholesterol in Alzheimer's disease and frontotemporal dementia. Transl. Neurosci..

[bib71] Wilson D.M., Binder L.I. (1997). Free fatty acids stimulate the polymerization of tau and amyloid beta peptides. In vitro evidence for a common effector of pathogenesis in Alzhei’er's disease. Am. J. Pathol..

[bib72] Yoon S.J., Elahi L.S., Pașca A.M., Marton R.M., Gordon A., Revah O., Miura Y., Walczak E.M., Holdgate G.M., Fan H.C. (2019). Reliability of human cortical organoid generation. Nat. Methods.

[bib73] Zamanian J.L., Xu L., Foo L.C., Nouri N., Zhou L., Giffard R.G., Barres B.A. (2012). Genomic analysis of reactive astrogliosis. J. Neurosci..

[bib74] Zhao J., Lu W., Ren Y., Fu Y., Martens Y.A., Shue F., Davis M.D., Wang X., Chen K., Li F. (2021). Apolipoprotein E regulates lipid metabolism and α-synuclein pathology in human iPSC-derived cerebral organoids. Acta Neuropathol..

