# Supplementary material for: Human tau mutations in cerebral organoids induce a progressive dyshomeostasis of cholesterol
Source: Stem Cell Reports. 2022 Aug 18;17(9):2127–40. doi: 10.1016/j.stemcr.2022.07.011 (PMC9481908; doi:10.1016/j.stemcr.2022.07.011)
Supplement: Document S1. Experimental procedures and Figures S1–S5 [file mmc1.pdf]

**Stem Cell Reports, Volume *17***

## Supplemental Information

# Human tau mutations in cerebral organoids induce a progressive dys-homeostasis of cholesterol

**Stella M.K. Glasauer, Susan K. Goderie, Jennifer N. Rauch, Elmer Guzman, Morgane Audouard, Taylor Bertucci, Shona Joy, Emma Rommelfanger, Gabriel Luna, Erica Keane-Rivera, Steven Lotz, Susan Borden, Aaron M. Armando, Oswald Quehenberger, Sally Temple, and Kenneth S. Kosik**

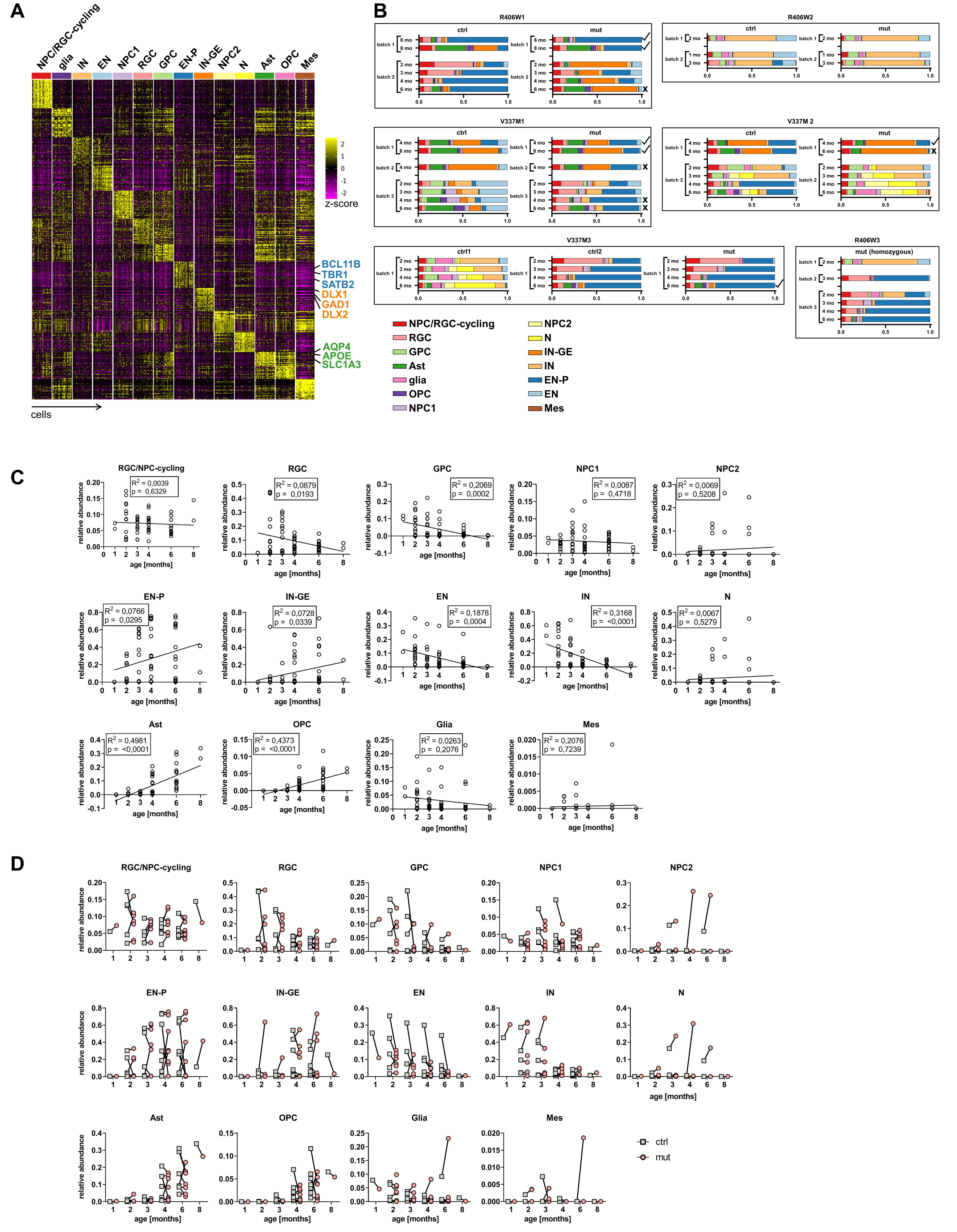


Supplemental Figure 1 – Legend on next page

**Supplemental Figure 1, related to Figure 1 Cellular composition of cerebral organoids A)** Heatmap showing expression levels (z-scores) of the top 50 marker genes for each cell class. Cell classes were randomly downsampled to 50 cells to improve visualization of small clusters. Highlighted are selected canonical markers of EN-P (blue), IN-GE (orange) and Ast (green). **B)** Cellular composition of all individual samples sequenced. For full line names and their abbreviations, see Methods. Shown are relative abundances (number of cells per type relative to all cells in a sample). For isogenic pairs that have ≥ 30 astrocytes in each sample (and were thus further analyzed for transcriptional effects of *MAPT* mutations of astrocytes, Figure 3), it is indicated whether the isogenic pair had > 5% pyramidal neurons (EN-P) and < 50% neurons belonging to the unidentified neuronal populations (IN, EN and N) in each sample of the pair (check mark) or not ("x"). **C)** Changes in relative abundances of cell types over time. Each datapoint represents one individual sample. Simple linear regressions were calculated for each cell type. **D)** Relative abundances of cell types over time, separated by control and mutant samples. Lines were used to visually connect members of isogenic pairs. Statistically significant differences between ctrl and mut samples were calculated for each cell type at ages 2, 3, 4 and 6 months using two-tailed Wilcoxon matched-pairs signed rank tests and Bonferroni correction for multiple testing. All comparisons were non-significant.

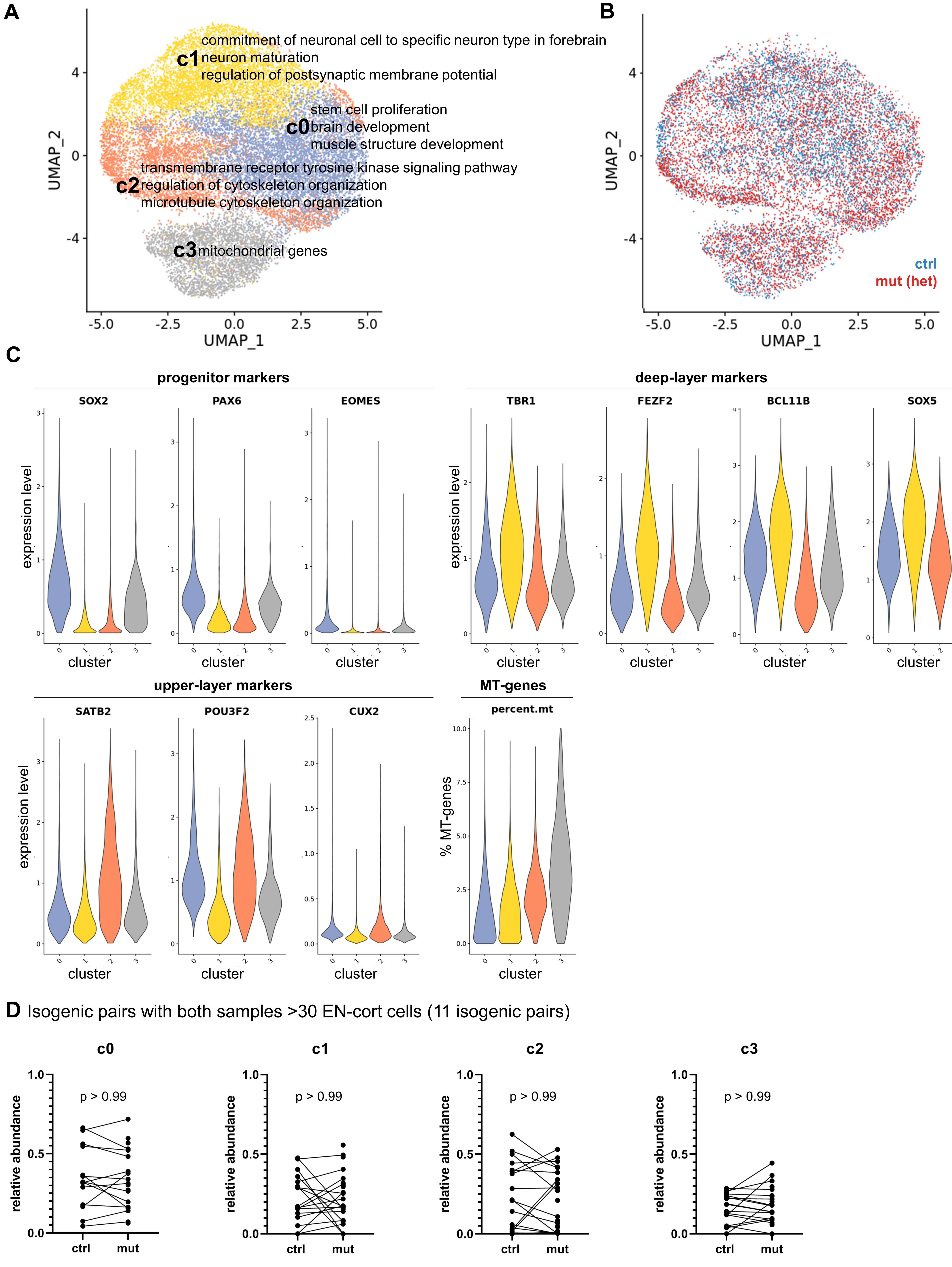


**Supplementary Figure 2 – Legend on next page**

**Supplemental Figure 2, related to Figure 2 Subclustering of pyramidal excitatory neurons A)** UMAP plot of pyramidal excitatory neurons after extraction from entire dataset and subclustering (see Methods), yielding 4 clusters. Gene ontology enrichment analysis using the database "biological process" was performed on the marker genes of each subcluster. Shown are the top 3 enriched gene categories for each cluster. **B)** UMAP colored by control / mutant cells, showing that they are largely intermingled. **C)** Expression values of cortical layer markers specific to progenitors, deep-layer neurons and upper layer neurons, showing enrichment of deep-layer and upper-layer markers in cluster 1 and 2, respectively. Cluster 0 is enriched in progenitor markers. **D)** Analysis of enrichment/depletion of mutant pyramidal excitatory neurons in the identified subclusters. All isogenic pairs of which both members have at least 30 pyramidal neurons were analyzed (11 isogenic pairs) using Wilcoxon matched pairs signed rank test followed by Benjamini-Hochberg correction, returning non-significant p-values. Members of isogenic pairs are connected by lines. Unconnected datapoints are from the homozygous R406W mutation line and were not included in statistics.

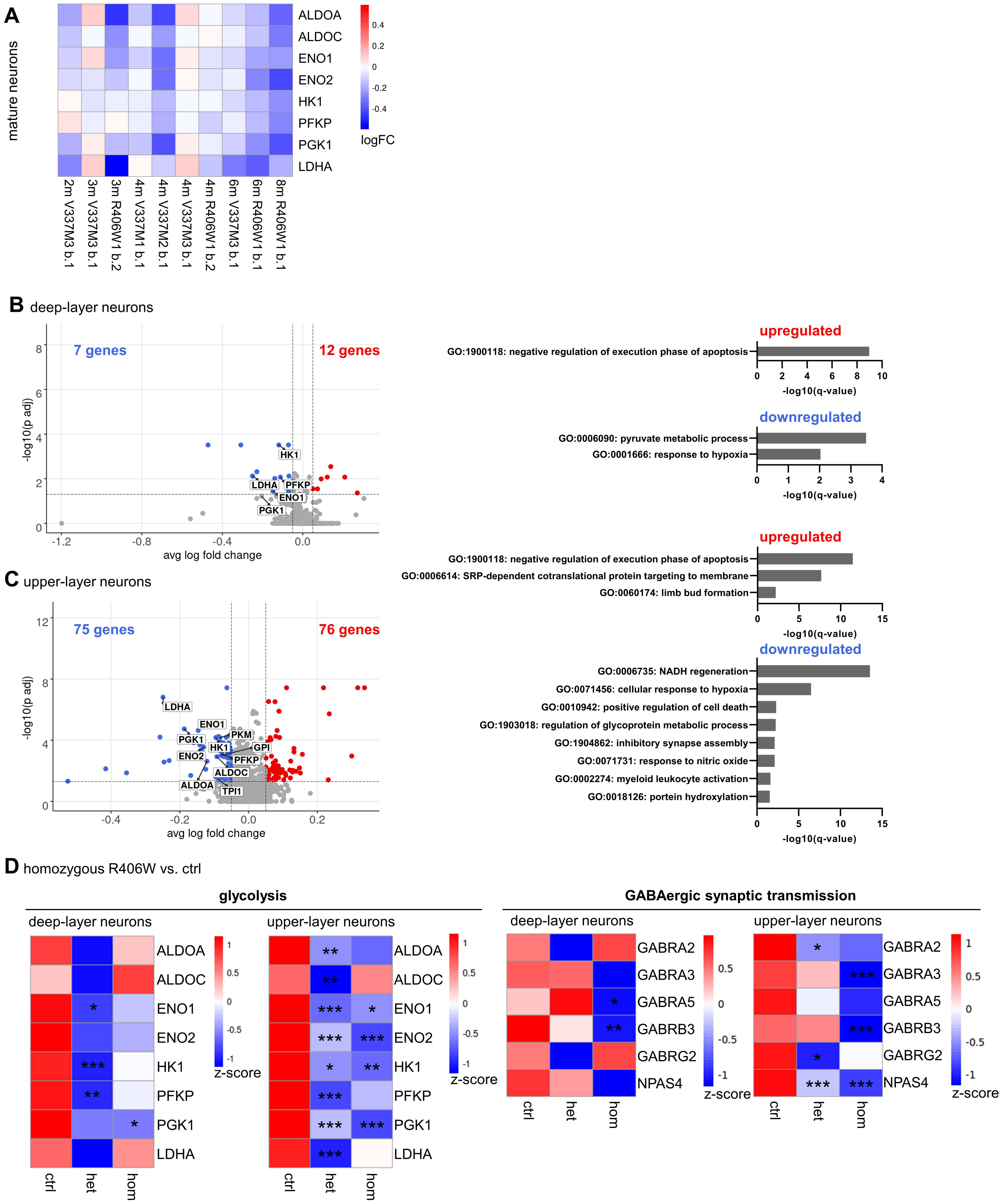


**Supplemental Figure 3 – Legend on next page**

**Supplemental Figure 3, related to Figure 2 Differential gene expression analysis in pyramidal excitatory neurons. A)** Heatmap of logFC of glycolysis related genes identified in Fig. 2C across all isogenic pairs with > 30 pyramidal neurons per sample. Abbreviations b.1 and b.2 refer to the different differentiation batches for each line as depicted in Supplementary Figure 1B. **B)** Differential gene expression analysis in deep-layer neurons (n = 5 isogenic pairs from 4 organoid batches, 1024 cells), using the MAST differential expression test with a logFC cutoff of 0.05. Benjamini-Hochberg corrected p-values < 0.05 were considered statistically significant. Left: volcano plot, differentially expressed genes involved in glycolysis are highlighted; Right: Enriched GO terms ("biological process") of upregulated and downregulated genes. **C)** Differential gene expression analysis in upper-layer neurons (n = 6 isogenic pairs from 4 organoid batches, 1530 cells), using the MAST test as described in (B). Note that *MAPT* mutant neurons of both deep- and upper-layer clusters show enrichment of downregulated genes in categories related to glycolysis (e.g. pyruvate metabolic process, glucose homeostasis, NADH regeneration). **D)** Validation of transcriptional changes observed in heterozygous R406W and V337M mutants in the R406W homozygous mutant line. Genes of the glycolytic pathway (left) and involved in GABAergic synaptic transmission (right) identified as differentially expressed heterozygous mutant vs. ctrl neurons (deep- and upper-layer neurons combined, see Figure 3) are shown. Heatmaps show expression levels (z-scores) of the selected genes in control, heterozygous (V337M and R406W) and homozygous (R406W) mutant cells, separately for deep- and upper-layer neurons enriched clusters. All genes in the dataset were tested for differential expression using the MAST test as described in (B). Significantly different expression compared to controls is indicated: * $p \leq 0.05$, ** $p \leq 0.01$, *** $p \leq 0.001$

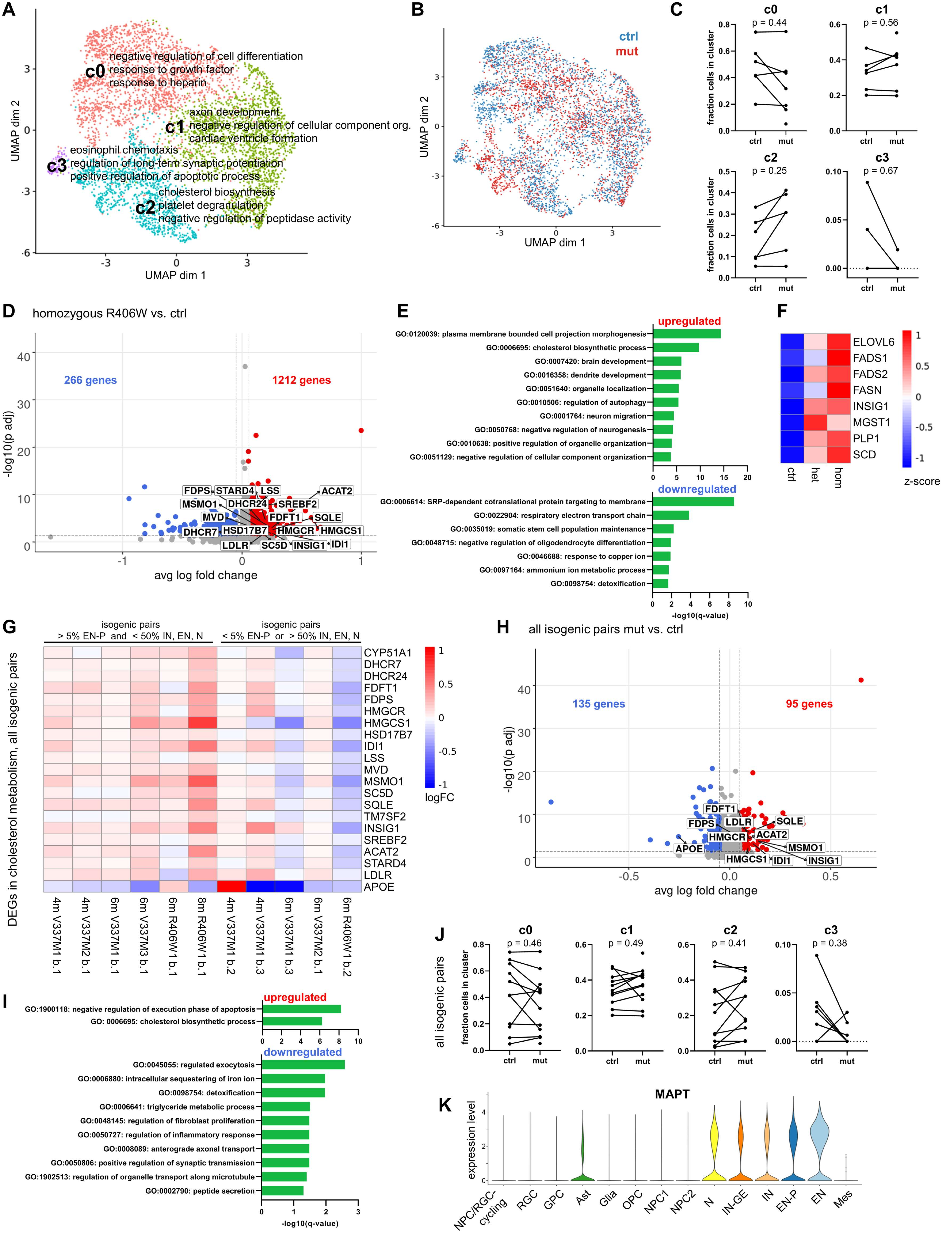


**Supplemental Figure 4 – Legend on next page**

**Supplemental Figure 4, related to Figure 3 Single-cell analysis of astrocytes - extended A)** UMAP of astrocytes that were extracted from the dataset and subclustered (see Methods), resulting in 4 clusters. Gene ontology enrichment analysis using the database "biological process" was performed on the marker genes of each subcluster. Shown are the top 3 enriched gene categories for each cluster. **B)** UMAP colored by control / mutant cells, showing that they are largely intermingled. **C)** Analysis of enrichment/depletion of mutant astrocytes in the subclusters. All isogenic pairs of which both members have at least 30 astrocytes were analyzed (6 isogenic pairs) using Wilcoxon matched pairs signed rank test followed by Benjamini-Hochberg correction. All statistical tests returned non-significant p-values. Members of isogenic pairs are connected by lines. Unconnected datapoints are from the homozygous R406W mutation and were not included in the statistics. **D)** Volcano plot of differential gene expression analysis of astrocytes of the homozygous R406W mutant line vs. ctrl astrocytes (n = 1053 cells), using the MAST test with a logFC cutoff of 0.05. Benjamini-Hochberg corrected p-values < 0.05 were considered statistically significant. Differentially expressed genes involved in cholesterol metabolism are highlighted. **E)** Gene ontology enrichment analysis of genes identified in (D). The GO term "cholesterol biosynthetic process" was enriched in genes upregulated in the homozygous R406W mutant. **F)** Heatmap showing expression levels (z-scores) of genes included in the GO term "fatty acid biosynthetic process" identified as differentially expressed in Figure 3A in control, heterozygous (R406W and V337M) and homozygous (R406W) *MAPT* mutant astrocytes. All genes 8 genes are significantly upregulated in heterozygous mutants compared to control, and in the homozygous mutant compared to control. **G)** Heatmap showing log fold changes of differentially expressed genes that are involved cholesterol metabolism for all individual isogenic pairs. Positive log fold changes are more consistent in the isogenic pairs that have > 5% pyramidal neurons (EN-P) and < 50% neurons belonging to the unidentified neuronal populations (IN, EN and N) in each sample. Abbreviations b.1, b.2 and b.3 refer to the different batches as depicted in Supplementary Figure 1B. **H)** Volcano plot of differential gene expression analysis in all isogenic pairs (n = 11 isogenic pairs from 7 organoid batches, 3398 cells), omitting filtering and using the MAST differential expression test as described in (D). Differentially expressed genes involved in cholesterol metabolism are highlighted. **I)** Gene ontology enrichment analysis of genes identified in (H), demonstrating that the GO term "cholesterol biosynthetic process" was enriched also in genes upregulated when testing the unfiltered dataset for differentially expressed genes. **J)** Analysis for enrichment/depletion of *MAPT* mutant cells as described in (C) but on all isogenic pairs without applying a filter. All statistical tests returned non-significant p-values. **K)** Expression level of *MAPT* mRNA across different cell types, showing presence of *MAPT* mRNA in astrocytes. Unimputed (non-SAVER treated) expression values are shown.

**A**

| Cholesterol and biosynthesis intermediates | | |
|---|---|---|
| abbreviation | full name | detected in n organoids |
| 7-DHC | 7-Dehydrocholesterol | 50 / 60 |
| 8-DHC | 8-Dehydrocholesterol | 60 / 60 |
| DHL | 24,25-Dihydrolanosterol | 60 / 60 |
| T-MAS | 14-Demethyl-lanosterol | 54 / 60 |
| FF-MAS | 14-demethyl-14-dehydrolanosterol | 0 / 60 |
| Dihydro FF-MAS | 4,4-dimethyl-cholest-8(9), 14-dien-3ß-ol | 0 / 60 |
| Dihydro T-MAS | 4,4-dimethyl-cholest-8(9)-en-3ß-ol | 49 / 60 |
| Cholesterol | Cholesterol | 60 / 60 |
| Desmosterol | Desmosterol | 60 / 60 |
| Lanosterol | Lanosterol | 60 / 60 |
| 22-OHC | 22-Hydroxycholesterol, | 0 / 60 |
| Zymostenol | Zymostenol | 0 / 60 |
| Zymosterol | Zymosterol | 0 / 60 |

| Oxysterols | | |
|---|---|---|
| abbreviation | full name | detected in n organoids |
| 24-OHC | 24-Hydroxycholesterol | 32 / 60 |
| 24,25-EpC | 24,25-Epoxycholesterol | 0 / 60 |
| 25-OHC | 25-Hydroxycholesterol | 0 / 60 |
| 27-OHC | 27-Hydroxycholesterol | 10 / 60 |
| 4b-OHC | 4ß-Hydroxycholesterol | 0 / 60 |
| 7a-OHC | 7alpha-Hydroxycholesterol | 0 / 60 |
| 7,25-dOHC | 7,25-Dihydroxycholesterol | 0 / 60 |
| 7,27-dOHC | 7,27-Dihydroxycholesterol | 5 / 60 |
| 7-KetoC | 7-Ketocholesterol | 0 / 60 |

| Other sterols | | |
|---|---|---|
| abbreviation | full name | detected in n organoids |
| 7a-OH-ONE | 7alpha-hydroxy-4-cholesten-3-one | 0 / 60 |
| Cholestanol | Cholestanol | 60 / 60 |
| Campesterol | Campesterol | 60 / 60 |
| Ergosterol | Ergosterol | 0 / 60 |
| Sitosterol | Sitosterol | 54 / 60 |

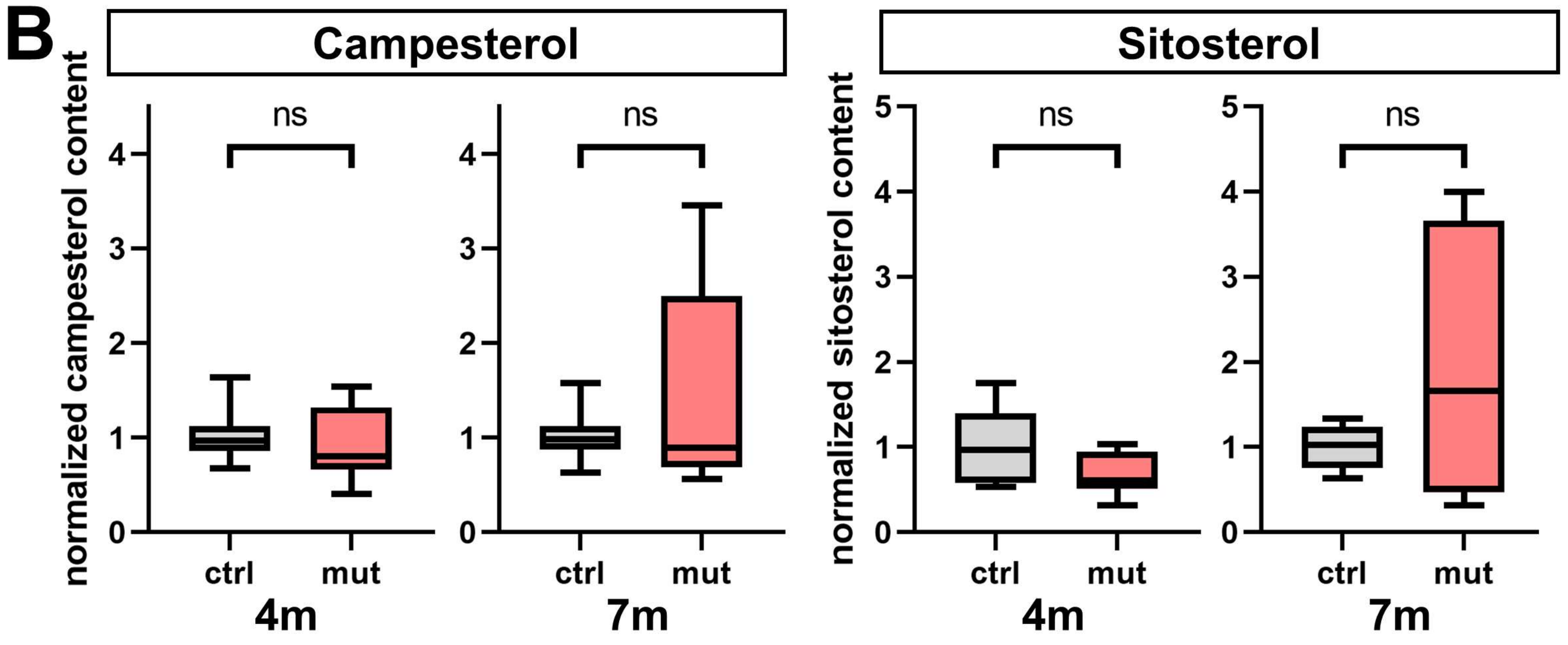


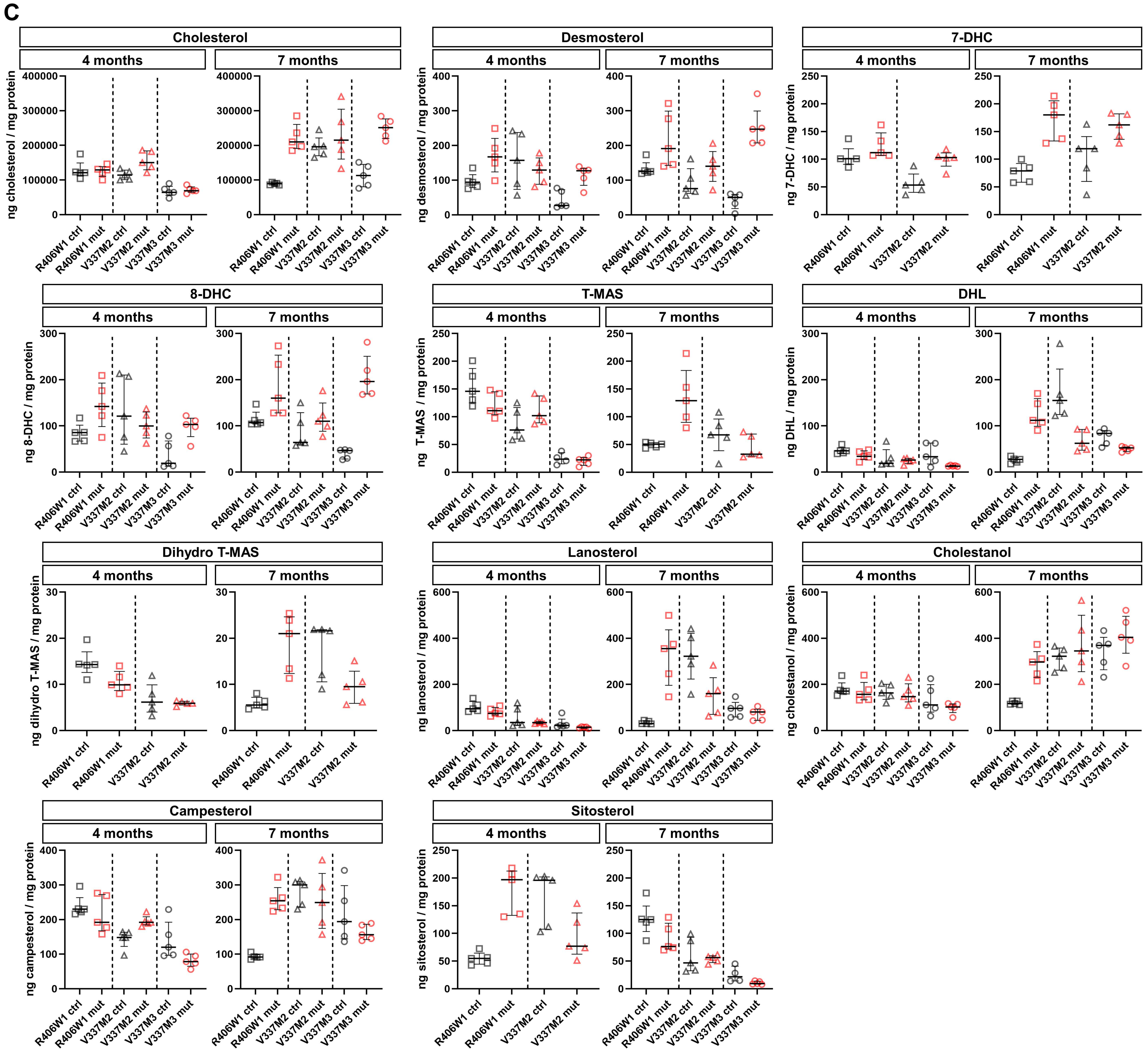


**Supplemental Figure 5, related to Figure 4 Sterol quantification in *MAPT* mutant organoids - extended. A)** List of compounds contained in the sterol panel and their detection rates in organoid samples. **B)** LC-MS data of the two phytosterols detected, all three isogenic lines combined. Sitosterol: 4 months ctrl n = 14 from 3 organoid batches, 4 months mut n = 15 from 3 organoid batches, 7 months ctrl n = 10 from 2 organoid batches, 7 months mut n = 10 from 2 organoid batches. Campesterol: n = 15 from 3 organoid batches for each condition and timepoint. Mut were normalized to ctrl values. Box and whiskers plots represent the median (line in box center), first and third quartile (lower and upper box border, respectively), and minimum and maximum values (whiskers). Wilcoxon rank-sum test followed by Benjamini-Hochberg correction (for 22 multiple comparisons) revealed no statistically significant differences between ctrl and mut. **C)** Representation of LC-MS data for individual isogenic pairs and samples. For full line names, see Methods. Data are expressed at ng / mg protein. Plots display the median (line), first and third quartile ranges (whiskers) and individual data points.

**Supplemental Table Legends**

**Supplemental Table 1, related to Figure 1 Marker genes of identified cell populations.** The reported marker genes were identified using the Wilcoxon rank sum test, with a logFC threshold of 0.25 and Bonferroni correction for multiple testing.

**Supplemental Table 2, related to Figure 1 GO categories enriched in cell population markers.** Results were obtained by performing GO enrichment analysis on the top 50 markers of each population.

**Supplemental Table 3, related to Figures 2 and 3 Lists of differentially expressed genes in *MAPT* mutant versus control cells.** For differential gene expression analyses, the MAST test was used. Genes with Benjamini-Hochberg corrected p-values < 0.05 and logFC > 0.05 or < -0.05 are reported.

**Supplemental Experimental Procedures**

**Cell Lines and Maintenance of Human Induced Pluripotent Stem Cells (iPSCs)**

The iPSC lines used in this study: GIH6-1-C1Δ1E11 (WT/WT), GIH6-1-C1Δ1A02 (V337M/WT), GIH7-C2Δ2B12 (WT/WT), GIH7-C2Δ2F02 (WT/WT), GIH7-C2Δ2A01 (V337M/WT), ND32951A.15Δ1B06 (WT/WT), and ND32951A.15Δ1B09 (V337M/WT), GIH143-C3 (R406W/R406W, homozygote), F11362.1ΔC11 (WT/WT), F11362.1ΔF10 (R406W/WT), F11421.12Δ2A07 (WT/WT), F11421.12 (R406W/WT), were established from the Tau Consortium iPSC line collection (Karch et al. 2019) and grown at NSCI core facility NeuraCell and are available upon request (www.neuralsci.org/tau). Short names for the isogenic pairs were used throughout the manuscript as follows: R406W1: F11362.1ΔC11 (WT/WT) and F11362.1ΔF10 (R406W/WT); R406W2: F11421.12Δ2A07 (WT/WT) and F11421.12 (R406W/WT); V337M1: ND32951A.15Δ1B06 (WT/WT), and ND32951A.15Δ1B09 (V337M/WT); V337M2: GIH6-1-C1Δ1E11 (WT/WT) and GIH6-1-C1Δ1A02 (V337M/WT); V337M3: GIH7-C2Δ2B12 (WT/WT), GIH7-C2Δ2F02 (WT/WT) and GIH7-C2Δ2A01 (V337M/WT). APOE genotypes are as follows. E3/E3 genotype: GIH6-1-C1Δ1E11, GIH6-1-C1Δ1A02, GIH7-C2Δ2B12, GIH7-C2Δ2F02, GIH7-C2Δ2A01, GIH143-C3, F11421.12Δ2A07 and F11421.12; E4/E4 genotype: ND32951A.15Δ1B06 and ND32951A.15Δ1B09; E2/E3 genotype: F11362.1ΔC11 and F11362.1ΔF10. The iPSC lines were expanded with daily feeding of mTeSR1 medium (StemCell Technologies, catalog #05851) or mTESR1 with FGF2-Discs (StemCultures catalog #DSC500) and feeder-free conditions in six-well plates (Corning, catalog #3506) precoated with growth factor-reduced Matrigel (Corning, catalog #356231). iPSCs were passaged every week using Dispase (STEMCELL Technologies Cat#07923) or ReLeSR (Stem Cell Tech catalog #100-0484).

**3D Cerebral Organoid Production**

Following previously described protocols (Yoon et al. 2019; Gregory et al. 2020), organoid production was started in AggreWell™800 plates (Stem Cell Technologies, catalog #34811). The wells were rinsed with 2 ml of DMEM/F12/well, then aspirated. 0.5 ml of organoid formation medium (Essential 8 (E8) medium (ThermoFisher, cat. #A1517001) supplemented with ROCK inhibitor - Y-27632 (Tocris Cat#1254) diluted1:1000 to 10 µM) was added to each well. The plates were centrifuged at 2,000 x g for 5 minutes in a swinging bucket rotor fitted with a plate holder and inspected under a microscope to ensure any bubbles were removed. iPSCs were grown to 80-85% confluency. To obtain a single-cell suspension of iPSCs for effective organoid formation, the cells were pre-treated with 10 µM of Y-27632 in E8 medium

for 50-60 minutes. The culture medium was removed from the wells, which were rinsed twice with DPBS (Dulbecco's Phosphate Buffered Solution without Calcium or Magnesium: Gibco, cat. #14190-144). 2 ml of Accutase were added to each well and incubated for c.10 minutes at 37°C in 5% $CO_2$ until cells lifted off the dish by gentle shaking. The cell suspension was gently triturated 2-3 times to dissociate remaining cell clumps. The cells were counted, centrifuged at 1,200 rpm for 4 minutes, and resuspended in organoid formation medium to achieve a single-cell suspension of 3 million cells in 1.5 ml which was added to each AggreWell to give a final volume of 2 ml per well. The suspension was gently pipetted to evenly distribute cells throughout each well. The plate was centrifuged for 3 minutes at 100xg to dispense the cells into the microwell cones to achieve even distribution, which was checked under the microscope. The cells were incubated for 24 hours at 37°C and 5% $CO_2$.

After 24 hours of incubation, the 3D organoids were removed from the AggreWell™800 plate, rinsed twice in DMEM/F12 medium then transferred into ultra-low attachment 10 cm plates in medium A: E6 medium supplemented with Dorsomorphin (DM) (Tocris, catalog #3093) at 2.5 µM;SB431542 (Tocris, catalog #1614) at 10 µM, and XAV-939 (Tocris #3748) at 2.5 µM.

After day 1, medium A changes were performed daily. On day 6, the medium was changed to medium B: Neurobasal-A (Life Technologies #10888-022) supplemented with B-27 supplement without vitamin A (Life Technologies, catalog #12587010), Anti-A (Life Technologies, catalog #15240-062), 20 ng/ml FGF2 (R&D Systems, catalog #233-FB), GlutaMax (Life Technologies, catalog #3505-061), and 20 ng/ml EGF (Peprotech, catalog # AF-100-15). The organoids were fed with medium B, daily for the first 10 days and every other day for the subsequent 9 days. Starting at day 25, medium C was used: Neurobasal-A (Life Technologies #10888-022) supplemented with B-27 supplement without vitamin A (Life Technologies, catalog #12587010), Anti-A (Life Technologies, catalog #15240-062), GlutaMax(Life Technologies, catalog #3505-061), 20 ng/ml BDNF (Peprotech, catalog # 450-02) and 20 ng/ml NT3 (Peprotech, catalog # 450-03), every other day. From day 43 onwards, medium D was used: the same as C but without BDNF or NT3 and changed every four days. Throughout the culture period, organoids that fused together were separated by cutting with a disposable scalpel (McKesson non-safety scalpels, 1626). Organoids in Figures 4, iPSCs were grown and patterned as described above with minor modifications, culturing in ultra-low attachment 96 well U-bottom plates (S-BIO, catalog #MS9096SZ).

## Single-cell RNA Sequencing of Cerebral Organoids

### Organoid Samples

For the V337M mutant line GIH7 (GIH7-C2Δ2A01), organoids of two different CRISPR-corrected clones (GIH7-C2Δ2B12 and GIH7-C2Δ2F02) were sequenced. For all other heterozygous mutant lines (F11362.1.ΔF10, F11421.12parent, ND32951A.15Δ2B09 and GIH6-1-C1ΔA02), one CRISPR corrected clone each was used as control line (F11362.1.ΔC11, F11421.12ΔA07, ND32951A.15Δ1B06 and GIH6-1-C1ΔE11). We also sequenced organoids from a homozygous carrier of the R406W mutation (GIH143-C3). We sampled organoids at ages 2, 3, 4 and 6 months, except for the R406W mutant line F11421 which was sampled up to 3 months. One isogenic pair (R406W mutant F11362.1.ΔF10 and isogenic control F11362.1.ΔC11) was also sampled at 8 months, and one isogenic pair (R406W mutant F11421.12parent and isogenic control F11421.12ΔA07) at 1 month (Figure 1B). One replicate (defined as one differentiation experiment) of each line at each timepoint was sequenced, with a few exceptions where we sequenced 2 or 3 replicates (Figure 1B).

### Organoid Dissociation

3-4 organoids were pooled for each dissociation and dissociated using the Worthington Papain Dissociation System (Worthington). Solutions were prepared according to the Manufacturer's instructions. Organoids in FB "D" media were cut into pieces with a sterile scalpel blade and washed twice in PBS prior to dissociation. Incubation in papain/DNaseI solution was performed in a 15 ml conical tube with untightened lid and at 37°C and 5% $CO_2$ with occasional agitation. After the first 30 min of incubation, organoids were triturated 10 times with a fire-polished glass Pasteur pipette, followed by further incubation and trituration every 15 min until dissociation was complete. The total incubation time in papain/DNaseI solution was 75 -90 min. After adding Inhibitor Solution and DNaseI in EBSS, the dissociated cells were pelleted by centrifugation for 5 min at 300 g. The cells were resuspended in ice-cold PBS/0.01% BSA and passed through a 35 µm cell strainer. An aliquot of the single-cell suspension was mixed with Trypan blue and analyzed for viability and cell count. Viability was > 90% for all organoid samples.

### Drop-seq Library Construction

Following dissociation, cells were diluted to 110 cells/µl in PBS/0.01% BSA. Drop-seq was performed as described (Macosko et al., 2015, full details available under http://mccarrolllab.org/download/905/). Briefly, cells and barcoded beads (Chemgenes, 132 beads/ul in lysis buffer) were ran on an aquapel-treated microfluidic drop-seq device (FlowJEM) for co-encapsulation in nanoliter-sized droplets. After

droplet-breakage, reverse transcription and Exonuclease I treatment, beads were counted, and 2000 beads were apportioned per PCR tube for cDNA amplification. The amplified cDNA libraries were purified using SPRI beads (Beckman Coulter) and quantified on a Fragment Analyzer (Agilent). Tagmentations were performed using the Illumina Nextera XT kit (Illumina), and the resulting libraries were purified in two consecutive rounds of SPRI beads-based size selection (0.6x beads to sample ratio followed by 1x beads to sample ratio). The size and concentration of the final libraries were measured on a Fragment Analyzer and a Qbit Fluorometer (ThermoFisher), respectively. Libraries were sequenced on an Illumina Nextseq500 instrument at 50,000 reads per cell.

**Single-cell RNA Data Analysis of Cerebral Organoids**

**Counts Matrix Generation**

Counts matrices were generated using the Drop-seq tools package (Macosko et al. 2015), with full details available online (https://github.com/broadinstitute/Drop-seq/files/2425535/Drop-seqAlignmentCookbookv1.2Jan2016.pdf). Briefly, raw reads were converted to BAM files, cell barcodes and UMIs were extracted, and low-quality reads were removed. Adapter sequences and polyA tails were trimmed, and reads were converted to Fastq for STAR alignment (STAR version 2.6). Mapping to human genome (hg19 build) was performed with default settings. Reads mapped to exons were kept and tagged with gene names, beads synthesis errors were corrected, and a digital gene expression matrix was extracted from the aligned library. We extracted data from twice as many cell barcodes as the number of cells targeted (NUM_CORE_BARCODES = 2x # targeted cells).

**Filtering Low-Quality Cells and Doublets**

Downstream analysis was performed using Seurat 3.0 (Butler et al. 2018; Stuart et al. 2019) in R version 3.6.3. An individual Seurat object was generated for each sample which were filtered and clustered individually. This is because the Doublet Finder method (see below) identifies doublets more accurately in individual samples when they have different cellular compositions. Cells with < 300 genes detected were filtered out, as were cells with > 10% mitochondrial gene content. Counts data were log-normalized using the default NormalizeData function and the default scale of 1e4. Then, the top 2000 variable genes were identified using the Seurat FindVariableFeatures function (selection.method = “vst”, nfeatures = 2000), followed by scaling and centering using the default ScaleData function. Principal Components Analysis was carried out on the scaled expression values of the 2000 top variable genes, and the cells were clustered using the first 50 principal components (PCs) as input in the FindNeighbors function, and a resolution of 0.4 in the FindClusters function. Non-linear dimensionality reduction was performed by

running UMAP on the first 50 PCs. Following clustering and dimensionality reduction, putative cell doublets were identified using DoubletFinder (McGinnis et al. 2019), assuming a doublet formation rate of 5%. For each sample, the optimal pK value was identified based on the results of paramSweep_vs, summarizeSweep and find.pK functions of the DoubletFinder package. Instead of using the default paramSweep_vs function, we extended the upper range of computed pK values to 1.2. We visually verified cells identified as doublets had high nFeatures (number of genes expressed) by plotting the pANN metric against nFeatures. For samples not showing this correlation, we adjusted the pK value to the next highest peak in the pK/BCmetric plot. Finally, the individual Seurat objects were merged, and doublets removed. The resulting dataset had a mean of 1499 transcripts per cell and 930 genes per cell. The mean content of mitochondrially-encoded genes was 2.1%.

### Data Imputation

Raw counts of the merged Seurat object were extracted using the GetAssayData function (slot = "counts"). Imputation was performed using the saver Function of the SAVER package (Huang et al. 2018) with default settings. A new Seurat object was generated from the resulting matrix.

### Clustering and Cell Type Identification

Normalization, variable gene selection, and scaling on the imputed dataset were performed as above with the exception that 3000 variable genes were identified. Statistically significant principal components were determined using the JackStraw and ScoreJackstraw functions of Seurat v3, revealing a drop-off of p-values below significance level of 0.05 between PCs 90-100. Consequently, clustering and UMAP dimensionality reduction were performed on the first 90 PCs. Clustering at resolution 0.6 resulted in 17 clusters in good agreement with the expression of known marker genes for cell types found in brain organoids. Marker genes for each cluster were identified using the Wilcoxon rank sum test implemented in the FindMarkers function, with a logFC threshold of 0.25 and Bonferroni correction for multiple testing.

Marker genes for each cluster were manually compared to known marker genes, and to published single-cell data of the human brain and brain organoids (Giandomenico et al. 2019; Polioudakis et al. 2019; Quadrato et al. 2017; Velmeshev et al. 2019). Clusters 1 and 10 were merged into the "IN" cluster (inhibitory neurons most likely not derived from ganglionic eminences), since they show very low expression of ganglionic eminences markers (DLX1,2,5,6 and DLX6-AS1), but share GAD1 as marker gene. Similarly, clusters 3, 4, and 5 were merged to form the "EN-P" cluster (excitatory cortical neurons), based on the common expression of marker genes BCL11B, TBR1 and SOX5. This merging of clusters resulted in 14 major subpopulations. The list of marker genes for each cluster is provided as Supplementary Table 1.

### Subclustering of Astrocytes and Pyramidal Glutamatergic Neurons

The astrocyte cluster was extracted from the dataset using Seurat's subset function. Variable features were selected using the "mvp" method and default parameters. Clustering and UMAP projection were performed as above, using the first 50 PCs as input. The same procedure was applied to the EN-P population but using the first 70 PCs for clustering and UMAP projection.

### GO Enrichment Analyses

Metascape (Zhou et al. 2019), available at https://metascape.org was used for all gene ontology enrichment analyses, with the following custom settings: A background gene list of the 22,097 genes in our dataset was provided, and gene enrichment was performed on the GO Biological Processes database. Metascape performs hierarchical clustering of the enriched terms, and the most significant term within a cluster was selected as the representative term to report in GO enrichment results. The representative GO terms were filtered using a Q-value cutoff of 0.05.

### Differential Gene Expression Analyses

Before testing for differentially expressed genes between control and *MAPT* mutant cells, the number of control and mutant cells were balanced for each isogenic pair, i.e. the sample with the higher number of cells was subsetted to the number of cells in the sample with the lower number of cells. This was done to prevent differences between cell lines or batches from confounding the analyses. For comparisons to the V337M mutant cell line GIH7-C2Δ2A01, the isogenic control GIH7-C2Δ2F02 was used and GIH7-C2Δ2B12 was disregarded due to an almost complete lack of pyramidal neurons (EN-P) and abundance of unknown neuron (IN and N) categories (Supplementary Figure 1B). All differential gene expression analyses of *MAPT* mutant vs. control cells were carried out using the MAST test (Finak et al. 2015) as implemented in the Seurat package with a logFC cutoff of 0 and a minimum percentage of 0 (logFC.cutoff = 0, min.pct = 0). Benjamini-Hochberg corrected p-values < 0.05 were considered statistically significant. A logFC cutoff of 0.05 was applied after differential gene expression testing to identify genes of interest. Packages used to visualize results were pheatmap (Kolde 2019) and EnhancedVolcano (Blighe, Rana, and Lewis 2019).

### Gene Signature Scoring

Gene signature scoring was performed using Seurat's AddModuleScore function with default parameters and a bin size of 8. Scoring was performed on the gene signature "superpathway of cholesterol biosynthesis", deposited in the Human Cyc database and comprising 25 genes (ACAT1, ACAT2, CYP51A1,

DHCR24, DHCR7, EBP, FDFT1, FDPS, GGPS1, HMGCR", HMGCS1, HMGCS2, HSD17B7, IDI1, IDI2, LBR, LSS, MSMO1, MVD, MVK, NSDHL, PMVK, SC5D, SQLE, TMSF2).

**Immunohistochemistry of Organoid Sections**

Immunohistochemistry was performed on isogenic lines from 3 different mutation carriers (R406W1, V337M2, V337M3), and in one or two differentiation batches per line (R406W1: 2 batches, V337M2: 2 batches, V337M3: 1 batch using ctrl2 as isogenic control, see "Cell Lines and Maintenance of Human Induced Pluripotent Stem Cells (iPSCs)" subsection for full line names. 3-4 organoids were evaluated for co-expression of HMGCS1 and GFAP for each cell line and condition.

Organoids were fixed overnight in 4% paraformaldehyde (Santa Cruz), sunk in 30% sucrose, embedded in OCT compound and rapidly frozen before tissue was cryosectioned at 20 µm thickness, with a Leica cryostat model CM3050. All solutions were used at approximately 500uL/slide. Slides were thawed to room temperature (RT), blocked in 10% Normal Goat Serum (NGS), 3% Bovine Serum Albumin (BSA), 0.3% Triton-X 100 in phosphate buffered saline (PBS) for 1 hour at RT. Slides were incubated with anti-HMGCS1 (Abcam ab155787) 1:100 in block solution overnight at 4$^{o}$C. Sections were then incubated with goat anti-rabbit IgG-488 (Invitrogen) antibody diluted 1:1000 in block solution for 1 hour at RT. Slides were co-labeled with GFAP (Millipore MAB-3402) 1:400 in block overnight at 4$^{o}$C. GFAP was revealed by incubating with secondary antibody Goat anti mouse $IgG_1$-546 (Invitrogen) at 1:1000 in block for 1 hour at RT. Slides were incubated with DAPI (Sigma D-1306) at 1:1000 in PBS 10 min at RT to label nuclei. High power images were taken with a Zeiss Axio-Observer Z1 epifluorescence microscope. GFAP+ cells that were co-stained for HMGCS1 were quantified. Images of 4 sections per organoid were taken; Sections were randomly chosen to be representative of the whole organoid - top to bottom and left to right.

**Lipidomics**

For free sterol/oxysterol analysis, individual snap-frozen cerebral organoids were homogenized into 200uL of 10% methanol in water. An internal standard mix of 25-Hydroxycholesterol-d6, Desmosterol-d6, and Campesterol-d6 (Avanti Polar Lipids) was added to 180uL of homogenate. Samples were extraction using a modified BUME extraction (Löfgren et al., 2016). Extracts were brought to dryness and taken up in 90% methanol in water and run on a Waters Acquity UPLC interfaced with an AB Sciex 6500 QTrap mass spectrometer equipped with an APCI probe. Source settings were: Curtain Gas=20, Collision Gas=Medium,

Ion Spray Voltage=5500, Temperature=400, GS1=25, GS2=15. A Phenomenex Kinetex C18 1.7uM 2.1mm x 150mm column was used for chromatographic separation. A 30min step gradient was employed using 70/30 Acetonitrile/Water with 5mM Ammonium Acetate as Buffer A and 50/50 acetonitrile/water with 5mM Ammonium Acetate as Buffer B with a flow of 0.5 mL/min. The gradient started at 0%B for 2 minutes, ramped to 10%B over 4 minutes, 15%B over 9 minutes, 50%B over 11 minutes, 100%B over 2 minutes, then held at 100%B for 2 minutes. Sterol species were identified by mass spectrometry on a 6500 Qtrap instrument (Sciex) using 30 MRMs (Multiple Reaction Monitoring) in positive mode. Standard curves were obtained in parallel using identical conditions. Data analysis was performed with Analyst and Mulitquant software packages.

**Supplemental References**


Butler, A., Hoffman, P., Smibert, P., Papalexi, E., and Satija, R. (2018). Integrating single-cell transcriptomic data across different conditions, technologies, and species. *Nat Biotechnol*, *36*(5), 411–420. 10.1038/nbt.4096

Finak, G., McDavid, A., Yajima, M., Deng, J., Gersuk, V., Shalek, A. K., Slichter, C. K., Miller, H. W., McElrath, M. J., Prlic, M., et al. (2015). MAST: a flexible statistical framework for assessing transcriptional changes and characterizing heterogeneity in single-cell RNA sequencing data. *Genome Biol*, *16*, 278. 10.1186/s13059-015-0844-5

Giandomenico, S. L., Mierau, S. B., Gibbons, G. M., Wenger, L., Masullo, L., Sit, T., Sutcliffe, M., Boulanger, J., Tripodi, M., Derivery, E., et al. (2019). Cerebral organoids at the air-liquid interface generate diverse nerve tracts with functional output. *Nat Neurosci*, *22*(4), 669–679. 10.1038/s41593-019-0350-2

Gregory, J. A., Hoelzli, E., Abdelaal, R., Braine, C., Cuevas, M., Halpern, M., Barretto, N., Schrode, N., Akbalik, G., Kang, K., et al. (2020). Cell Type-Specific In Vitro Gene Expression Profiling of Stem Cell-Derived Neural Models. *Cells*, *9*(6), 1406. 10.3390/cells9061406

Huang, M., Wang, J., Torre, E., Dueck, H., Shaffer, S., Bonasio, R., Murray, J. I., Raj, A., Li, M., and Zhang, N. R. (2018). SAVER: gene expression recovery for single-cell RNA sequencing. *Nat Methods*, *15*(7), 539–542. 10.1038/s41592-018-0033-z

Karch, C. M., Kao, A. W., Karydas, A., Onanuga, K., Martinez, R., Argouarch, A., Wang, C., Huang, C., Sohn, P. D., Bowles, K. R., et al. (2019). A Comprehensive Resource for Induced Pluripotent Stem Cells from Patients with Primary Tauopathies. *Stem Cell Reports*, *13*(5), 10.1016/j.stemcr.2019.09.006

Löfgren, L., Forsberg, G. B., and Ståhlman, M. (2016). The BUME method: a new rapid and simple chloroform-free method for total lipid extraction of animal tissue. *Sci Rep*, *6*, 27688. 10.1038/srep27688

Macosko, E. Z., Basu, A., Satija, R., Nemesh, J., Shekhar, K., Goldman, M., Tirosh, I., Bialas, A. R., Kamitaki, N., Martersteck, E. M., et al. (2015). Highly Parallel Genome-wide Expression Profiling of Individual Cells Using Nanoliter Droplets. *Cell*, *161*(5), 1202–1214. 10.1016/j.cell.2015.05.002

McGinnis, C. S., Murrow, L. M., and Gartner, Z. J. (2019). DoubletFinder: Doublet Detection in Single-Cell RNA Sequencing Data Using Artificial Nearest Neighbors. *Cell Syst*, *8*(4), 329–337.e4. 10.1016/j.cels.2019.03.003

Polioudakis, D., de la Torre-Ubieta, L., Langerman, J., Elkins, A. G., Shi, X., Stein, J. L., Vuong, C. K., Nichterwitz, S., Gevorgian, M., Opland, C. K., et al. (2019). A Single-Cell Transcriptomic Atlas of Human Neocortical Development during Mid-gestation. *Neuron*, *103*(5), 785–801.e8. 10.1016/j.neuron.2019.06.011

Quadrato, G., Nguyen, T., Macosko, E. Z., Sherwood, J. L., Min Yang, S., Berger, D. R., Maria, N., Scholvin, J., Goldman, M., Kinney, J. P., et al. (2017). Cell diversity and network dynamics in photosensitive human brain organoids. *Nature*, *545*(7652), 48–53. 10.1038/nature22047

Stuart, T., Butler, A., Hoffman, P., Hafemeister, C., Papalexi, E., Mauck, W. M., 3rd, Hao, Y., Stoeckius, M., Smibert, P., and Satija, R. (2019). Comprehensive Integration of Single-Cell Data. *Cell*, *177*(7), 1888–1902.e21. 10.1016/j.cell.2019.05.031

Velmeshev, D., Schirmer, L., Jung, D., Haeussler, M., Perez, Y., Mayer, S., Bhaduri, A., Goyal, N., Rowitch, D. H., and Kriegstein, A. R. (2019). Single-cell genomics identifies cell type-specific molecular changes in autism. *Science*, *364*(6441), 685–689. 10.1126/science.aav8130

Yoon, S. J., Elahi, L. S., Pașca, A. M., Marton, R. M., Gordon, A., Revah, O., Miura, Y., Walczak, E. M., Holdgate, G. M., Fan, H. C., et al. (2019). Reliability of human cortical organoid generation. *Nat Methods*, *16*(1), 75–78. 10.1038/s41592-018-0255-0

Zhou, Y., Zhou, B., Pache, L., Chang, M., Khodabakhshi, A. H., Tanaseichuk, O., Benner, C., and Chanda, S. K. (2019). Metascape provides a biologist-oriented resource for the analysis of systems-level datasets. *Nat Commun*, *10*(1), 1523. 10.1038/s41467-019-09234-6
