# Supplementary material for: Human tau mutations in cerebral organoids induce a progressive dyshomeostasis of cholesterol
Source: Stem Cell Reports. 2022 Aug 18;17(9):2127–40. doi: 10.1016/j.stemcr.2022.07.011 (PMC9481908; doi:10.1016/j.stemcr.2022.07.011)
Supplement: Document S2. Article plus supplemental information [file mmc5.pdf]

Stem Cell Reports
Article
OPEN ACCESS

Human tau mutations in cerebral organoids induce a progressive dyshomeostasis of cholesterol

Stella M.K. Glasauer,[1] Susan K. Goderie,[2] Jennifer N. Rauch,[1,4] Elmer Guzman,[1,5] Morgane Audouard,[1,5] Taylor Bertucci,[2] Shona Joy,[2] Emma Rommelfanger,[1] Gabriel Luna,[1] Erica Keane-Rivera,[1] Steven Lotz,[2] Susan Borden,[2] Aaron M. Armando,[3] Oswald Quehenberger,[3] Sally Temple,[2,*] and Kenneth S. Kosik[1,*]
[1]Neuroscience Research Institute and Department of Molecular, Cellular and Developmental Biology, University of California, Santa Barbara, Santa Barbara, CA 93106, USA
[2]Neural Stem Cell Institute, Rensselaer, NY 12144, USA
[3]Department of Pharmacology, University of California, San Diego, San Diego, CA 92093, USA
[4]Present address: Department of Biochemistry and Molecular Biology, University of Massachusetts Amherst, Amherst, MA 01003, USA
[5]Present address: Altos Labs Bay Area Institute of Science, Redwood City, CA 94065, USA
*Correspondence: sallytemple@neuralsci.org (S.T.), kosik@lifesci.ucsb.edu (K.S.K.)
https://doi.org/10.1016/j.stemcr.2022.07.011

SUMMARY

Mutations in the *MAPT* gene that encodes tau lead to frontotemporal dementia (FTD) with pathology evident in both cerebral neurons and glia. Human cerebral organoids (hCOs) from individuals harboring pathogenic tau mutations can reveal the earliest downstream effects on molecular pathways within a developmental context, generating interacting neurons and glia. We found that in hCOs carrying the V337M and R406W tau mutations, the cholesterol biosynthesis pathway in astrocytes was the top upregulated gene set compared with isogenic controls by single-cell RNA sequencing (scRNA-seq). The 15 upregulated genes included *HMGCR*, *ACAT2*, *STARD4*, *LDLR*, and *SREBF2*. This result was confirmed in a homozygous R406W mutant cell line by immunostaining and sterol measurements. Cholesterol abundance in the brain is tightly regulated by efflux and cholesterol biosynthetic enzyme levels in astrocytes, and dysregulation can cause aberrant phosphorylation of tau. Our findings suggest that cholesterol dyshomeostasis is an early event in the etiology of neurodegeneration caused by tau mutations.

INTRODUCTION

Among individuals with frontotemporal dementia (FTD) due to tauopathies, those with tau mutations represent paradigmatic exemplars of this entire disease category because the mutations clearly position the tau gene as the underlying cause of the disease. With the implication of tau mutations as the clear initiator of the disease process, the current challenge is to track the downstream molecular pathways that ultimately lead to the complex pathological and clinical phenotypes collectively referred to as FTD. The preparation of human cerebral organoids (hCOs) from induced pluripotent stem cells (iPSCs) harvested from mutation carriers and their isogenic controls is a potentially informative approach to detect the effects of mutations on cell types over development and maturation. Numerous studies indicate the emergence of cell populations with at least some degree of maturation including synaptic structures and neural network activity (Sidhaye and Knoblich, 2021).

In support of this approach, hCOs with *APP* and *PSEN1* mutations, hCOs generated from Down syndrome iPSCs, and hCOs carrying the Alzheimer disease (AD) APOE4 risk allele show AD hallmarks, including amyloid-beta accumulation and tau hyperphosphorylation (Raja et al., 2016; Gonzalez et al., 2018; Lee et al., 2016). hCOs with the *MAPT* V337M mutation have been reported to have increased tau phosphorylation, glutamatergic dysfunction, and glutamatergic neuron loss (Bowles et al., 2021). Importantly, the effects of tau mutations appear to begin during development, an inference that molecular pathology is evident before tau inclusions appear (Hernandez et al., 2019; Jiang et al., 2018).

Nonneuronal cells are increasingly recognized as critical players in neurodegenerative disease, including tauopathies (De Strooper and Karran 2016). Astrocytes can affect disease onset and progression (Matias et al. 2019) but have been less characterized than neurons in neurodegeneration. In FTD, astrogliosis and astrocytic degeneration are observed even at early disease stages (Broe et al., 2004), suggesting that molecular changes in astrocytes are early events in the etiology of FTD.

Using a guided approach for hCO production (Yoon et al., 2019), we generated a diversity of cell types with a focus on the astrocyte population. We performed single-cell RNA sequencing (scRNA-seq) on a set of hCOs from human iPSC lines with tau mutations and their isogenic controls to search for dysregulation of molecular pathways. The cholesterol synthetic pathway emerged as dysregulated in astrocytes from hCOs with tau mutations. Astrocytes are the predominant cell type that produce cholesterol in the adult brain (Saher and Stumpf, 2015). We validated the findings with immunohistochemistry and lipidomics. Accompanying the astrocytic changes,

This is an open access article under the CC BY license (http://creativecommons.org/licenses/by/4.0/).

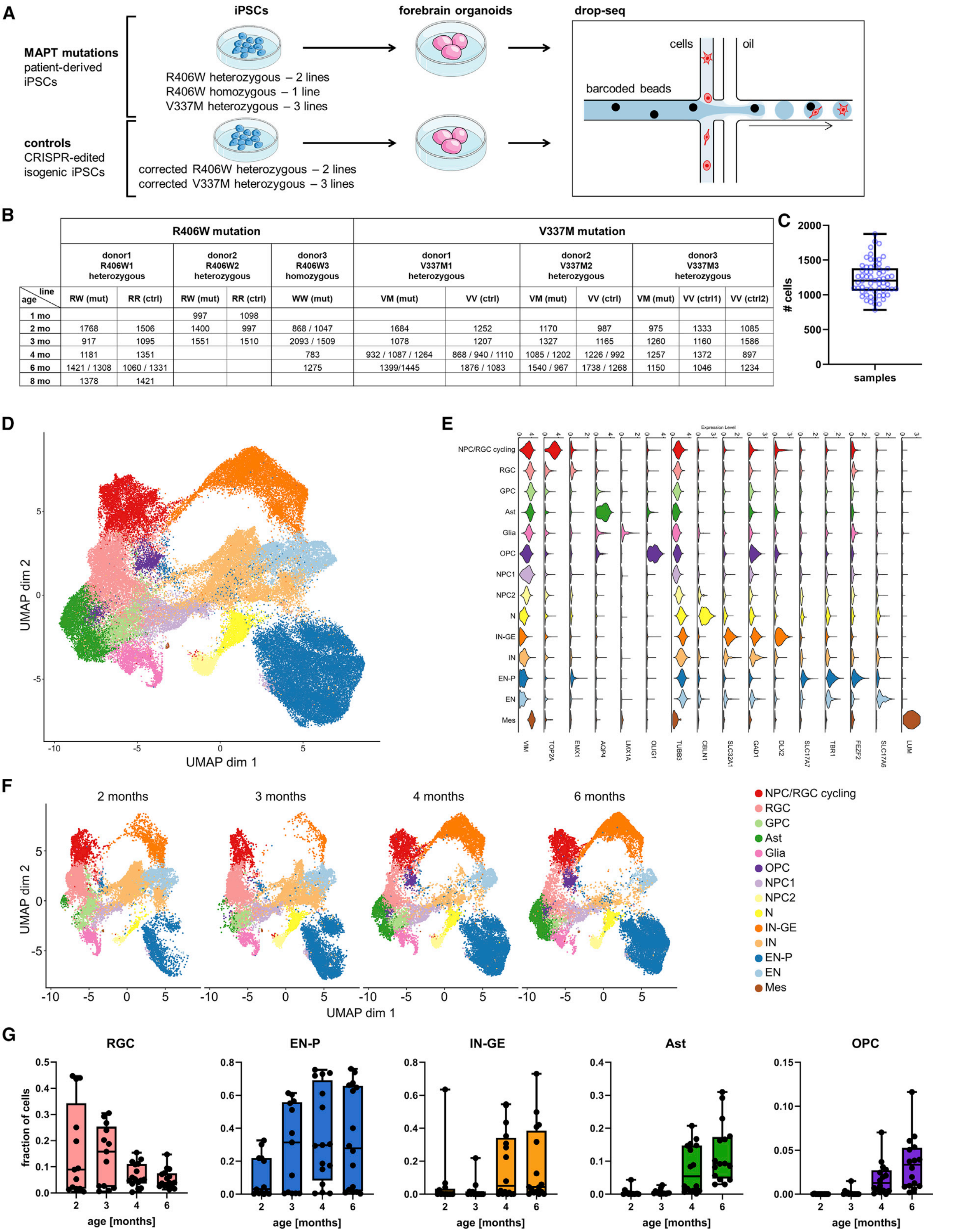

line / age	R406W mutation					V337M mutation						
	donor1 R406W1 heterozygous		donor2 R406W2 heterozygous		donor3 R406W3 homozygous	donor1 V337M1 heterozygous		donor2 V337M2 heterozygous		donor3 V337M3 heterozygous		
	RW (mut)	RR (ctrl)	RW (mut)	RR (ctrl)	WW (mut)	VM (mut)	VV (ctrl)	VM (mut)	VV (ctrl)	VM (mut)	VV (ctrl1)	VV (ctrl2)
1 mo			997	1098								
2 mo	1768	1506	1400	997	868 / 1047	1684	1252	1170	987	975	1333	1085
3 mo	917	1095	1551	1510	2093 / 1509	1078	1207	1327	1165	1260	1160	1586
4 mo	1181	1351			783	932 / 1087 / 1264	868 / 940 / 1110	1085 / 1202	1226 / 992	1257	1372	897
6 mo	1421 / 1308	1060 / 1331			1275	1399/1445	1876 / 1083	1540 / 967	1738 / 1268	1150	1046	1234
8 mo	1378	1421										

(legend on next page)

we observed decreased expression of glycolytic and GABA receptor genes in pyramidal neurons, showing that both neurons and astrocytes are affected by *MAPT* mutations. As cholesterol metabolism regulates aberrant tau phosphorylation (van der Kant et al., 2019), our study reveals astrocytic cholesterol biosynthesis as a disease-relevant pathway affected early on in the etiology of neurodegeneration.

RESULTS

Cellular diversity of hCOs derived from *MAPT* mutant carriers and isogenic controls

Tau pathology in FTD and AD massively affects the cerebral cortex (Arendt et al., 2016). To model the effect of pathogenic *MAPT* mutations in the human cerebral cortex, we relied on hCOs generated by directed differentiation (Yoon et al., 2019). iPSCs from two heterozygous *MAPT* R406W carriers, one homozygous R406W carrier and three heterozygous V337M, and CRISPR-corrected isogenic control for each heterozygous line were used for hCO production (Figures 1A and 1B; see experimental procedures for full line names and abbreviations). We used drop-seq (Macosko et al., 2015) to obtain sc transcriptomes for a total of 62 hCO samples, each consisting of three to four pooled hCOs, and 76,111 total cells post-filtering, with a mean of 1,228 cells ± 239.6 SD per sample (Figures 1B and 1C).

Clustering and uniform manifold approximation and projection (UMAP) of the dataset resulted in 14 major cell populations (Figure 1D; see experimental procedures). Marker genes for each cell population were computed (Table S1), manually probed for cell-type-specific genes found in the literature and in the PanglaoDB database (Franzén et al., 2019) (Figures 1E and S1A), and compared with published sc datasets (Giandomenico et al., 2019; Polioudakis et al., 2019; Quadrato et al., 2017; Velmeshev et al., 2019). The manually annotated identities were further confirmed by Gene Ontology (GO) enrichment analysis of the marker gene lists (Table S2).

The following neuronal populations were identified (Figures 1D and 1E): excitatory neurons expressing pyramidal cell (EN-P) markers (expressing, e.g., *BCL11B*, *SOX5*, *TBR1*, *SATB2*, *FEZF2*, *SLCC17A7*); GABAergic neurons expressing markers of the ventral forebrain and, more specifically, the lateral ganglionic eminences and dorsal portion of the caudal ganglionic eminences according to Long et al. (2009) (IN-GEs; expressing, e.g., *GAD1*, *GAD2*, *DLX1,2,5,6*, *ETV1*, *PBX3*, *ARX*, *SP9*); largely unidentified excitatory neurons (ENs; expressing, e.g., *MAPT*, *SLC17A6*, *SCN2A*, *ANKD3*); largely unidentified GABAergic neurons (INs; expressing, e.g., *GAD1*, *GAD2*); and other unidentified neurons (Ns; expressing, e.g., synaptic genes *SYT1*, *CPLX2*, *SYNJ2*). Two populations were identified as presumed neuronal progenitors based on low levels of both glial and neuronally expressed genes (neural progenitor cell [NPC1] and NPC2). Furthermore, we identified glial populations: radial glia (radial glia cells [RGCs]; expressing, e.g., *VIM*, *SOX2*, *PAX6*, *EMX2*); actively proliferating cells (RGC/NPC cycling; expressing, e.g., *VIM*, *EMX2*, *DLX1*, *TOP2A*, *CENPF*); largely unidentified glia expressing markers of choroid plexus, ependymal cells, and signaling molecules secreted by cortical hem and rhombic lip (glia; e.g., *TTR*, *HTR2C*, *TM4SF1*, *EFNB3*, *WNT2B*, *BMP7*); oligodendrocyte progenitors (OPCs; expressing, e.g., *OLIG1*, *OLIG2*, *PDGFRA*); astrocytes (Asts; expressing, e.g., *SLC1A2*, *GFAP*, *S100B*, *AQP4*); and a presumptive glial progenitor population (glial progenitor cells [GPCs]) expressing astrocyte markers (*SLC1A3*, *GFAP*, *S100B*) at levels intermediate

Figure 1. Composition of the human cerebral organoid (hCO) single-cell RNA dataset

(A) iPSCs from 6 donors with *MAPT* mutations and 5 isogenic controls were grown into hCOs and subjected to drop-seq.

(B) Overview of single-cell (sc) dataset and numbers of cells sequenced. Entries with more than one number indicate replicates from independent differentiation experiments, each number representing the number of cells sequenced for a replicate. For full line names, see supplemental experimental procedures.

(C) Cell numbers sequenced per sample (1,228 cells, mean ± 239.6 SD). Box and whiskers plots represent median (line in box center), first and third quartile (lower and upper box border, respectively), and minimum and maximum values (whiskers).

(D) UMAP of entire dataset, 62 samples and 76,111 cells. Colors represent cell types.

(E) Expression of selected canonical markers.

(F) UMAP subsetted to ages in months.

(G) Relative abundances (n cells in a cluster divided by total n of cells in a sample) of cell populations over hCO development. Box and whiskers plots represent median (line in box center), first and third quartile (lower and upper box border, respectively), and minimum and maximum values (whiskers).

Ast, astrocytes; Glia, glia expressing markers of choroid plexus, ependymal cells and Wnt/Bmp signaling molecules; EN, excitatory neurons; EN-P, pyramidal neurons; GPC, glial progenitor cells; IN, inhibitory neurons; IN-GE, inhibitory neurons derived from ganglionic eminences; Mes, mesenchymal cells; N, neurons; NPC1, neuronal progenitors 1; NPC2, neuronal progenitors 2; NPC/RGC-cycling, cycling neural progenitors and radial glia; RGC, radial glia; OPC, oligodendrocyte progenitors.

See also Figure S1.

between RGCs and Asts. A very small cluster of putative mesenchymal cells (Mess), similar to a population described in Camp et al. (2015), was identified (expressing, e.g., *LUM*, *DCN*, and multiple genes encoding collagens). Although the proportion of inhibitory neurons was larger than that reported in Yoon et al. (2019), the EN-P population was the predominant cell type in our dataset (Figure 1D).

The abundances of EN-Ps, IN-GEs, OPCs, and Asts increased over time whereas NPCs/RGCs decreased (Figures 1F, 1G, and S1C), as expected during hCO maturation (Lancaster et al., 2013; Paşca et al., 2015; Quadrato et al., 2017; Renner et al., 2017; Sloan et al., 2017) . However, a small minority of hCO preparations did not show abundant EN-Ps expected in hCOs (Figure S1B; supplemental experimental procedures) but either a high abundance of IN-GEs or unidentified neurons (Ns, INs, ENs) (Figure S1B). Such preparations were excluded from the analysis of astrocytes as described in the results. We did not detect significant effects of the *MAPT* mutations on the relative abundances of the 14 major cell populations (Figure S2D). However, given the variation in cellular composition (Figures S1B–S1D), it seems unlikely that we would be able to detect potentially subtle effects of *MAPT* mutations on cell type abundances.

Downregulation of the glycolytic pathway and GABA receptor genes in pyramidal neurons

Having identified the major cell types, we focused on effects of *MAPT* mutations on the population corresponding to pyramidal neurons (EN-Ps), as they are heavily impaired in neurodegenerative diseases (Fu et al., 2018). The EN-P cluster consists of distinctive subgroups (Figures 2A, 2B, S2A, and S2C): an intermediate progenitor and immature neuron cluster, two clusters enriched in deep- and upper-layer neurons, respectively, and a cluster characterized by high expression of mitochondrially encoded genes indicating cell damage or high metabolic demand (Figures 2A, 2B, and S2C). No statistically significant enrichment of mutant cells in any subcluster was found (Figures S2B and S2D).

Differential gene expression (DGE) in the clusters containing upper- and deep-layer-enriched clusters combined (“mature neurons”; Figure 2A) in ten mutant-isogenic pairs (from 5 hCO batches) with 4,044 cells yielded 60 downregulated genes (Figure 2C; Table S3), associated with the two top enriched GO terms (“NADH regeneration” and “cellular response to hypoxia”) (Figure 2D), both containing multiple enzymes in glycolysis (*ALDOA*, *ALDOC*, *ENO1*, *ENO2*, *HK1*, *PFKP*, *PGK1*) and *LDHA*, converting pyruvate into lactate (Figures 2C and 2E). Expression levels of these genes were decreased in the *MAPT* mutant across hCO ages (Figure 2E) and across isogenic pairs (Figure S3A). Decreased glucose metabolism has been reported from AD brains preceding memory deficits and during normal aging, while an increase in glycolysis can be neuroprotective (Goyal et al., 2017; Nordberg et al., 2010; Tang, 2020). The top GO term of the 81 upregulated genes (“negative regulation of execution phase of apoptosis”) (Figures 2C and 2D) predominantly included homologs of the mitochondrial *MT-RNR2* gene (*MTRNR2L* genes). Although their functions are poorly understood, upregulation of *MTRNR2L* genes has also been reported in a sc sequencing study of AD brains (Mathys et al., 2019). Glycolysis-related genes were also downregulated in mutant cells when analyzing upper- and deep-layer clusters separately (Figures S3B and S3C; Table S3), and the glycolytic genes identified in the analysis of mature neurons (Figures 2C and 2D) showed negative changes in both deep- and upper-layer clusters (Figure 2F).

Downregulated genes in upper-layer neurons were enriched in “inhibitory synapse assembly,” including *GABRA2*, *GABRG2*, and *NPAS4*, (Figures 2G and S3C), consistent with downregulation of GABA receptor genes in *MAPT* R406W iPSC-derived neurons and brains of *MAPT* R406W carriers (Jiang et al., 2018). A subset of GABA receptor and glycolytic genes was also downregulated in the homozygous R406W line compared with controls (Figure S3D; Table S3). These results show that *MAPT* mutations induce transcriptional changes in the glycolysis pathway and GABA receptors, both of which have been shown to be impacted in neurodegeneration. We next investigated how astrocytes participate in this disease environment set by *MAPT* mutations.

The cholesterol biosynthesis pathway is upregulated in *MAPT* mutant astrocytes

A subset of the hCO preparations showed unexpected cellular composition. We did not include these samples, but restricted our analysis to isogenic pairs that had >5% EN-Ps and <50% unidentified neurons (INs, ENs, Ns) in each sample, since the neuronal environment and regional identity affect astrocytes’ transcriptional profiles (Morel et al., 2017; Clarke et al., 2021). Five of the 22 samples containing sufficient astrocytes for analysis (supplemental experimental procedures) did not meet these criteria, resulting in the removal of five isogenic pairs from astrocyte analyses (Figure S1B). No statistically significant enrichment of *MAPT* mutant cells was found among astrocyte subclusters (Figures S4A–S4C), suggesting no population expansion or constriction effects of *MAPT* mutations. We next performed DGE analysis of astrocytes from hCO ages 4, 6, and 8 months combined, identifying 112 up- and 142 downregulated genes (Figure 3A; Table S3). “Cholesterol biosynthesis” was the top enriched GO term for upregulated genes in *MAPT* mutant astrocytes

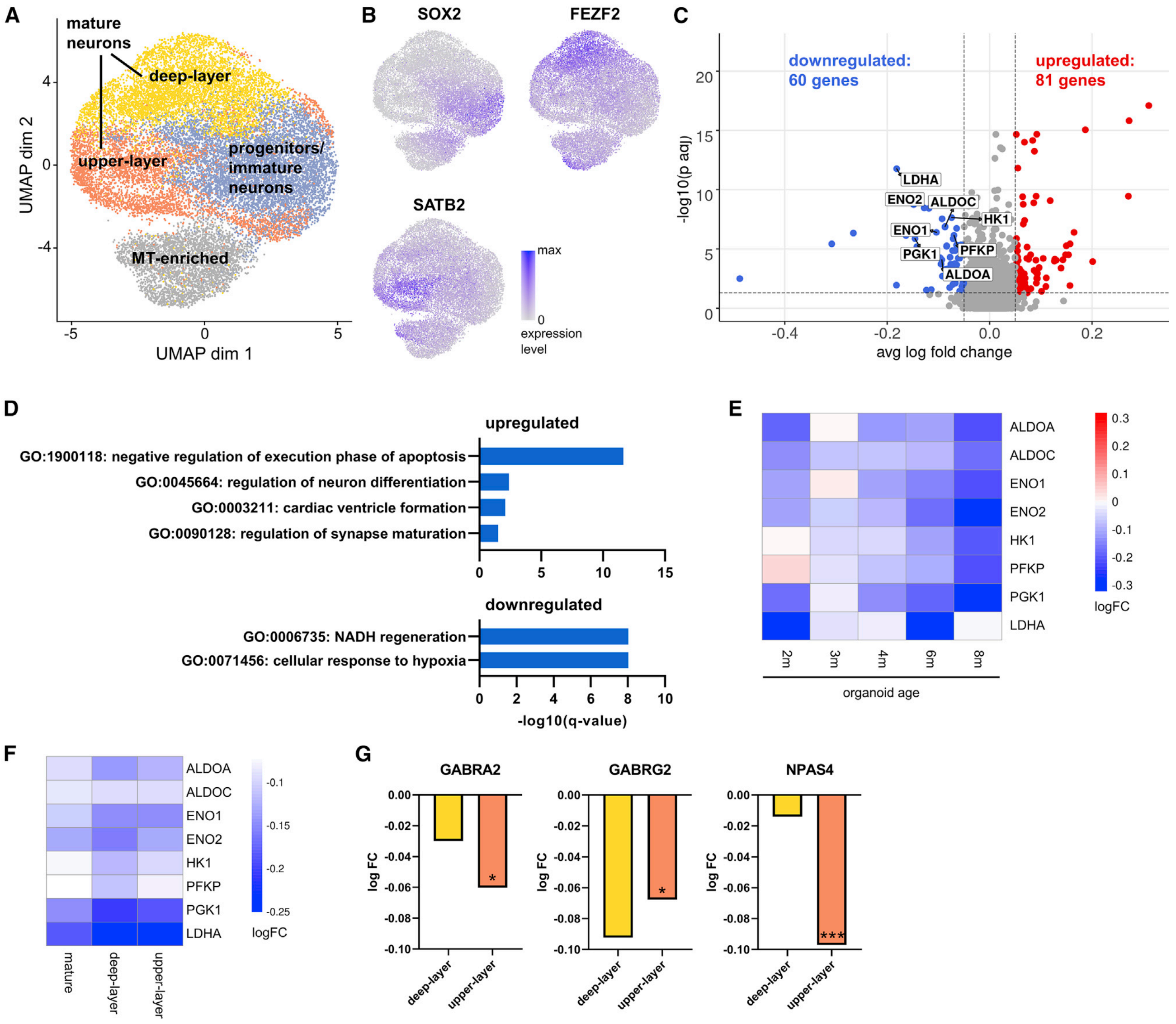

Figure 2. Effects of *MAPT* mutations on pyramidal glutamatergic neurons
(A) UMAP and subclustering of EN-Ps.
(B) Expression of selected subcluster markers.
(C) Volcano plot of differential gene expression (DGE) results of *MAPT* mutant versus control mature neurons, combining isogenic pairs with $\geq$30 mature neurons in each sample (n = 10 isogenic pairs from 5 hCO batches, 4,044 cells). Differentially expressed genes (DEGs) were identified using the MAST test (Finak et al., 2015) with a logFC cutoff of 0.05 and Benjamini-Hochberg (BH)-corrected p values <0.05. DEGs involved in glycolysis are highlighted.
(D) Gene Ontology (GO) enrichment of genes up- (top) and downregulated (bottom) in *MAPT* mutant mature EN-Ps.
(E) Heatmap of logFC of glycolysis-related genes identified in (C) across different ages.
(F) Heatmap of logFC of glycolysis-related genes identified in (C), and logFC in deep- and upper-layer neurons.
(G) Downregulation of GABA receptor genes *GABRA2* and *GABRG2*, and the immediate-early gene *NPAS4* in upper-layer neurons. Negative logFC indicates decreased gene expression in heterozygous mutant compared with control, positive logFC the opposite. $^{*}p \leq 0.05$, $^{***}p \leq 0.001$. See also Figures S2 and S3.

(Figure 3B). Fifteen of these genes encode enzymes of the cholesterol synthesis pathway, including its rate-limiting enzyme HMGCR (Figure 3A). Also, *ACAT2*, encoding an enzyme converting cholesterol to its storage form cholesteryl esters, *STARD4*, encoding an intracellular cholesterol transporter, *LDLR*, encoding an important receptor for

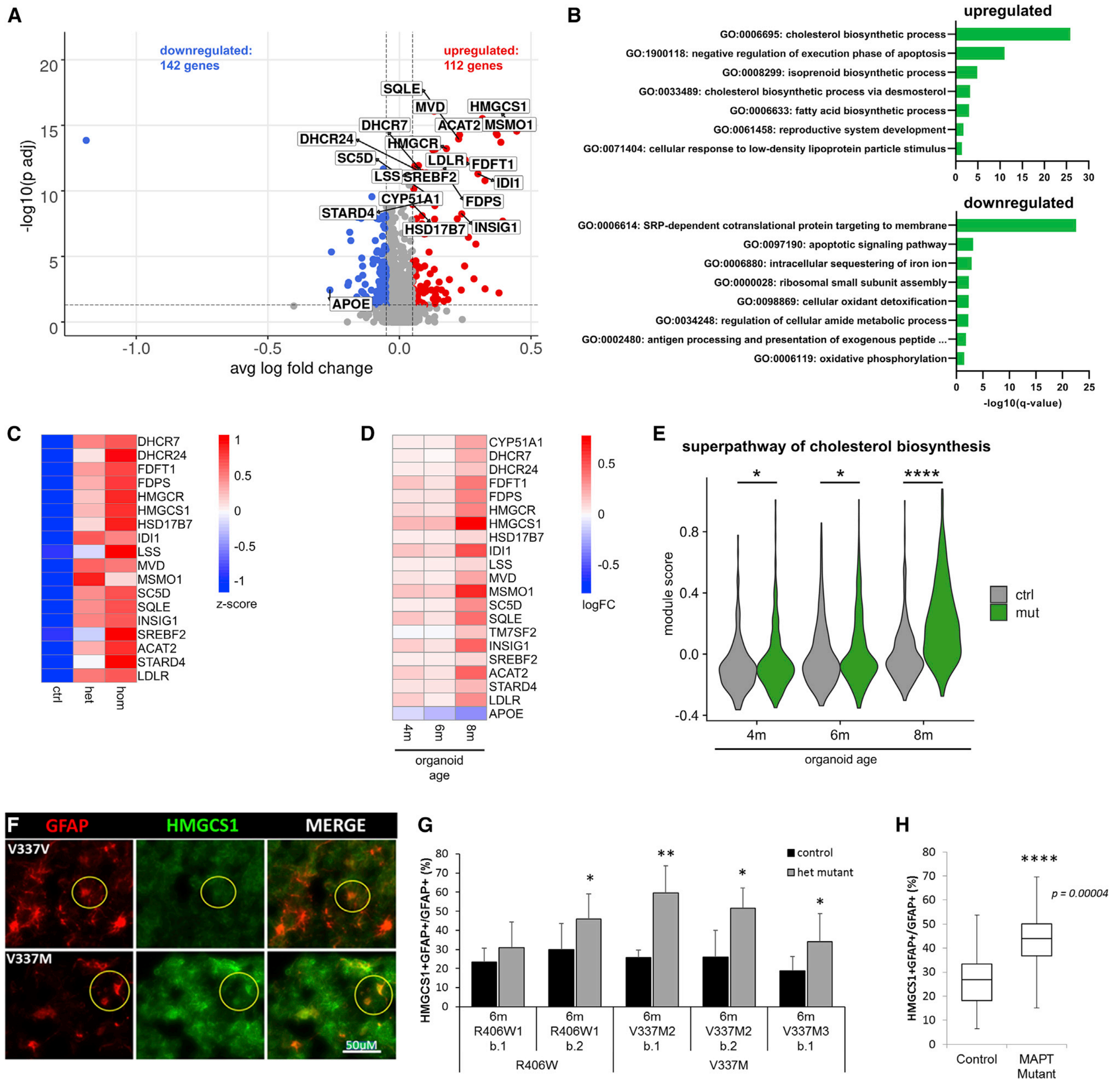

Figure 3. Effects of *MAPT* mutations on astrocytes

(A) Volcano plot of DGE in astrocytes of isogenic pairs with >5% EN-Ps and <50% INs, ENs, and Ns in each sample (n = 6 isogenic pairs from 4 hCO batches, 1,802 cells) using the MAST test with a logFC cutoff of 0.05 and BH-corrected p values <0.05. Cholesterol-related DEGs are highlighted.

(B) GO enrichment of genes up- (top) and downregulated (bottom) in *MAPT* mutant astrocytes including isogenic pairs with at least 30 mature astrocytes in each sample (n = 11 isogenic pairs from 7 hCO batches, 3,398 cells).

(C) Expression levels (*Z* scores) of cholesterol-related genes in astrocytes from control, heterozygous R406W and V337M mutant, homozygous R406W mutant.

(D) Heatmap of logFC of cholesterol-related genes identified in (A) across hCO ages. Positive logFC indicates increased gene expression in mutant (heterozygous and homozygous combined) compared with control, negative logFC the opposite.

(E) Violin plots showing gene signature enrichment scores of a cholesterol biosynthetic gene set (“superpathway of cholesterol biosynthesis” deposited in the Human Cyc database, 25 genes) across ages (4 months: n = 247 control cells, 254 mutant cells; 6 months, n = 286 control cells, 424 mutant cells; 8 months, n = 375 control cells, 375 mutant cells) and genotypes (mut, homozygous and heterozygous

(legend continued on next page)

cholesterol uptake, and *SREBF2*, encoding a transcriptional activator of cholesterol biosynthesis enzymes, were significantly upregulated (Figure 3A).

The increased expression of cholesterol biosynthesis genes was further confirmed in hCOs from the R406W homozygous line compared with control samples (Figure S4D; Table S3). GO enrichment analysis of the upregulated genes again revealed the cholesterol biosynthesis pathway as a top category (Figure S4E). Eighteen out of the 21 cholesterol-related genes identified as differentially expressed (DE) in the heterozygous mutants were also DE in the homozygous R406W mutant (Figure 3C).

We next identified age effects across 4–8 months (i.e., stages when astrocytes are present), revealing positive log fold changes (FC) of the previously identified DE cholesterol-related genes (Figure 3A) across ages (heterozygous and homozygous combined; Figure 3D). Furthermore, examination of a cholesterol biosynthesis signature ("superpathway of cholesterol biosynthesis," consisting of 25 genes; Caspi et al., 2018) in genotypes and across ages revealed significant increases in the expression of this signature in mutant (heterozygous and homozygous combined) versus control astrocytes at 4, 6, and 8 months of age, with the largest size effect and significance level at 8 months (Figure 3E).

The transcription factor SREBF2 activates cholesterol biosynthesis genes and genes acting in long-chain fatty acid synthesis (Madison 2016). GO terms related to fatty acid synthesis were enriched in the genes upregulated in *MAPT* mutant astrocytes (Figure 3B), including the fatty acid synthase gene *FASN* (Figure S4F), modulating neurodegeneration-related toxicities (Ates et al., 2020). All eight upregulated genes included in the GO term "fatty acid biosynthetic process" were also upregulated in the homozygous R406W mutant line (Figure S5F).

Concomitantly, we identified downregulation of *APOE* in *MAPT* mutant astrocytes (Figure 3A). *APOE* encodes an apolipoprotein important for brain cholesterol transport and is a prominent risk gene for AD (Corder et al., 1993). Genes involved in cholesterol synthesis and the *APOE* gene show the same directionalities of change in lines with APOE3/3 (2 lines), APOE2/3 (1 line), and APOE4/4 (1 line) genotypes (Figure S4G; supplemental experimental procedures), indicating that these changes may occur irrespective of the APOE variant.

To rule out the possibility that our filtering had artificially produced the finding of upregulated cholesterol biosynthetic enzymes, we performed DGE testing on all isogenic pairs (Figure S4H; Table S3). This approach still resulted in the cholesterol biosynthesis pathway as upregulated according to GO enrichment terms (Figure S4I) and upregulated *HMGCR* (Figure S4H). However, the directionality of change of the previously identified cholesterol-related DE genes (Figure 3A) was more consistently positive in the isogenic pairs that did meet the criteria stated above (Figure S4G). No population expansion or restriction effects were present in either approach (Figures S4C and S4J). *MAPT* was expressed in mutant and control astrocytes, albeit at lower levels than in neurons, (Figure S4K), opening up the possibility of cell-autonomous effects of *MAPT* mutations on astrocytes.

We validated upregulation of a cholesterol biosynthetic enzyme with immunohistochemistry: co-labeling of HMGCS1, encoded by the cholesterol-related gene with the highest logFC in our analysis, and the astrocyte marker GFAP revealed a higher proportion of HMGCS1-positive astrocytes in mutant hCOs compared with control.

Age-dependent elevation of cholesterol and its precursors in *MAPT* mutant organoids

To investigate whether the observed changes in the cholesterol biosynthesis pathway were accompanied by altered levels of cholesterol and its biosynthesis intermediates, we utilized liquid chromatography-mass spectrometry (LC-MS)-based lipidomics. hCOs from one R406W heterozygous line (R406W1) and two V337M heterozygous lines (V337M2, V337M3) and their respective isogenic control were analyzed for sterols at ages 4 and 7 months. We analyzed five hCOs per age group and cell line and therefore a total of 60 individual hCOs (Figure 4A). The sterol panel included cholesterol and 11 of its precursors (Figure S5A). For statistical analysis, we included metabolites that were detected in at least 50% of the hCO samples in each of the age groups (Figures 4B, 4C, S5B, and S5C), resulting in a set of cholesterol and 7 of its precursors (Figure 4B), the cholesterol derivative cholestanol (Figure 4C),

combined; ctrl, control cells). Statistical significance was determined using two-sided Wilcoxon rank-sum test and BH-corrected p values for 3 comparisons.

(F) Representative image of HMGCS1 and GFAP co-labeling in 6-month-old V337M mutant hCOs and isogenic control.

(G) Percentages of HMGCS1-positive astrocytes (GFAP+) from two R406W and three V337M isogenic pairs across three hCO prep dates at 6 months. Bars and error bars represent means ± SD.

(H) Percentages of HMGCS1+ astrocytes (GFAP+) from all three isogenic *MAPT* mutant and control lines. Box and whiskers plots represent median (line in box center), first and third quartile (lower and upper box border, respectively), and minimum and maximum values (whiskers). Student's t test was used to determine significance. $^{*}p \leq 0.1$, $^{**}p \leq 0.01$, $^{****}p \leq 0.0001$.

See also Figure S4.

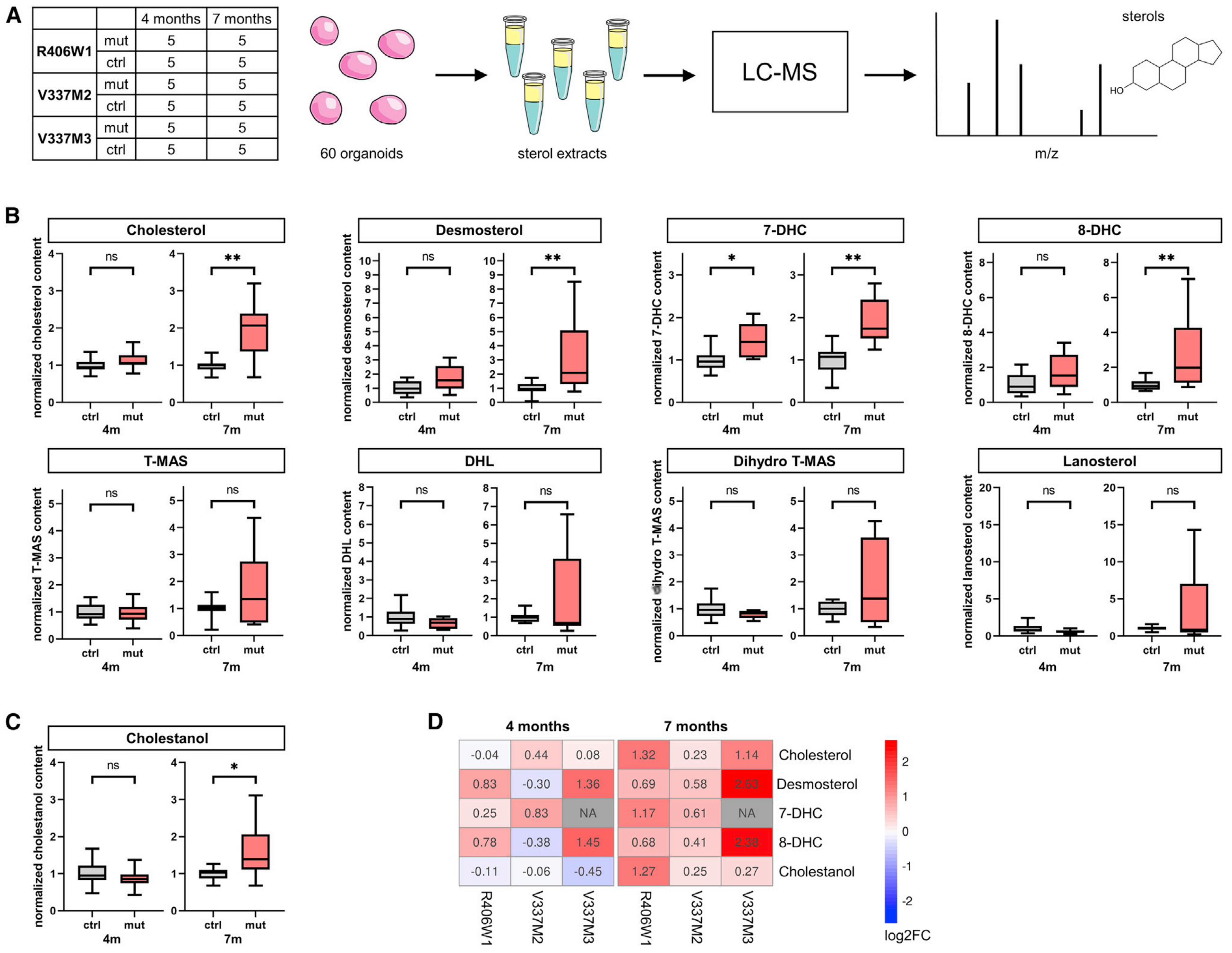

Figure 4. Sterol quantification in *MAPT* mutant hCOs

(A) Mutant and control hCOs from three isogenic lines were subjected to sterol analysis with liquid chromatography-mass spectrometry (LC-MS). Five individual hCOs from each line were analyzed at 4 and 7 months hCO age.

(B and C) LC-MS data of all three isogenic lines combined. Metabolites detected in >50% hCO samples at each time point were analyzed. n = 15 from 3 hCO batches for each control and mutant at each time point except for 7-DHC (n = 10 from 2 hCO batches for each group at each time point), T-MAS (n = 10 from 2 hCO batches for each group at each time point), and Dihydro T-MAS (4 months control n = 14 from 3 hCO batches, 4 months mutant n = 15 from 3 hCO batches, 7 months n = 10 from 2 hCO batches for each group); see also Figure S5C. Mutant values were normalized to control. Box and whiskers plots represent median (line in box center), first and third quartile (lower and upper box border, respectively), and minimum and maximum values (whiskers). Significance was determined using Wilcoxon rank-sum test. BH-corrected p values (for 22 multiple comparisons) <0.05 are reported as statistically significant. *$p \leq 0.05$, **$p \leq 0.01$.

(D) Heatmap of log2FC for the three isogenic pairs. Positive log2FC indicates increased concentrations in mutant hCOs compared with control, negative log2FC the opposite.

See also Figure S5.

and two phytosterols (Figure S5A). Other cholesterol biosynthesis intermediates were not detected in most samples, likely due to small size of hCOs and therefore low sample input.

A combined analysis of all hCOs within each time point revealed a significant increase of cholesterol, its precursors desmosterol, 7-dehydrocholesterol (7-DHC), 8-DHC, and the cholesterol derivative cholestanol at 7 months (Figures 4B and 4C). Cholestanol accumulates in cerebrotendinous xanthomatosis, often involving neurological symptoms (Björkhem 2013). Each isogenic pair (R406W1, V337M2, V337M3) consistently showed increased cholesterol, desmosterol, 7-DHC, 8-DHC, and cholestanol at 7 months (Figures 4D and S5B). At 4 months, only

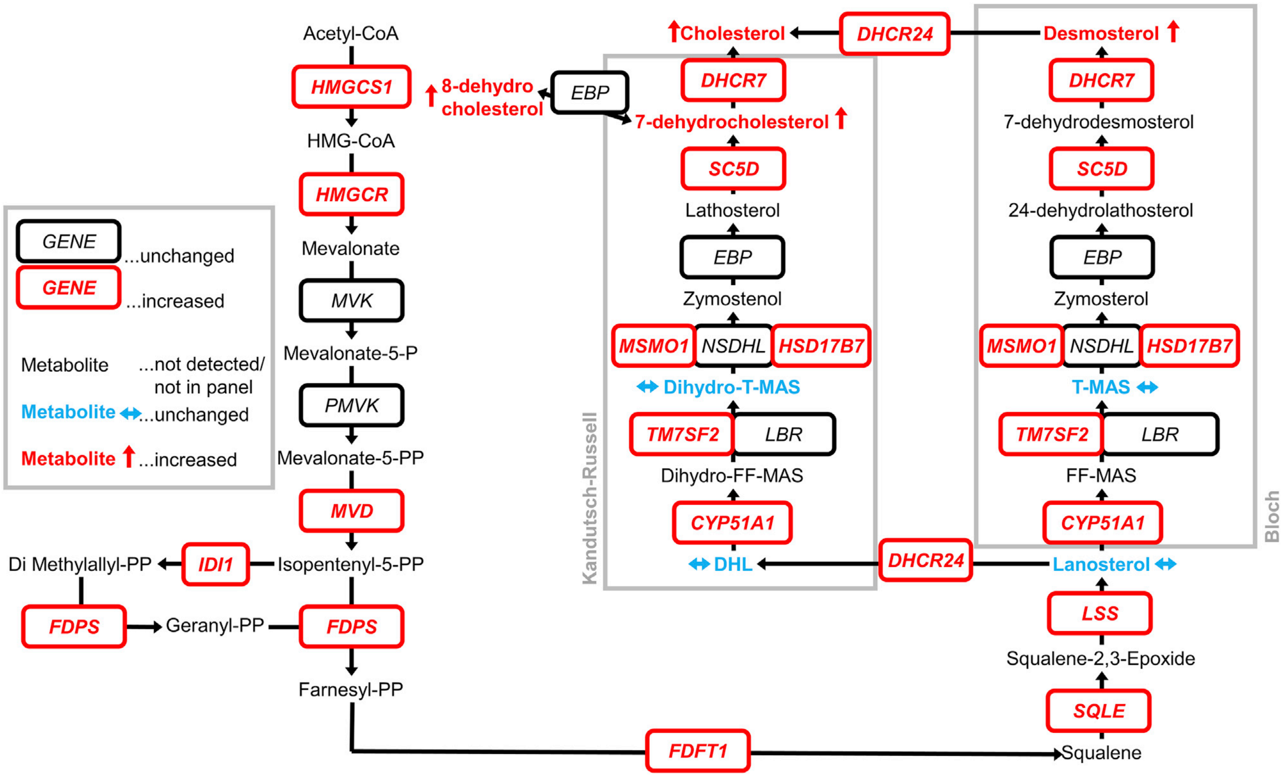

Figure 5. Summary of changes in cholesterol biosynthesis caused by *MAPT* mutations

Boxed/italic, genes encoding cholesterol biosynthetic enzymes; red/boxed/italic, genes with increased expression in *MAPT* mutant astrocytes, determined by scRNA-seq; black/boxed/italic, genes with unaltered expression; unboxed, metabolites (cholesterol and its precursors); red/arrow up, metabolites with increased levels in *MAPT* mutant hCOs determined by LC-MS; blue/arrow sideways, metabolites with unaltered levels; black, metabolites not detected or not included in the sterol panel.

7-DHC was significantly increased (Figure 4B). Levels of precursors generated in earlier steps of cholesterol synthesis (T-MAS, DHL, Dihydro T-MAS, lanosterol) were not altered at either time point. This pattern may be explained by elevated cholesterol levels resulting in product inhibition (Cornish-Bowden, 2013) of the enzymes catalyzing the last steps of cholesterol biosynthesis.

Summarized, lipidomics corroborate our transcriptomic and immunohistochemical findings of dysregulated cholesterol synthesis (Figure 5), and the increase of metabolites lags behind the increase in the relevant mRNAs.

DISCUSSION

The difficulty in distinguishing causes from consequences when evaluating tau pathology can be addressed by tracking the effects of pathological tau mutations from early in development across the variety of affected cells. Hence, the utility of hCOs. Our article links mutations of the *MAPT* gene to lipid dysregulation and therefore posits that the vector of the disease diathesis projects from tau mutations to dysregulation of the cholesterol pathway. We found increased expression of cholesterol biosynthetic genes in astrocytes and validated these results with lipidomics. Whether the effects of the *MAPT* mutations on astrocytes are indirect or cell autonomous remains open. On one hand, *MAPT* is highly expressed in neurons, making it plausible that the astrocytic change in cholesterol synthesis is a response to impaired neuronal function induced by mutant tau. On the other hand, we detected *MAPT* mRNA in astrocytes, in line with reports of tau protein or mRNA in astrocytes (Kovacs 2020), raising the possibility of cell-autonomous effects, as reported from N279K *MAPT* mutant astrocytes (Hallmann et al., 2017). As we did not observe altered numbers of astrocytes, neurons, or precursors in *MAPT* mutant hCOs (Figure S1D), it seems likely that upregulation of cholesterol biosynthesis in *MAPT* mutant astrocytes is a specific effect and not the by-product of a more general effect such as impaired maturation.

A growing body of evidence links dysregulation of lipid metabolism to AD (Arenas et al., 2017). The most direct

link between cholesterol and neurodegeneration is impairment in lysosomal cholesterol transport causing Niemann-Pick Type C disease, characterized by accumulation of cholesterol, other lipids, and tau tangles (Auer et al., 1995; Suzuki et al., 1995). A recent report linked FTD and cholesterol metabolism by showing that plasma cholesterol is increased in FTD (Wang et al., 2020).

Our findings may also be relevant to the emerging role of hyperexcitability in tauopathies, an effect that has also been observed in *MAPT* V337M mutant iPSC-derived neurons (Sohn et al., 2019). As cholesterol can alter membrane properties and modulate synaptic signaling (Korinek et al., 2015, 2020), it is tempting to speculate that elevated cholesterol early on in disease etiology may contribute to hyperexcitability in FTD. Sterol measurements also revealed elevation of cholesterol precursors in *MAPT* mutant hCOs. It is conceivable that these precursors themselves or products derived from them contribute to pathogenesis.

Another top enriched category in genes upregulated in astrocytes of *MAPT* mutant hCOs was fatty acid biosynthesis. Several free polyunsaturated fatty acids, including arachidonic acid, increase tau polymerization (Wilson and Binder 1997; King et al., 2000). Interestingly, the upregulated genes encode three fatty acid desaturases: FADS1, FADS2, and SCD. FADS1 and FADS2 rate limit in fatty acid desaturation and catalyze conversion of linoleic to arachidonic acid. Recently, a genetic *FADS1* variant was shown to increase the levels of arachidonic acid and AD risk (Hammouda et al., 2020). Two other upregulated genes encode enzymes in long-chain saturated fatty acid synthesis: ELOVL6 and FASN. This is intriguing, as long-chain saturated fatty acids are secreted by neurotoxic astrocytes and induce cell death (Guttenplan et al., 2021). ELOVL6 alters the expression of endoplasmic reticulum stress genes (Matsuzaka et al., 2007), known to play a central role in neurodegeneration (Hetz and Saxena 2017).

Astrocytes of *MAPT* mutant hCOs did not show the inflammatory signature of astrocyte activation (Zamanian et al., 2012; Liddelow et al., 2017) or disease-associated astrocytes (Habib et al., 2020). However, *IFITM3*, the only upregulated gene with direct function in the immune response, disrupts cholesterol homeostasis (Amini-Bavil-Olyaee et al., 2013). Furthermore, increased astrocytic IFITM3 expression causes neuronal impairments (Ibi et al., 2013), making it a possibility that IFITM3 functionally connects astrocytic cholesterol dyshomeostasis and neuronal impairment in neurodegeneration.

An important line of evidence for a central role of cholesterol in neurodegeneration was the discovery of the APOE4 allele as the strongest genetic risk factor for late onset AD (Corder et al., 1993), with modulatory effects on tau pathology and tau-related neurodegeneration (Shi et al., 2017). We identified decreased *APOE* transcript levels in astrocytes of *MAPT* mutant hCOs. Expression of astrocytic *APOE* was also decreased in two sc studies of AD (Grubman et al., 2019; Mathys et al., 2019) and in iPSC-derived APOE4 astrocytes versus APOE3 (Lin et al., 2018). Interestingly, a recent study in cerebral hCOs has identified lipidomic changes as a result of *APOE* knockout (Zhao et al., 2021).

The mechanisms of how *MAPT* mutations lead to elevated cholesterol biosynthesis are yet to be determined. One interesting possibility is that the formation of tau oligomers from mutant tau might damage membranes (Flach et al., 2012), and increased cholesterol biosynthesis might be a compensatory response. According to a recent report (Tuck et al., 2022), such elevated membrane cholesterol might in turn protect neurons from tau spread, while cholesteryl esters, on the other hand, play a role in the formation of pathological tau (van der Kant et al., 2019), highlighting the multifaceted role of cholesterol in neurodegeneration. It is also conceivable that cholesterol biosynthesis is increased by toxic tau species via stress signaling.

In summary, our study identifies increased cholesterol biosynthesis downstream of tau mutations. Our findings suggest that astrocytic changes in cholesterol biosynthesis and transport occur in the absence of astrogliosis, severe loss of neurons, and tau inclusions, suggesting that elevated cholesterol biosynthesis precedes these events in the etiology of neurodegeneration caused by *MAPT* mutations. Perturbed cholesterol metabolism is emerging as a shared feature of neurodegenerative diseases, including AD, Parkinson disease, Niemann-Pick Type C, and FTD, and, given our findings, may be an early event in the disease process, warranting further mechanistic investigation with a goal toward early intervention.

EXPERIMENTAL PROCEDURES

iPSCs used in this study were established from the Tau Consortium iPSC line collection (Karch et al., 2019), grown at NSCI core facility NeuraCell, and are available upon request (www.neuralsci.org/tau). hCOs were generated using described protocols (Yoon et al., 2019; Gregory et al., 2020). For scRNA-seq, three to four hCOs were pooled for dissociation, and drop-seq was performed as described (Macosko et al., 2015). Libraries were sequenced on an Illumina Nextseq500 instrument at 50,000 reads per cell. Counts matrices were generated using the Drop-seq tools package (Macosko et al., 2015). Downstream analysis was performed using Seurat 3.0 (Butler et al., 2018; Stuart et al., 2019). The filtered dataset had means of 1,499 transcripts and 930 genes per cell. The mean content of mitochondrially encoded genes was 2.1%. Data were imputed using SAVER (Huang et al., 2018). Free sterol/oxysterol analysis (Quehenberger et al., 2010) of individual cerebral hCOs was performed using LC/MS. See supplemental experimental procedures for details.

Data and code availability

scRNA-seq data are available in NCBI's GEO under GEO: GSE208418 (https://www.ncbi.nlm.nih.gov/geo/query/acc.cgi?acc=GSE208418). The Seurat object and code used for data analysis were deposited on Dryad under https://doi.org/10.25349/D95898.

SUPPLEMENTAL INFORMATION

Supplemental information can be found online at https://doi.org/10.1016/j.stemcr.2022.07.011.

AUTHOR CONTRIBUTIONS

Conceptualization, S.M.K.G., K.S.K., and S.T.; investigation, S.M.K.G., S.K.G., J.N.R., M.A., E.R., G.L., E.K.-R., and A.M.A; methodology, S.M.K.G., S.K.G., J.N.R., S.L., S.B., A.M.A., and O.Q.; formal analysis, S.M.K.G., T.B., E.G., S.J., and A.M.A.; visualization, S.M.K.G. and T.B.; resources, O.Q., S.T., and K.S.K.; software, S.M.K.G. and E.G; supervision, O.Q., S.T., and K.S.K.; project administration, T.B., S.T., and K.S.K.; funding acquisition, S.M.K.G., J.N.R., S.T., and K.S.K.; writing – original draft, S.M.K.G. and K.S.K.; writing – review and editing, J.N.R., M.A., E.G., T.B., S.J., A.M.A., O.Q., and S.T.

ACKNOWLEDGMENTS

We thank Alexander Franks and Megan Elcheikhali for expert statistical advice and Nathan Boles for feedback on the manuscript. iPSCs and hCOs were cultured at NeuraCell, Neural Stem Cell Institute, NY, USA (www.http://neuralsci.org/neuracell) with coordination by Khadijah Onanuga. The authors acknowledge the use of the Biological Nanostructures Laboratory within the California NanoSystems Institute, supported by the University of California, Santa Barbara and the University of California, Office of the President, and the DNA Technologies and Expression Analysis Core at the UC Davis Genome Center, supported by NIH Shared Instrumentation Grant 1S10OD010786-01. Lipid analysis was performed at UCSD Lipidomics Core. S.M.K.G. was the recipient of Swiss National Science Foundation Early Postdoc.Mobility and Postdoc.Mobility grants (P2ZHP3_174753 and P400PB_186800). This study was funded by the Rainwater Charitable Foundation; NINDS U54 NS100717-01(K.S.K.); NIH K99 grant to J.N.R. (NIH K99: AG064116); Larry L Hillblom Foundation (K.S.K.); NINDS R35 NS097277 (S.T.); NIA (AG056293 to S.T.); and CurePSP (S.T.). The recruitment and clinical characterization of research participants at Washington University were supported by NIH P30 AG066444, P01 AG03991, and P01 AG026276.

CONFLICTS OF INTEREST

The authors declare no competing interests.

Received: June 15, 2022
Revised: July 18, 2022
Accepted: July 19, 2022
Published: August 18, 2022

REFERENCES

Amini-Bavil-Olyaee, S., Choi, Y.J., Lee, J.H., Shi, M., Huang, I.C., Farzan, M., and Jung, J.U. (2013). The antiviral effector IFITM3 disrupts intracellular cholesterol homeostasis to block viral entry. Cell Host Microbe *13*, 452–464. https://doi.org/10.1016/j.chom.2013.03.006.

Arenas, F., Garcia-Ruiz, C., and Fernandez-Checa, J.C. (2017). Intracellular cholesterol trafficking and impact in neurodegeneration. Front. Mol. Neurosci. *10*, 382. https://doi.org/10.3389/fnmol.2017.00382.

Arendt, T., Stieler, J.T., and Holzer, M. (2016). Tau and tauopathies. Brain Res. Bull. *126*, 238–292. https://doi.org/10.1016/j.brainresbull.2016.08.018.

Ates, G., Goldberg, J., Currais, A., and Maher, P. (2020). CMS121, a fatty acid synthase inhibitor, protects against excess lipid peroxidation and inflammation and alleviates cognitive loss in a transgenic mouse model of Alzheimer's disease. Redox Biol. *36*, 101648. https://doi.org/10.1016/j.redox.2020.101648.

Auer, I.A., Schmidt, M.L., Lee, V.M., Curry, B., Suzuki, K., Shin, R.W., Pentchev, P.G., Carstea, E.D., and Trojanowski, J.Q. (1995). Paired helical filament tau (PHFtau) in Niemann-Pick type C disease is similar to PHFtau in Alzheimer's disease. Acta Neuropathol. *90*, 547–551. https://doi.org/10.1007/BF00318566.

Björkhem, I. (2013). Cerebrotendinous xanthomatosis. Curr. Opin. Lipidol. *24*, 283–287. https://doi.org/10.1097/MOL.0b013e328362df13.

Bowles, K.R., Silva, M.C., Whitney, K., Bertucci, T., Berlind, J.E., Lai, J.D., Garza, J.C., Boles, N.C., Mahali, S., Strang, K.H., et al. (2021). ELAVL4, splicing, and glutamatergic dysfunction precede neuron loss in MAPT mutation cerebral organoids. Cell *184*, 4547–4563.e17. https://doi.org/10.1016/j.cell.2021.07.003.

Broe, M., Kril, J., and Halliday, G.M. (2004). Astrocytic degeneration relates to the severity of disease in frontotemporal dementia. Brain *127*, 2214–2220. https://doi.org/10.1093/brain/awh250.

Butler, A., Hoffman, P., Smibert, P., Papalexi, E., and Satija, R. (2018). Integrating single-cell transcriptomic data across different conditions, technologies, and species. Nat. Biotechnol. *36*, 411–420. https://doi.org/10.1038/nbt.4096.

Camp, J.G., Badsha, F., Florio, M., Kanton, S., Gerber, T., Wilsch-Bräuninger, M., Lewitus, E., Sykes, A., Hevers, W., Lancaster, M., et al. (2015). Human cerebral organoids recapitulate gene expression programs of fetal neocortex development. Proc. Natl. Acad. Sci. USA *112*, 15672–15677. https://doi.org/10.1073/pnas.1520760112.

Caspi, R., Billington, R., Fulcher, C.A., Keseler, I.M., Kothari, A., Krummenacker, M., Latendresse, M., Midford, P.E., Ong, Q., Ong, W.K., and Paley, S. (2018). The MetaCyc database of metabolic pathways and enzymes. Nucleic. Acids Res. *46*, D633–D639. https://doi.org/10.1093/nar/gkx935.

Clarke, B.E., Taha, D.M., Tyzack, G.E., and Patani, R. (2021). Regionally encoded functional heterogeneity of astrocytes in health and disease: a perspective. Glia *69*, 20–27. https://doi.org/10.1002/glia.23877.

Corder, E.H., Saunders, A.M., Strittmatter, W.J., Schmechel, D.E., Gaskell, P.C., Small, G.W., Roses, A.D., Haines, J.L., and

Pericak-Vance, M.A. (1993). Gene dose of apolipoprotein E type 4 allele and the risk of Alzheimer's disease in late onset families. Science *261*, 921–923. https://doi.org/10.1126/science.8346443.

Cornish-Bowden, A. (2013). Fundamentals of Enzyme Kinetics (John Wiley & Sons).

De Strooper, B., and Karran, E. (2016). The cellular phase of Alzheimer's disease. Cell *164*, 603–615. https://doi.org/10.1016/j.cell.2015.12.056.

Finak, G., McDavid, A., Yajima, M., Deng, J., Gersuk, V., Shalek, A.K., Slichter, C.K., Miller, H.W., McElrath, M.J., Prlic, M., et al. (2015). MAST: a flexible statistical framework for assessing transcriptional changes and characterizing heterogeneity in single-cell RNA sequencing data. Genome Biol. *16*, 278. https://doi.org/10.1186/s13059-015-0844-5.

Flach, K., Hilbrich, I., Schiffmann, A., Gärtner, U., Krüger, M., Leonhardt, M., Waschipky, H., Wick, L., Arendt, T., and Holzer, M. (2012). Tau oligomers impair artificial membrane integrity and cellular viability. J. Biol. Chem. *287*, 43223–43233. https://doi.org/10.1074/jbc.M112.396176.

Franzén, O., Gan, L.M., and Björkegren, J. (2019). PanglaoDB: a web server for exploration of mouse and human single-cell RNA sequencing data. Database, baz046. https://doi.org/10.1093/database/baz046.

Fu, H., Hardy, J., and Duff, K.E. (2018). Selective vulnerability in neurodegenerative diseases. Nat. Neurosci. *21*, 1350–1358. https://doi.org/10.1038/s41593-018-0221-2.

Giandomenico, S.L., Mierau, S.B., Gibbons, G.M., Wenger, L.M.D., Masullo, L., Sit, T., Sutcliffe, M., Boulanger, J., Tripodi, M., Derivery, E., et al. (2019). Cerebral organoids at the air-liquid interface generate diverse nerve tracts with functional output. Nat. Neurosci. *22*, 669–679. https://doi.org/10.1038/s41593-019-0350-2.

Gonzalez, C., Armijo, E., Bravo-Alegria, J., Becerra-Calixto, A., Mays, C.E., and Soto, C. (2018). Modeling amyloid beta and tau pathology in human cerebral organoids. Mol. Psychiatry *23*, 2363–2374. https://doi.org/10.1038/s41380-018-0229-8.

Goyal, M.S., Vlassenko, A.G., Blazey, T.M., Su, Y., Couture, L.E., Durbin, T.J., Bateman, R.J., Benzinger, T.L.S., Morris, J.C., and Raichle, M.E. (2017). Loss of brain aerobic glycolysis in normal human aging. Cell Metab. *26* (2), 353–360. https://doi.org/10.1016/j.cmet.2017.07.010.

Gregory, J.A., Hoelzli, E., Abdelaal, R., Braine, C., Cuevas, M., Halpern, M., Barretto, N., Schrode, N., Akbalik, G., Kang, K., et al. (2020). Cell type-specific in vitro gene expression profiling of stem cell-derived neural models. Cells *9*, 1406. https://doi.org/10.3390/cells9061406.

Grubman, A., Chew, G., Ouyang, J.F., Sun, G., Choo, X.Y., McLean, C., Simmons, R.K., Buckberry, S., Vargas-Landin, D.B., Poppe, D., et al. (2019). A single-cell atlas of entorhinal cortex from individuals with Alzheimer's disease reveals cell-type-specific gene expression regulation. Nat. Neurosci. *22*, 2087–2097. https://doi.org/10.1038/s41593-019-0539-4.

Guttenplan, K.A., Weigel, M.K., Prakash, P., Wijewardhane, P.R., Hasel, P., Rufen-Blanchette, U., Münch, A.E., Blum, J.A., Fine, J., Neal, M.C., et al. (2021). Neurotoxic reactive astrocytes induce cell death via saturated lipids. Nature *599*, 102–107. https://doi.org/10.1038/s41586-021-03960-y.

Habib, N., McCabe, C., Medina, S., Varshavsky, M., Kitsberg, D., Dvir-Szternfeld, R., Green, G., Dionne, D., Nguyen, L., Marshall, J.L., et al. (2020). Disease-associated astrocytes in Alzheimer's disease and aging. Nat. Neurosci. *23*, 701–706. https://doi.org/10.1038/s41593-020-0624-8.

Hallmann, A.L., Araúzo-Bravo, M.J., Mavrommatis, L., Ehrlich, M., Röpke, A., Brockhaus, J., Missler, M., Sterneckert, J., Schöler, H.R., Kuhlmann, T., et al. (2017). Astrocyte pathology in a human neural stem cell model of frontotemporal dementia caused by mutant TAU protein. Sci. Rep. *7*, 42991. https://doi.org/10.1038/srep42991.

Hammouda, S., Ghzaiel, I., Khamlaoui, W., Hammami, S., Mhenni, S.Y., Samet, S., Hammami, M., and Zarrouk, A. (2020). Genetic variants in FADS1 and ELOVL2 increase level of arachidonic acid and the risk of Alzhei'er's disease in the Tunisian population. Prostaglandins Leukot. Essent. Fatty Acids *160*, 102159. https://doi.org/10.1016/j.plefa.2020.102159.

Hernandez, I., Luna, G., Rauch, J.N., Reis, S.A., Giroux, M., Karch, C.M., Boctor, D., Sibih, Y.E., Storm, N.J., Diaz, A., et al. (2019). A farnesyltransferase inhibitor activates lysosomes and reduces tau pathology in mice with tauopathy. Sci. Transl. Med. *11*, eaat3005. https://doi.org/10.1126/scitranslmed.aat3005.

Hetz, C., and Saxena, S. (2017). ER stress and the unfolded protein response in neurodegeneration. Nat. Rev. Neurol. *13*, 477–491. https://doi.org/10.1038/nrneurol.2017.99.

Huang, M., Wang, J., Torre, E., Dueck, H., Shaffer, S., Bonasio, R., Murray, J.I., Raj, A., Li, M., and Zhang, N.R. (2018). SAVER: gene expression recovery for single-cell RNA sequencing. Nat. Methods *15*, 539–542. https://doi.org/10.1038/s41592-018-0033-z.

Ibi, D., Nagai, T., Nakajima, A., Mizoguchi, H., Kawase, T., Tsuboi, D., Kano, S.I., Sato, Y., Hayakawa, M., Lange, U.C., et al. (2013). Astroglial IFITM3 mediates neuronal impairments following neonatal immune challenge in mice. Glia *61*, 679–693. https://doi.org/10.1002/glia.22461.

Jiang, S., Wen, N., Li, Z., Dube, U., Del Aguila, J., Budde, J., Martinez, R., Hsu, S., Fernandez, M.V., Cairns, N.J., et al. (2018). Integrative system biology analyses of CRISPR-edited iPSC-derived neurons and human brains reveal deficiencies of presynaptic signaling in FTLD and PSP. Transl. Psychiatry *8*, 265. https://doi.org/10.1038/s41398-018-0319-z.

Karch, C.M., Kao, A.W., Karydas, A., Onanuga, K., Martinez, R., Argouarch, A., Wang, C., Huang, C., Sohn, P.D., Bowles, K.R., et al. (2019). A comprehensive resource for induced pluripotent stem cells from patients with primary tauopathies. Stem Cell Rep. *13*, 939–955. https://doi.org/10.1016/j.stemcr.2019.09.006.

King, M.E., Gamblin, T.C., Kuret, J., and Binder, L.I. (2000). Differential assembly of human tau isoforms in the presence of arachidonic acid. J. Neurochem. *74*, 1749–1757. https://doi.org/10.1046/j.1471-4159.2000.0741749.x.

Korinek, M., Vyklicky, V., Borovska, J., Lichnerova, K., Kaniakova, M., Krausova, B., Krusek, J., Balik, A., Smejkalova, T., Horak, M., and Vyklicky, L. (2015). Cholesterol modulates open probability

and desensitization of NMDA receptors. J. Physiol. *593*, 2279–2293. https://doi.org/10.1113/jphysiol.2014.288209.

Korinek, M., Gonzalez-Gonzalez, I.M., Smejkalova, T., Hajdukovic, D., Skrenkova, K., Krusek, J., Horak, M., and Vyklicky, L. (2020). Cholesterol modulates presynaptic and postsynaptic properties of excitatory synaptic transmission. Sci. Rep. *10*, 12651. https://doi.org/10.1038/s41598-020-69454-5.

Kovacs, G.G. (2020). Astroglia and tau: new perspectives. Front. Aging Neurosci. *12*, 96. https://doi.org/10.3389/fnagi.2020.00096.

Lancaster, M.A., Renner, M., Martin, C.A., Wenzel, D., Bicknell, L.S., Hurles, M.E., Homfray, T., Penninger, J.M., Jackson, A.P., and Knoblich, J.A. (2013). Cerebral organoids model human brain development and microcephaly. Nature *501*, 373–379. https://doi.org/10.1038/nature12517.

Lee, H.K., Velazquez Sanchez, C., Chen, M., Morin, P.J., Wells, J.M., Hanlon, E.B., and Xia, W. (2016). Three dimensional human neuro-spheroid model of Alzhei'er's disease based on differentiated induced pluripotent stem cells. PLoS One *11*, e0163072. https://doi.org/10.1371/journal.pone.0163072.

Liddelow, S.A., Guttenplan, K.A., Clarke, L.E., Bennett, F.C., Bohlen, C.J., Schirmer, L., Bennett, M.L., Münch, A.E., Chung, W.S., Peterson, T.C., et al. (2017). Neurotoxic reactive astrocytes are induced by activated microglia. Nature *541*, 481–487. https://doi.org/10.1038/nature21029.

Lin, Y.T., Seo, J., Gao, F., Feldman, H.M., Wen, H.L., Penney, J., Cam, H.P., Gjoneska, E., Raja, W.K., Cheng, J., et al. (2018). APOE4 causes widespread molecular and cellular Alterations associated with Alzhei'er's disease phenotypes in human iPSC-derived brain cell types. Neuron *98*, 1141–1154.e7. https://doi.org/10.1016/j.neuron.2018.05.008.

Long, J.E., Cobos, I., Potter, G.B., and Rubenstein, J.L.R. (2009). Dlx1&2 and Mash1 transcription factors control MGE and CGE patterning and differentiation through parallel and overlapping pathways. Cereb. Cortex *19*, i96–i106. https://doi.org/10.1093/cercor/bhp045.

Macosko, E.Z., Basu, A., Satija, R., Nemesh, J., Shekhar, K., Goldman, M., Tirosh, I., Bialas, A.R., Kamitaki, N., Martersteck, E.M., et al. (2015). Highly parallel genome-wide expression profiling of individual cells using nanoliter droplets. Cell *161*, 1202–1214. https://doi.org/10.1016/j.cell.2015.05.002.

Madison, B.B. (2016). Srebp2: a master regulator of sterol and fatty acid synthesis. J. Lipid Res. *57*, 333–335. https://doi.org/10.1194/jlr.C066712.

Mathys, H., Davila-Velderrain, J., Peng, Z., Gao, F., Mohammadi, S., Young, J.Z., Menon, M., He, L., Abdurrob, F., Jiang, X., et al. (2019). Single-cell transcriptomic analysis of Alzhei'er's disease. Nature *570*, 332–337. https://doi.org/10.1038/s41586-019-1195-2.

Matias, I., Morgado, J., and Gomes, F.C.A. (2019). Astrocyte heterogeneity: impact to brain aging and disease. Front. Aging Neurosci. *11*, 59. https://doi.org/10.3389/fnagi.2019.00059.

Matsuzaka, T., Shimano, H., Yahagi, N., Kato, T., Atsumi, A., Yamamoto, T., Inoue, N., Ishikawa, M., Okada, S., Ishigaki, N., et al. (2007). Crucial role of a long-chain fatty acid elongase, Elovl6, in obesity-induced insulin resistance. Nat. Med. *13*, 1193–1202. https://doi.org/10.1038/nm1662.

Morel, L., Chiang, M.S.R., Higashimori, H., Shoneye, T., Iyer, L.K., Yelick, J., Tai, A., and Yang, Y. (2017). Molecular and functional properties of regional astrocytes in the adult brain. J. Neurosci. *37*, 8706–8717. https://doi.org/10.1523/JNEUROSCI.3956-16.2017.

Nordberg, A., Rinne, J.O., Kadir, A., and Långström, B. (2010). The use of PET in Alzheimer disease. Nat. Rev. Neurol. *6*, 78–87. https://doi.org/10.1038/nrneurol.2009.217.

Paşca, A.M., Sloan, S.A., Clarke, L.E., Tian, Y., Makinson, C.D., Huber, N., Kim, C.H., Park, J.Y., O'Rourke, N.A., Nguyen, K.D., et al. (2015). Functional cortical neurons and astrocytes from human pluripotent stem cells in 3D culture. Nat. Methods *12*, 671–678. https://doi.org/10.1038/nmeth.3415.

Polioudakis, D., de la Torre-Ubieta, L., Langerman, J., Elkins, A.G., Shi, X., Stein, J.L., Vuong, C.K., Nichterwitz, S., Gevorgian, M., Opland, C.K., et al. (2019). A single-cell transcriptomic atlas of human neocortical development during mid-gestation. Neuron *103*, 785–801.e8. https://doi.org/10.1016/j.neuron.2019.06.011.

Quadrato, G., Nguyen, T., Macosko, E.Z., Sherwood, J.L., Min Yang, S., Berger, D.R., Maria, N., Scholvin, J., Goldman, M., Kinney, J.P., et al. (2017). Cell diversity and network dynamics in photosensitive human brain organoids. Nature *545*, 48–53. https://doi.org/10.1038/nature22047.

Quehenberger, O., Armando, A.M., Brown, A.H., Milne, S.B., Myers, D.S., Merrill, A.H., Bandyopadhyay, S., Jones, K.N., Kelly, S., Shaner, R.L., et al. (2010). Lipidomics reveals a remarkable diversity of lipids in human plasma. J. Lipid Res. *51*, 3299–3305. https://doi.org/10.1194/jlr.M009449.

Raja, W.K., Mungenast, A.E., Lin, Y.T., Ko, T., Abdurrob, F., Seo, J., and Tsai, L.H. (2016). Self-organizing 3D human neural tissue derived from induced pluripotent stem cells recapitulate Alzhei'er's disease phenotypes. PLoS One *11*, e0161969. https://doi.org/10.1371/journal.pone.0161969.

Renner, M., Lancaster, M.A., Bian, S., Choi, H., Ku, T., Peer, A., Chung, K., and Knoblich, J.A. (2017). Self-organized developmental patterning and differentiation in cerebral organoids. EMBO J. *36*, 1316–1329. https://doi.org/10.15252/embj.201694700.

Saher, G., and Stumpf, S.K. (2015). Cholesterol in myelin biogenesis and hypomyelinating disorders. Biochim. Biophys. Acta *1851*, 1083–1094. https://doi.org/10.1016/j.bbalip.2015.02.010.

Shi, Y., Yamada, K., Liddelow, S.A., Smith, S.T., Zhao, L., Luo, W., Tsai, R.M., Spina, S., Grinberg, L.T., Rojas, J.C., et al. (2017). ApoE4 markedly exacerbates tau-mediated neurodegeneration in a mouse model of tauopathy. Nature *549*, 523–527. https://doi.org/10.1038/nature24016.

Sidhaye, J., and Knoblich, J.A. (2021). Brain organoids: an ensemble of bioassays to investigate human neurodevelopment and disease. Cell Death Differ *28*, 52–67. https://doi.org/10.1038/s41418-020-0566-4.

Sloan, S.A., Darmanis, S., Huber, N., Khan, T.A., Birey, F., Caneda, C., Reimer, R., Quake, S.R., Barres, B.A., and Paşca, S.P. (2017). Human astrocyte maturation captured in 3D cerebral cortical

spheroids derived from pluripotent stem cells. Neuron *95*, 779–790.e6. https://doi.org/10.1016/j.neuron.2017.07.035.

Sohn, P.D., Huang, C.T.L., Yan, R., Fan, L., Tracy, T.E., Camargo, C.M., Montgomery, K.M., Arhar, T., Mok, S.A., Freilich, R., et al. (2019). Pathogenic tau impairs axon initial segment plasticity and excitability homeostasis. Neuron *104*, 458–470.e5. https://doi.org/10.1016/j.neuron.2019.08.008.

Stuart, T., Butler, A., Hoffman, P., Hafemeister, C., Papalexi, E., Mauck, W.M., 3rd, Hao, Y., Stoeckius, M., Smibert, P., and Satija, R. (2019). Comprehensive integration of single-cell data. Cell *177*, 1888–1902.e21. https://doi.org/10.1016/j.cell.2019.05.031.

Suzuki, K., Parker, C.C., Pentchev, P.G., Katz, D., Ghetti, B., D'Agostino, A.N., and Carstea, E.D. (1995). Neurofibrillary tangles in Niemann-Pick disease type C. Acta Neuropathol. *89*, 227–238.

Tang, B.L. (2020). Glucose, glycolysis, and neurodegenerative diseases. J. Cell. Physiol. *235*, 7653–7662. https://doi.org/10.1002/jcp.29682.

Tuck, B.J., Miller, L.V.C., Katsinelos, T., Smith, A.E., Wilson, E.L., Keeling, S., Cheng, S., Vaysburd, M.J., Knox, C., Tredgett, L., et al. (2022). Cholesterol determines the cytosolic entry and seeded aggregation of tau. Cell Rep. *39*, 110776. https://doi.org/10.1016/j.celrep.2022.110776.

van der Kant, R., Langness, V.F., Herrera, C.M., Williams, D.A., Fong, L.K., Leestemaker, Y., Steenvoorden, E., Rynearson, K.D., Brouwers, J.F., Helms, J.B., et al. (2019). Cholesterol metabolism is a druggable Axis that independently regulates tau and amyloid-β in iPSC-derived Alzhei'er's disease neurons. Cell Stem Cell *24*, 363–375.e9. https://doi.org/10.1016/j.stem.2018.12.013.

Velmeshev, D., Schirmer, L., Jung, D., Haeussler, M., Perez, Y., Mayer, S., Bhaduri, A., Goyal, N., Rowitch, D.H., and Kriegstein, A.R. (2019). Single-cell genomics identifies cell type-specific molecular changes in autism. Science *364*, 685–689. https://doi.org/10.1126/science.aav8130.

Wang, P., Zhang, H., Wang, Y., Zhang, M., and Zhou, Y. (2020). Plasma cholesterol in Alzheimer's disease and frontotemporal dementia. Transl. Neurosci. *11*, 116–123. https://doi.org/10.1515/tnsci-2020-0098.

Wilson, D.M., and Binder, L.I. (1997). Free fatty acids stimulate the polymerization of tau and amyloid beta peptides. In vitro evidence for a common effector of pathogenesis in Alzhei'er's disease. Am. J. Pathol. *150*, 2181–2195.

Yoon, S.J., Elahi, L.S., Paşca, A.M., Marton, R.M., Gordon, A., Revah, O., Miura, Y., Walczak, E.M., Holdgate, G.M., Fan, H.C., et al. (2019). Reliability of human cortical organoid generation. Nat. Methods *16*, 75–78. https://doi.org/10.1038/s41592-018-0255-0.

Zamanian, J.L., Xu, L., Foo, L.C., Nouri, N., Zhou, L., Giffard, R.G., and Barres, B.A. (2012). Genomic analysis of reactive astrogliosis. J. Neurosci. *32*, 6391–6410. https://doi.org/10.1523/JNEUROSCI.6221-11.2012.

Zhao, J., Lu, W., Ren, Y., Fu, Y., Martens, Y.A., Shue, F., Davis, M.D., Wang, X., Chen, K., Li, F., et al. (2021). Apolipoprotein E regulates lipid metabolism and α-synuclein pathology in human iPSC-derived cerebral organoids. Acta Neuropathol. *142*, 807–825. https://doi.org/10.1007/s00401-021-02361-9.

Stem Cell Reports, Volume *17*

Supplemental Information

Human tau mutations in cerebral organoids induce a progressive dys-homeostasis of cholesterol

Stella M.K. Glasauer, Susan K. Goderie, Jennifer N. Rauch, Elmer Guzman, Morgane Audouard, Taylor Bertucci, Shona Joy, Emma Rommelfanger, Gabriel Luna, Erica Keane-Rivera, Steven Lotz, Susan Borden, Aaron M. Armando, Oswald Quehenberger, Sally Temple, and Kenneth S. Kosik

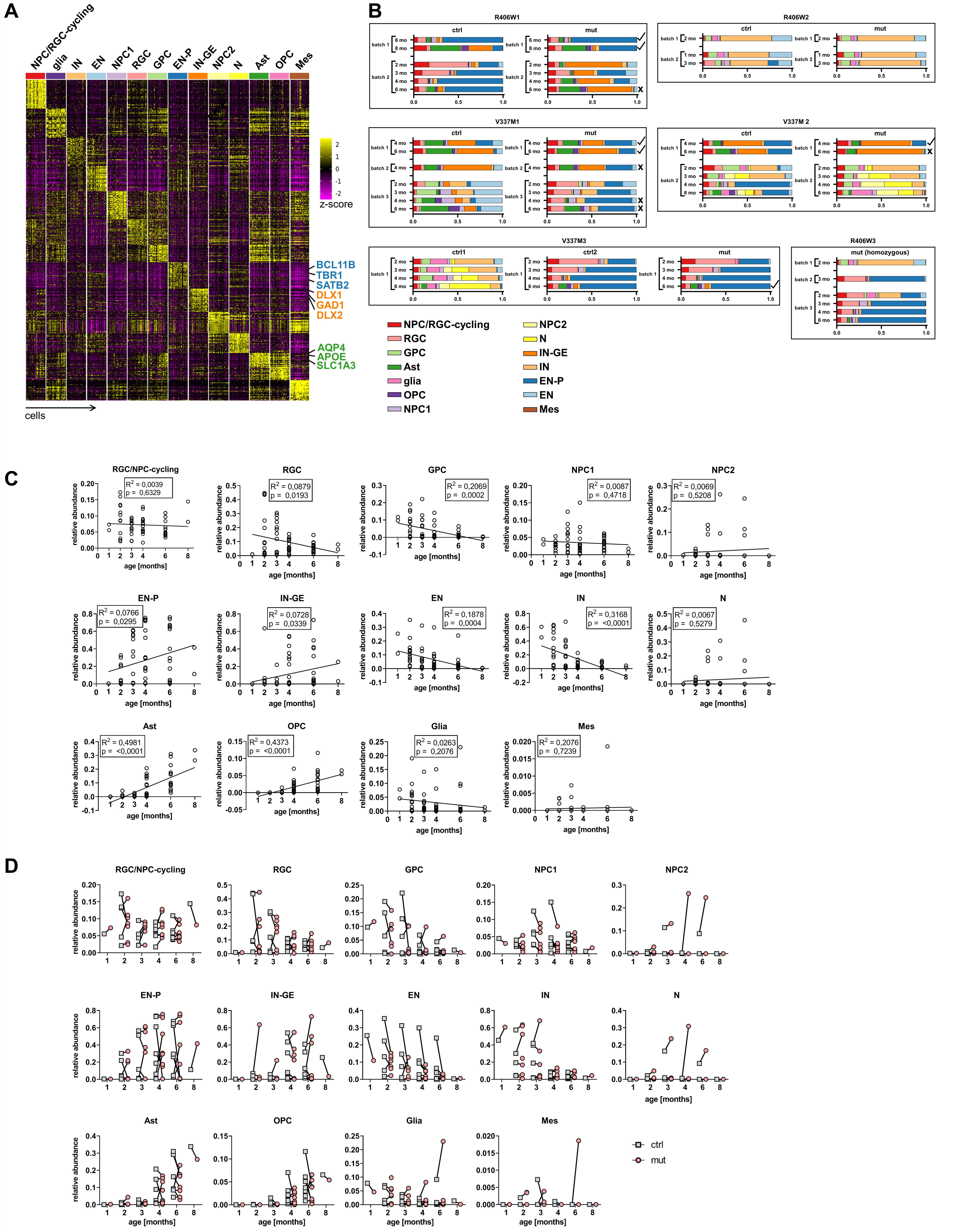

Supplemental Figure 1 – Legend on next page

Supplemental Figure 1, related to Figure 1 Cellular composition of cerebral organoids A) Heatmap showing expression levels (z-scores) of the top 50 marker genes for each cell class. Cell classes were randomly downsampled to 50 cells to improve visualization of small clusters. Highlighted are selected canonical markers of EN-P (blue), IN-GE (orange) and Ast (green). **B)** Cellular composition of all individual samples sequenced. For full line names and their abbreviations, see Methods. Shown are relative abundances (number of cells per type relative to all cells in a sample). For isogenic pairs that have ≥ 30 astrocytes in each sample (and were thus further analyzed for transcriptional effects of *MAPT* mutations of astrocytes, Figure 3), it is indicated whether the isogenic pair had > 5% pyramidal neurons (EN-P) and < 50% neurons belonging to the unidentified neuronal populations (IN, EN and N) in each sample of the pair (check mark) or not ("x"). **C)** Changes in relative abundances of cell types over time. Each datapoint represents one individual sample. Simple linear regressions were calculated for each cell type. **D)** Relative abundances of cell types over time, separated by control and mutant samples. Lines were used to visually connect members of isogenic pairs. Statistically significant differences between ctrl and mut samples were calculated for each cell type at ages 2, 3, 4 and 6 months using two-tailed Wilcoxon matched-pairs signed rank tests and Bonferroni correction for multiple testing. All comparisons were non-significant.

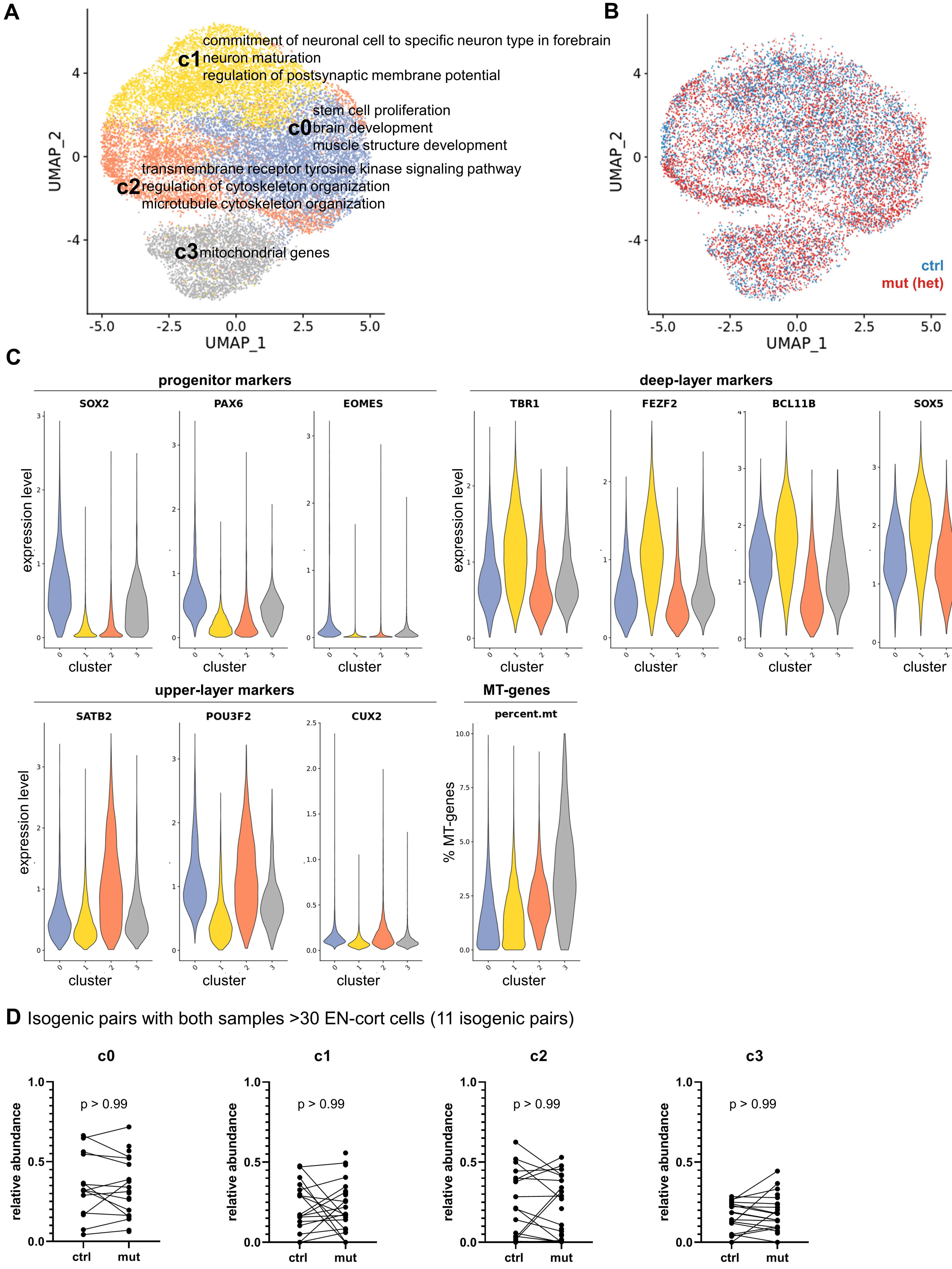

Supplementary Figure 2 – Legend on next page

Supplemental Figure 2, related to Figure 2 Subclustering of pyramidal excitatory neurons A) UMAP plot of pyramidal excitatory neurons after extraction from entire dataset and subclustering (see Methods), yielding 4 clusters. Gene ontology enrichment analysis using the database "biological process" was performed on the marker genes of each subcluster. Shown are the top 3 enriched gene categories for each cluster. **B)** UMAP colored by control / mutant cells, showing that they are largely intermingled. **C)** Expression values of cortical layer markers specific to progenitors, deep-layer neurons and upper layer neurons, showing enrichment of deep-layer and upper-layer markers in cluster 1 and 2, respectively. Cluster 0 is enriched in progenitor markers. **D)** Analysis of enrichment/depletion of mutant pyramidal excitatory neurons in the identified subclusters. All isogenic pairs of which both members have at least 30 pyramidal neurons were analyzed (11 isogenic pairs) using Wilcoxon matched pairs signed rank test followed by Benjamini-Hochberg correction, returning non-significant p-values. Members of isogenic pairs are connected by lines. Unconnected datapoints are from the homozygous R406W mutation line and were not included in statistics.

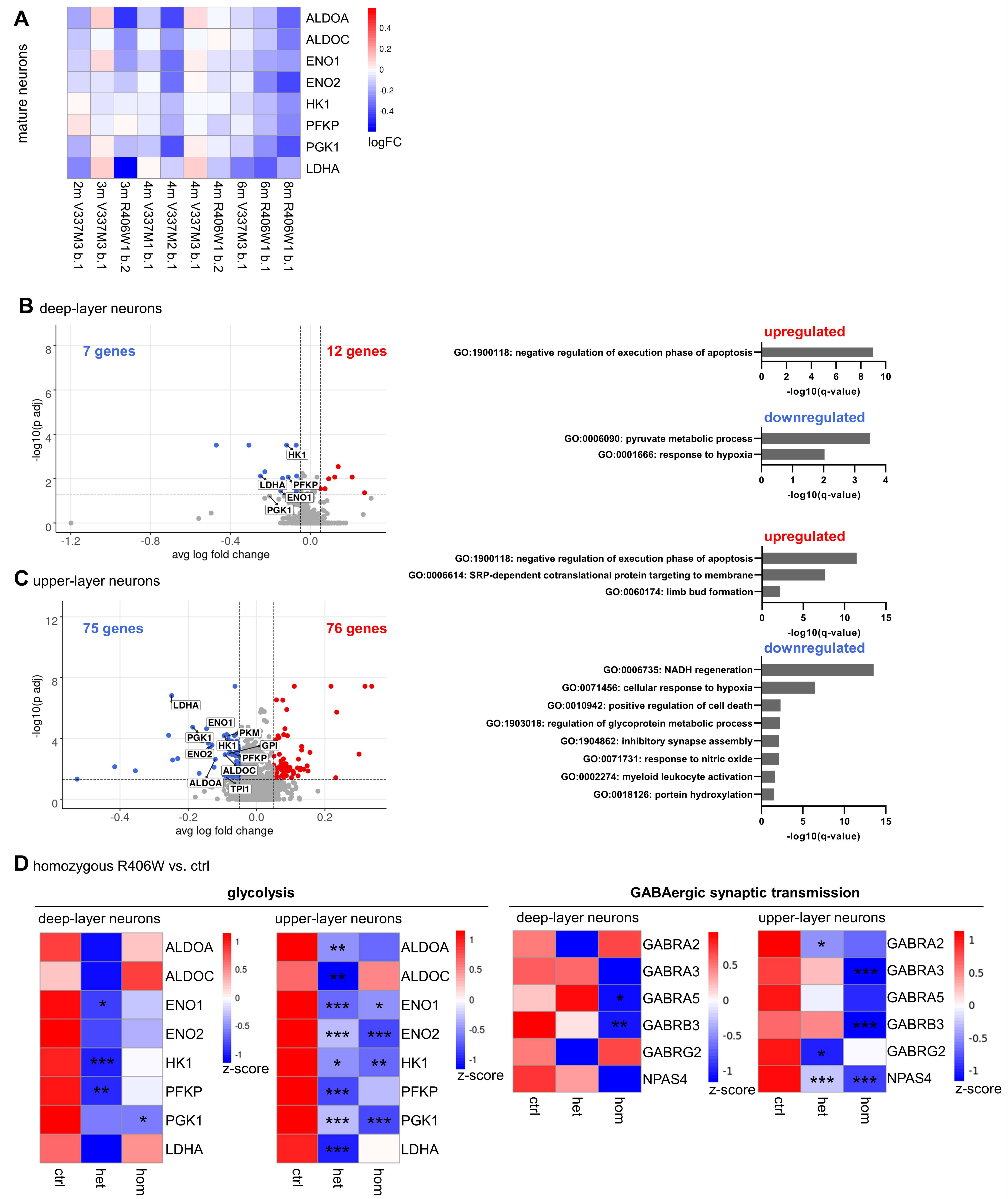

Supplemental Figure 3 – Legend on next page

Supplemental Figure 3, related to Figure 2 Differential gene expression analysis in pyramidal excitatory neurons. A) Heatmap of logFC of glycolysis related genes identified in Fig. 2C across all isogenic pairs with > 30 pyramidal neurons per sample. Abbreviations b.1 and b.2 refer to the different differentiation batches for each line as depicted in Supplementary Figure 1B. **B)** Differential gene expression analysis in deep-layer neurons (n = 5 isogenic pairs from 4 organoid batches, 1024 cells), using the MAST differential expression test with a logFC cutoff of 0.05. Benjamini-Hochberg corrected p-values < 0.05 were considered statistically significant. Left: volcano plot, differentially expressed genes involved in glycolysis are highlighted; Right: Enriched GO terms ("biological process") of upregulated and downregulated genes. **C)** Differential gene expression analysis in upper-layer neurons (n = 6 isogenic pairs from 4 organoid batches, 1530 cells), using the MAST test as described in (B). Note that *MAPT* mutant neurons of both deep- and upper-layer clusters show enrichment of downregulated genes in categories related to glycolysis (e.g. pyruvate metabolic process, glucose homeostasis, NADH regeneration). **D)** Validation of transcriptional changes observed in heterozygous R406W and V337M mutants in the R406W homozygous mutant line. Genes of the glycolytic pathway (left) and involved in GABAergic synaptic transmission (right) identified as differentially expressed heterozygous mutant vs. ctrl neurons (deep- and upper-layer neurons combined, see Figure 3) are shown. Heatmaps show expression levels (z-scores) of the selected genes in control, heterozygous (V337M and R406W) and homozygous (R406W) mutant cells, separately for deep- and upper-layer neurons enriched clusters. All genes in the dataset were tested for differential expression using the MAST test as described in (B). Significantly different expression compared to controls is indicated: * $p \leq 0.05$, ** $p \leq 0.01$, *** $p \leq 0.001$

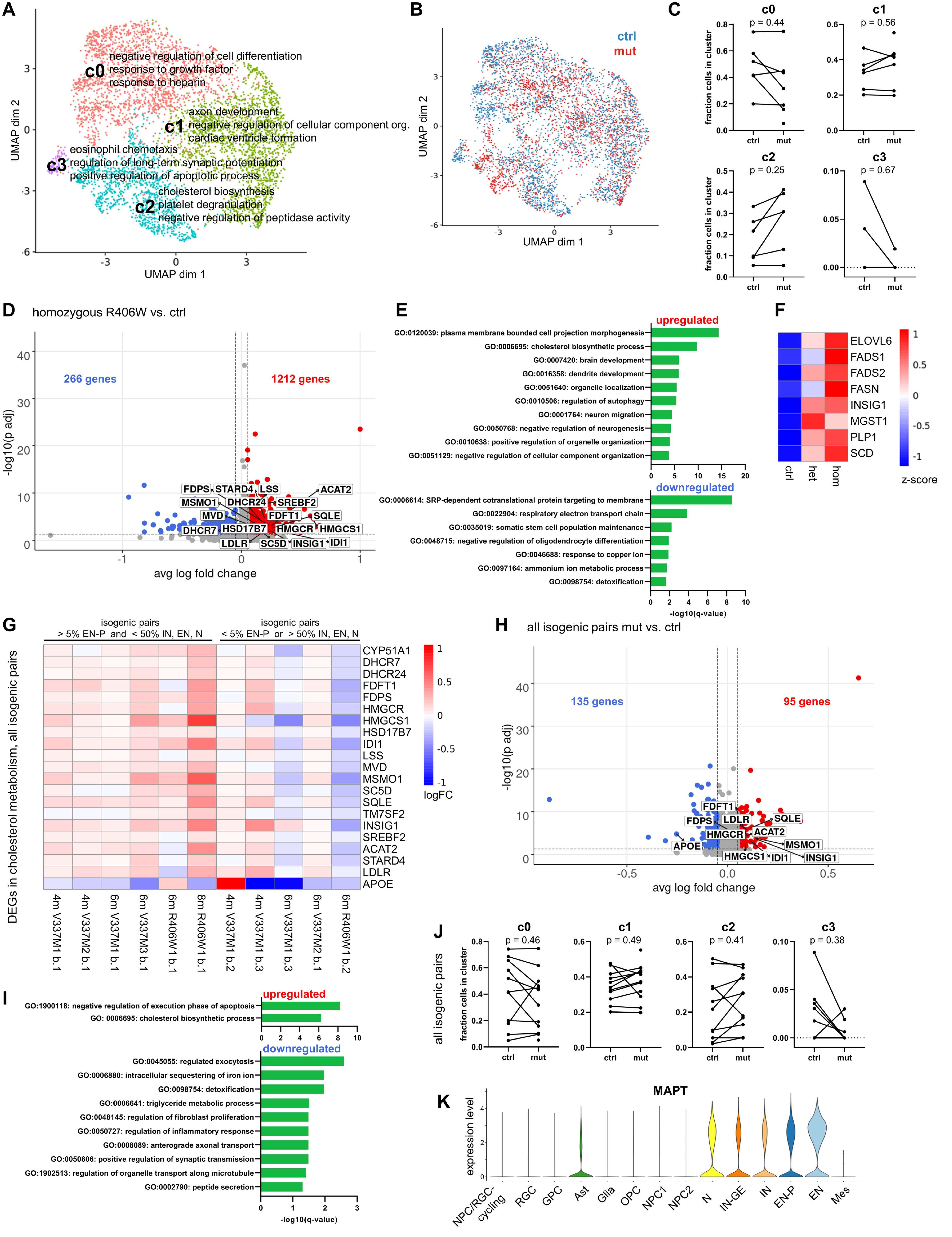

Supplemental Figure 4 – Legend on next page

Supplemental Figure 4, related to Figure 3 Single-cell analysis of astrocytes - extended A) UMAP of astrocytes that were extracted from the dataset and subclustered (see Methods), resulting in 4 clusters. Gene ontology enrichment analysis using the database "biological process" was performed on the marker genes of each subcluster. Shown are the top 3 enriched gene categories for each cluster. **B)** UMAP colored by control / mutant cells, showing that they are largely intermingled. **C)** Analysis of enrichment/depletion of mutant astrocytes in the subclusters. All isogenic pairs of which both members have at least 30 astrocytes were analyzed (6 isogenic pairs) using Wilcoxon matched pairs signed rank test followed by Benjamini-Hochberg correction. All statistical tests returned non-significant p-values. Members of isogenic pairs are connected by lines. Unconnected datapoints are from the homozygous R406W mutation and were not included in the statistics. **D)** Volcano plot of differential gene expression analysis of astrocytes of the homozygous R406W mutant line vs. ctrl astrocytes (n = 1053 cells), using the MAST test with a logFC cutoff of 0.05. Benjamini-Hochberg corrected p-values < 0.05 were considered statistically significant. Differentially expressed genes involved in cholesterol metabolism are highlighted. **E)** Gene ontology enrichment analysis of genes identified in (D). The GO term "cholesterol biosynthetic process" was enriched in genes upregulated in the homozygous R406W mutant. **F)** Heatmap showing expression levels (z-scores) of genes included in the GO term "fatty acid biosynthetic process" identified as differentially expressed in Figure 3A in control, heterozygous (R406W and V337M) and homozygous (R406W) *MAPT* mutant astrocytes. All genes 8 genes are significantly upregulated in heterozygous mutants compared to control, and in the homozygous mutant compared to control. **G)** Heatmap showing log fold changes of differentially expressed genes that are involved cholesterol metabolism for all individual isogenic pairs. Positive log fold changes are more consistent in the isogenic pairs that have > 5% pyramidal neurons (EN-P) and < 50% neurons belonging to the unidentified neuronal populations (IN, EN and N) in each sample. Abbreviations b.1, b.2 and b.3 refer to the different batches as depicted in Supplementary Figure 1B. **H)** Volcano plot of differential gene expression analysis in all isogenic pairs (n = 11 isogenic pairs from 7 organoid batches, 3398 cells), omitting filtering and using the MAST differential expression test as described in (D). Differentially expressed genes involved in cholesterol metabolism are highlighted. **I)** Gene ontology enrichment analysis of genes identified in (H), demonstrating that the GO term "cholesterol biosynthetic process" was enriched also in genes upregulated when testing the unfiltered dataset for differentially expressed genes. **J)** Analysis for enrichment/depletion of *MAPT* mutant cells as described in (C) but on all isogenic pairs without applying a filter. All statistical tests returned non-significant p-values. **K)** Expression level of *MAPT* mRNA across different cell types, showing presence of *MAPT* mRNA in astrocytes. Unimputed (non-SAVER treated) expression values are shown.

A

Cholesterol and biosynthesis intermediates		
abbreviation	full name	detected in n organoids
7-DHC	7-Dehydrocholesterol	50 / 60
8-DHC	8-Dehydrocholesterol	60 / 60
DHL	24,25-Dihydrolanosterol	60 / 60
T-MAS	14-Demethyl-lanosterol	54 / 60
FF-MAS	14-demethyl-14-dehydrolanosterol	0 / 60
Dihydro FF-MAS	4,4-dimethyl-cholest-8(9), 14-dien-3ß-ol	0 / 60
Dihydro T-MAS	4,4-dimethyl-cholest-8(9)-en-3ß-ol	49 / 60
Cholesterol	Cholesterol	60 / 60
Desmosterol	Desmosterol	60 / 60
Lanosterol	Lanosterol	60 / 60
22-OHC	22-Hydroxycholesterol,	0 / 60
Zymostenol	Zymostenol	0 / 60
Zymosterol	Zymosterol	0 / 60

Oxysterols		
abbreviation	full name	detected in n organoids
24-OHC	24-Hydroxycholesterol	32 / 60
24,25-EpC	24,25-Epoxycholesterol	0 / 60
25-OHC	25-Hydroxycholesterol	0 / 60
27-OHC	27-Hydroxycholesterol	10 / 60
4b-OHC	4ß-Hydroxycholesterol	0 / 60
7a-OHC	7alpha-Hydroxycholesterol	0 / 60
7,25-dOHC	7,25-Dihydroxycholesterol	0 / 60
7,27-dOHC	7,27-Dihydroxycholesterol	5 / 60
7-KetoC	7-Ketocholesterol	0 / 60

Other sterols		
abbreviation	full name	detected in n organoids
7a-OH-ONE	7alpha-hydroxy-4-cholesten-3-one	0 / 60
Cholestanol	Cholestanol	60 / 60
Campesterol	Campesterol	60 / 60
Ergosterol	Ergosterol	0 / 60
Sitosterol	Sitosterol	54 / 60

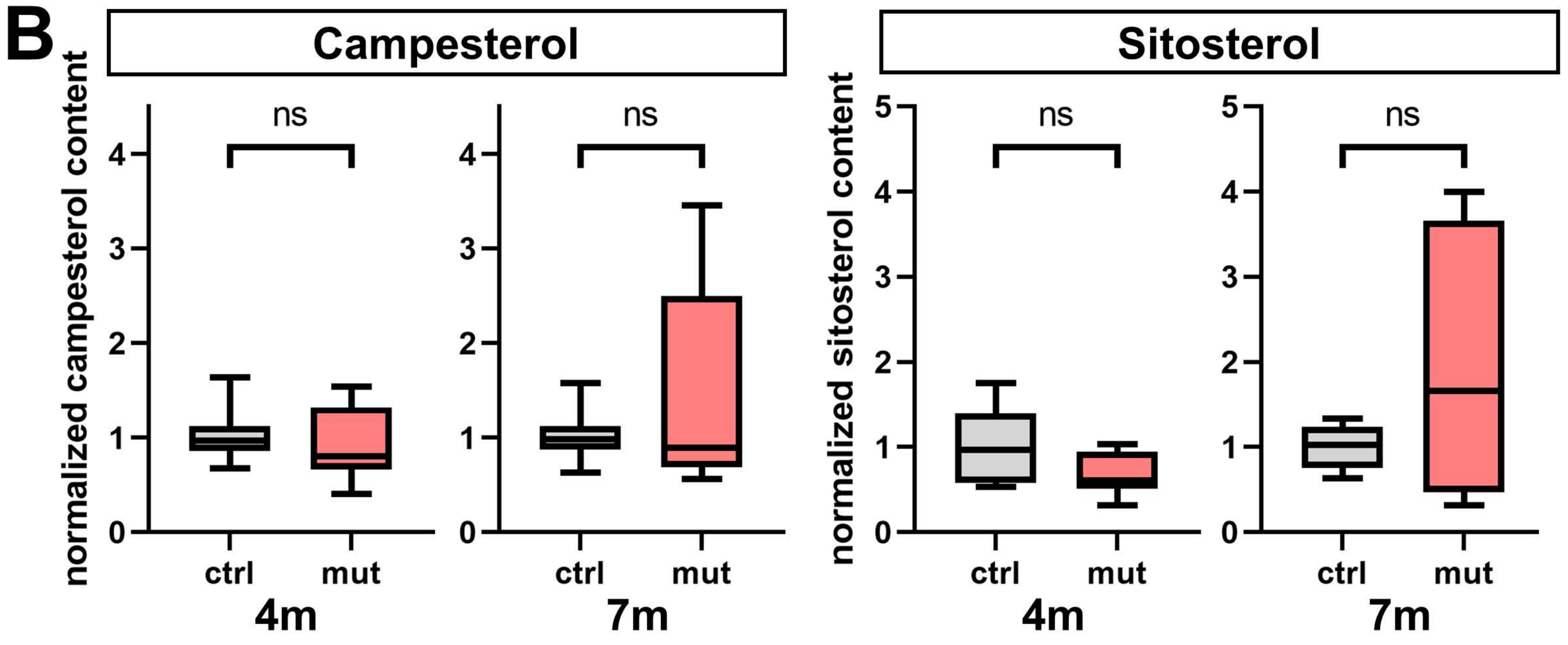

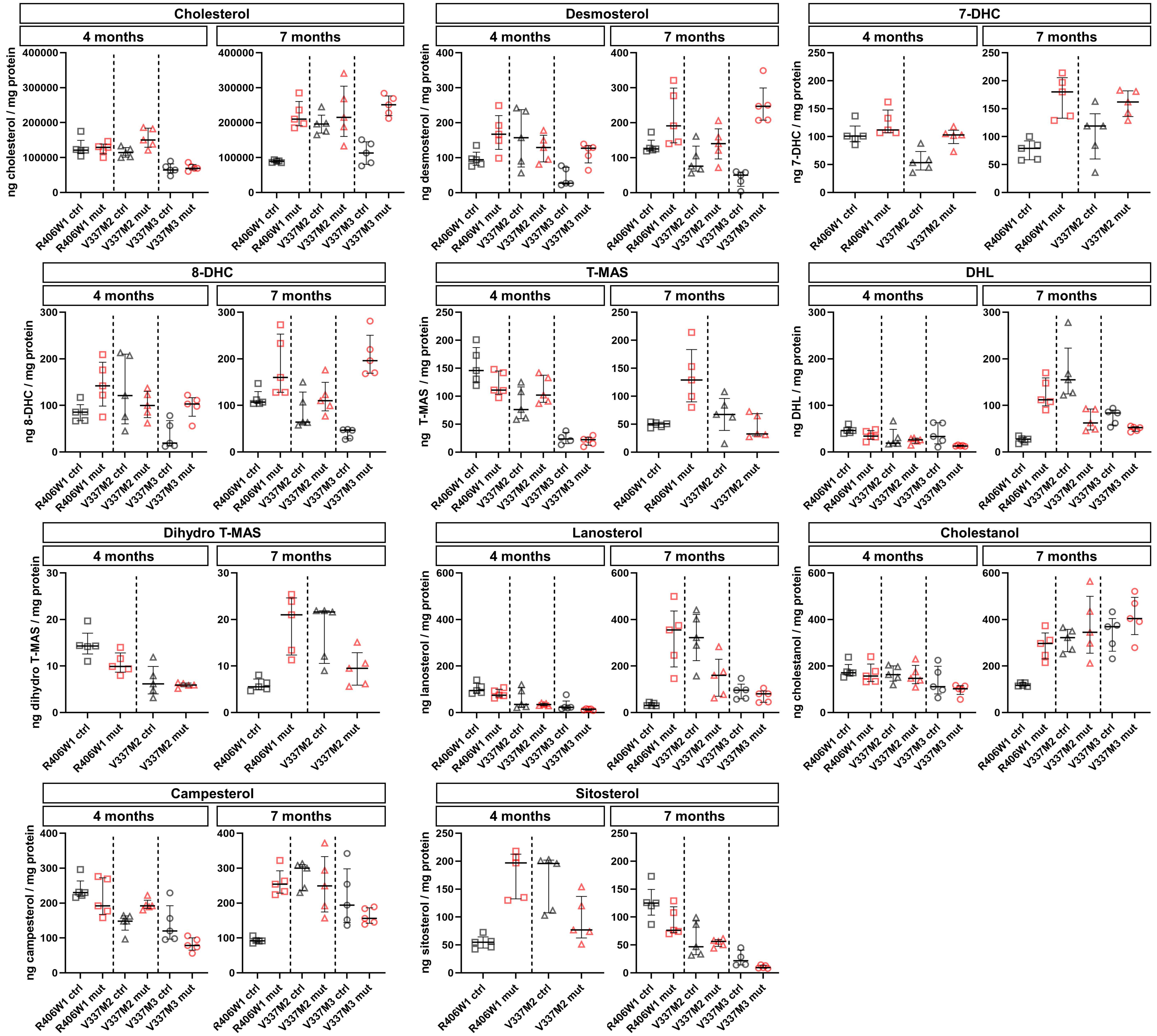

Supplemental Figure 5, related to Figure 4 Sterol quantification in *MAPT* mutant organoids - extended. A) List of compounds contained in the sterol panel and their detection rates in organoid samples. **B)** LC-MS data of the two phytosterols detected, all three isogenic lines combined. Sitosterol: 4 months ctrl n = 14 from 3 organoid batches, 4 months mut n = 15 from 3 organoid batches, 7 months ctrl n = 10 from 2 organoid batches, 7 months mut n = 10 from 2 organoid batches. Campesterol: n = 15 from 3 organoid batches for each condition and timepoint. Mut were normalized to ctrl values. Box and whiskers plots represent the median (line in box center), first and third quartile (lower and upper box border, respectively), and minimum and maximum values (whiskers). Wilcoxon rank-sum test followed by Benjamini-Hochberg correction (for 22 multiple comparisons) revealed no statistically significant differences between ctrl and mut. **C)** Representation of LC-MS data for individual isogenic pairs and samples. For full line names, see Methods. Data are expressed at ng / mg protein. Plots display the median (line), first and third quartile ranges (whiskers) and individual data points.

Supplemental Table Legends

Supplemental Table 1, related to Figure 1 Marker genes of identified cell populations. The reported marker genes were identified using the Wilcoxon rank sum test, with a logFC threshold of 0.25 and Bonferroni correction for multiple testing.

Supplemental Table 2, related to Figure 1 GO categories enriched in cell population markers. Results were obtained by performing GO enrichment analysis on the top 50 markers of each population.

Supplemental Table 3, related to Figures 2 and 3 Lists of differentially expressed genes in *MAPT* mutant versus control cells. For differential gene expression analyses, the MAST test was used. Genes with Benjamini-Hochberg corrected p-values < 0.05 and logFC > 0.05 or < -0.05 are reported.

Supplemental Experimental Procedures

Cell Lines and Maintenance of Human Induced Pluripotent Stem Cells (iPSCs)

The iPSC lines used in this study: GIH6-1-C1Δ1E11 (WT/WT), GIH6-1-C1Δ1A02 (V337M/WT), GIH7-C2Δ2B12 (WT/WT), GIH7-C2Δ2F02 (WT/WT), GIH7-C2Δ2A01 (V337M/WT), ND32951A.15Δ1B06 (WT/WT), and ND32951A.15Δ1B09 (V337M/WT), GIH143-C3 (R406W/R406W, homozygote), F11362.1ΔC11 (WT/WT), F11362.1ΔF10 (R406W/WT), F11421.12Δ2A07 (WT/WT), F11421.12 (R406W/WT), were established from the Tau Consortium iPSC line collection (Karch et al. 2019) and grown at NSCI core facility NeuraCell and are available upon request (www.neuralsci.org/tau). Short names for the isogenic pairs were used throughout the manuscript as follows: R406W1: F11362.1ΔC11 (WT/WT) and F11362.1ΔF10 (R406W/WT); R406W2: F11421.12Δ2A07 (WT/WT) and F11421.12 (R406W/WT); V337M1: ND32951A.15Δ1B06 (WT/WT), and ND32951A.15Δ1B09 (V337M/WT); V337M2: GIH6-1-C1Δ1E11 (WT/WT) and GIH6-1-C1Δ1A02 (V337M/WT); V337M3: GIH7-C2Δ2B12 (WT/WT), GIH7-C2Δ2F02 (WT/WT) and GIH7-C2Δ2A01 (V337M/WT). APOE genotypes are as follows. E3/E3 genotype: GIH6-1-C1Δ1E11, GIH6-1-C1Δ1A02, GIH7-C2Δ2B12, GIH7-C2Δ2F02, GIH7-C2Δ2A01, GIH143-C3, F11421.12Δ2A07 and F11421.12; E4/E4 genotype: ND32951A.15Δ1B06 and ND32951A.15Δ1B09; E2/E3 genotype: F11362.1ΔC11 and F11362.1ΔF10. The iPSC lines were expanded with daily feeding of mTeSR1 medium (StemCell Technologies, catalog #05851) or mTESR1 with FGF2-Discs (StemCultures catalog #DSC500) and feeder-free conditions in six-well plates (Corning, catalog #3506) precoated with growth factor-reduced Matrigel (Corning, catalog #356231). iPSCs were passaged every week using Dispase (STEMCELL Technologies Cat#07923) or ReLeSR (Stem Cell Tech catalog #100-0484).

3D Cerebral Organoid Production

Following previously described protocols (Yoon et al. 2019; Gregory et al. 2020), organoid production was started in AggreWell™800 plates (Stem Cell Technologies, catalog #34811). The wells were rinsed with 2 ml of DMEM/F12/well, then aspirated. 0.5 ml of organoid formation medium (Essential 8 (E8) medium (ThermoFisher, cat. #A1517001) supplemented with ROCK inhibitor - Y-27632 (Tocris Cat#1254) diluted1:1000 to 10 µM) was added to each well. The plates were centrifuged at 2,000 x g for 5 minutes in a swinging bucket rotor fitted with a plate holder and inspected under a microscope to ensure any bubbles were removed. iPSCs were grown to 80-85% confluency. To obtain a single-cell suspension of iPSCs for effective organoid formation, the cells were pre-treated with 10 µM of Y-27632 in E8 medium

for 50-60 minutes. The culture medium was removed from the wells, which were rinsed twice with DPBS (Dulbecco's Phosphate Buffered Solution without Calcium or Magnesium: Gibco, cat. #14190-144). 2 ml of Accutase were added to each well and incubated for c.10 minutes at 37°C in 5% CO_2 until cells lifted off the dish by gentle shaking. The cell suspension was gently triturated 2-3 times to dissociate remaining cell clumps. The cells were counted, centrifuged at 1,200 rpm for 4 minutes, and resuspended in organoid formation medium to achieve a single-cell suspension of 3 million cells in 1.5 ml which was added to each AggreWell to give a final volume of 2 ml per well. The suspension was gently pipetted to evenly distribute cells throughout each well. The plate was centrifuged for 3 minutes at 100xg to dispense the cells into the microwell cones to achieve even distribution, which was checked under the microscope. The cells were incubated for 24 hours at 37°C and 5% CO_2.

After 24 hours of incubation, the 3D organoids were removed from the AggreWell™800 plate, rinsed twice in DMEM/F12 medium then transferred into ultra-low attachment 10 cm plates in medium A: E6 medium supplemented with Dorsomorphin (DM) (Tocris, catalog #3093) at 2.5 µM;SB431542 (Tocris, catalog #1614) at 10 µM, and XAV-939 (Tocris #3748) at 2.5 µM.

After day 1, medium A changes were performed daily. On day 6, the medium was changed to medium B: Neurobasal-A (Life Technologies #10888-022) supplemented with B-27 supplement without vitamin A (Life Technologies, catalog #12587010), Anti-A (Life Technologies, catalog #15240-062), 20 ng/ml FGF2 (R&D Systems, catalog #233-FB), GlutaMax (Life Technologies, catalog #3505-061), and 20 ng/ml EGF (Peprotech, catalog # AF-100-15). The organoids were fed with medium B, daily for the first 10 days and every other day for the subsequent 9 days. Starting at day 25, medium C was used: Neurobasal-A (Life Technologies #10888-022) supplemented with B-27 supplement without vitamin A (Life Technologies, catalog #12587010), Anti-A (Life Technologies, catalog #15240-062), GlutaMax(Life Technologies, catalog #3505-061), 20 ng/ml BDNF (Peprotech, catalog # 450-02) and 20 ng/ml NT3 (Peprotech, catalog # 450-03), every other day. From day 43 onwards, medium D was used: the same as C but without BDNF or NT3 and changed every four days. Throughout the culture period, organoids that fused together were separated by cutting with a disposable scalpel (McKesson non-safety scalpels, 1626). Organoids in Figures 4, iPSCs were grown and patterned as described above with minor modifications, culturing in ultra-low attachment 96 well U-bottom plates (S-BIO, catalog #MS9096SZ).

Single-cell RNA Sequencing of Cerebral Organoids

Organoid Samples

For the V337M mutant line GIH7 (GIH7-C2Δ2A01), organoids of two different CRISPR-corrected clones (GIH7-C2Δ2B12 and GIH7-C2Δ2F02) were sequenced. For all other heterozygous mutant lines (F11362.1.ΔF10, F11421.12parent, ND32951A.15Δ2B09 and GIH6-1-C1ΔA02), one CRISPR corrected clone each was used as control line (F11362.1.ΔC11, F11421.12ΔA07, ND32951A.15Δ1B06 and GIH6-1-C1ΔE11). We also sequenced organoids from a homozygous carrier of the R406W mutation (GIH143-C3). We sampled organoids at ages 2, 3, 4 and 6 months, except for the R406W mutant line F11421 which was sampled up to 3 months. One isogenic pair (R406W mutant F11362.1.ΔF10 and isogenic control F11362.1.ΔC11) was also sampled at 8 months, and one isogenic pair (R406W mutant F11421.12parent and isogenic control F11421.12ΔA07) at 1 month (Figure 1B). One replicate (defined as one differentiation experiment) of each line at each timepoint was sequenced, with a few exceptions where we sequenced 2 or 3 replicates (Figure 1B).

Organoid Dissociation

3-4 organoids were pooled for each dissociation and dissociated using the Worthington Papain Dissociation System (Worthington). Solutions were prepared according to the Manufacturer's instructions. Organoids in FB "D" media were cut into pieces with a sterile scalpel blade and washed twice in PBS prior to dissociation. Incubation in papain/DNaseI solution was performed in a 15 ml conical tube with untightened lid and at 37°C and 5% CO_2 with occasional agitation. After the first 30 min of incubation, organoids were triturated 10 times with a fire-polished glass Pasteur pipette, followed by further incubation and trituration every 15 min until dissociation was complete. The total incubation time in papain/DNaseI solution was 75 -90 min. After adding Inhibitor Solution and DNaseI in EBSS, the dissociated cells were pelleted by centrifugation for 5 min at 300 g. The cells were resuspended in ice-cold PBS/0.01% BSA and passed through a 35 µm cell strainer. An aliquot of the single-cell suspension was mixed with Trypan blue and analyzed for viability and cell count. Viability was > 90% for all organoid samples.

Drop-seq Library Construction

Following dissociation, cells were diluted to 110 cells/µl in PBS/0.01% BSA. Drop-seq was performed as described (Macosko et al., 2015, full details available under http://mccarrolllab.org/download/905/). Briefly, cells and barcoded beads (Chemgenes, 132 beads/ul in lysis buffer) were ran on an aquapel-treated microfluidic drop-seq device (FlowJEM) for co-encapsulation in nanoliter-sized droplets. After

droplet-breakage, reverse transcription and Exonuclease I treatment, beads were counted, and 2000 beads were apportioned per PCR tube for cDNA amplification. The amplified cDNA libraries were purified using SPRI beads (Beckman Coulter) and quantified on a Fragment Analyzer (Agilent). Tagmentations were performed using the Illumina Nextera XT kit (Illumina), and the resulting libraries were purified in two consecutive rounds of SPRI beads-based size selection (0.6x beads to sample ratio followed by 1x beads to sample ratio). The size and concentration of the final libraries were measured on a Fragment Analyzer and a Qbit Fluorometer (ThermoFisher), respectively. Libraries were sequenced on an Illumina Nextseq500 instrument at 50,000 reads per cell.

Single-cell RNA Data Analysis of Cerebral Organoids

Counts Matrix Generation

Counts matrices were generated using the Drop-seq tools package (Macosko et al. 2015), with full details available online (https://github.com/broadinstitute/Drop-seq/files/2425535/Drop-seqAlignmentCookbookv1.2Jan2016.pdf). Briefly, raw reads were converted to BAM files, cell barcodes and UMIs were extracted, and low-quality reads were removed. Adapter sequences and polyA tails were trimmed, and reads were converted to Fastq for STAR alignment (STAR version 2.6). Mapping to human genome (hg19 build) was performed with default settings. Reads mapped to exons were kept and tagged with gene names, beads synthesis errors were corrected, and a digital gene expression matrix was extracted from the aligned library. We extracted data from twice as many cell barcodes as the number of cells targeted (NUM_CORE_BARCODES = 2x # targeted cells).

Filtering Low-Quality Cells and Doublets

Downstream analysis was performed using Seurat 3.0 (Butler et al. 2018; Stuart et al. 2019) in R version 3.6.3. An individual Seurat object was generated for each sample which were filtered and clustered individually. This is because the Doublet Finder method (see below) identifies doublets more accurately in individual samples when they have different cellular compositions. Cells with < 300 genes detected were filtered out, as were cells with > 10% mitochondrial gene content. Counts data were log-normalized using the default NormalizeData function and the default scale of 1e4. Then, the top 2000 variable genes were identified using the Seurat FindVariableFeatures function (selection.method = “vst”, nfeatures = 2000), followed by scaling and centering using the default ScaleData function. Principal Components Analysis was carried out on the scaled expression values of the 2000 top variable genes, and the cells were clustered using the first 50 principal components (PCs) as input in the FindNeighbors function, and a resolution of 0.4 in the FindClusters function. Non-linear dimensionality reduction was performed by

running UMAP on the first 50 PCs. Following clustering and dimensionality reduction, putative cell doublets were identified using DoubletFinder (McGinnis et al. 2019), assuming a doublet formation rate of 5%. For each sample, the optimal pK value was identified based on the results of paramSweep_vs, summarizeSweep and find.pK functions of the DoubletFinder package. Instead of using the default paramSweep_vs function, we extended the upper range of computed pK values to 1.2. We visually verified cells identified as doublets had high nFeatures (number of genes expressed) by plotting the pANN metric against nFeatures. For samples not showing this correlation, we adjusted the pK value to the next highest peak in the pK/BCmetric plot. Finally, the individual Seurat objects were merged, and doublets removed. The resulting dataset had a mean of 1499 transcripts per cell and 930 genes per cell. The mean content of mitochondrially-encoded genes was 2.1%.

Data Imputation

Raw counts of the merged Seurat object were extracted using the GetAssayData function (slot = "counts"). Imputation was performed using the saver Function of the SAVER package (Huang et al. 2018) with default settings. A new Seurat object was generated from the resulting matrix.

Clustering and Cell Type Identification

Normalization, variable gene selection, and scaling on the imputed dataset were performed as above with the exception that 3000 variable genes were identified. Statistically significant principal components were determined using the JackStraw and ScoreJackstraw functions of Seurat v3, revealing a drop-off of p-values below significance level of 0.05 between PCs 90-100. Consequently, clustering and UMAP dimensionality reduction were performed on the first 90 PCs. Clustering at resolution 0.6 resulted in 17 clusters in good agreement with the expression of known marker genes for cell types found in brain organoids. Marker genes for each cluster were identified using the Wilcoxon rank sum test implemented in the FindMarkers function, with a logFC threshold of 0.25 and Bonferroni correction for multiple testing.

Marker genes for each cluster were manually compared to known marker genes, and to published single-cell data of the human brain and brain organoids (Giandomenico et al. 2019; Polioudakis et al. 2019; Quadrato et al. 2017; Velmeshev et al. 2019). Clusters 1 and 10 were merged into the "IN" cluster (inhibitory neurons most likely not derived from ganglionic eminences), since they show very low expression of ganglionic eminences markers (DLX1,2,5,6 and DLX6-AS1), but share GAD1 as marker gene. Similarly, clusters 3, 4, and 5 were merged to form the "EN-P" cluster (excitatory cortical neurons), based on the common expression of marker genes BCL11B, TBR1 and SOX5. This merging of clusters resulted in 14 major subpopulations. The list of marker genes for each cluster is provided as Supplementary Table 1.

Subclustering of Astrocytes and Pyramidal Glutamatergic Neurons

The astrocyte cluster was extracted from the dataset using Seurat's subset function. Variable features were selected using the "mvp" method and default parameters. Clustering and UMAP projection were performed as above, using the first 50 PCs as input. The same procedure was applied to the EN-P population but using the first 70 PCs for clustering and UMAP projection.

GO Enrichment Analyses

Metascape (Zhou et al. 2019), available at https://metascape.org was used for all gene ontology enrichment analyses, with the following custom settings: A background gene list of the 22,097 genes in our dataset was provided, and gene enrichment was performed on the GO Biological Processes database. Metascape performs hierarchical clustering of the enriched terms, and the most significant term within a cluster was selected as the representative term to report in GO enrichment results. The representative GO terms were filtered using a Q-value cutoff of 0.05.

Differential Gene Expression Analyses

Before testing for differentially expressed genes between control and *MAPT* mutant cells, the number of control and mutant cells were balanced for each isogenic pair, i.e. the sample with the higher number of cells was subsetted to the number of cells in the sample with the lower number of cells. This was done to prevent differences between cell lines or batches from confounding the analyses. For comparisons to the V337M mutant cell line GIH7-C2Δ2A01, the isogenic control GIH7-C2Δ2F02 was used and GIH7-C2Δ2B12 was disregarded due to an almost complete lack of pyramidal neurons (EN-P) and abundance of unknown neuron (IN and N) categories (Supplementary Figure 1B). All differential gene expression analyses of *MAPT* mutant vs. control cells were carried out using the MAST test (Finak et al. 2015) as implemented in the Seurat package with a logFC cutoff of 0 and a minimum percentage of 0 (logFC.cutoff = 0, min.pct = 0). Benjamini-Hochberg corrected p-values < 0.05 were considered statistically significant. A logFC cutoff of 0.05 was applied after differential gene expression testing to identify genes of interest. Packages used to visualize results were pheatmap (Kolde 2019) and EnhancedVolcano (Blighe, Rana, and Lewis 2019).

Gene Signature Scoring

Gene signature scoring was performed using Seurat's AddModuleScore function with default parameters and a bin size of 8. Scoring was performed on the gene signature "superpathway of cholesterol biosynthesis", deposited in the Human Cyc database and comprising 25 genes (ACAT1, ACAT2, CYP51A1,

DHCR24, DHCR7, EBP, FDFT1, FDPS, GGPS1, HMGCR", HMGCS1, HMGCS2, HSD17B7, IDI1, IDI2, LBR, LSS, MSMO1, MVD, MVK, NSDHL, PMVK, SC5D, SQLE, TMSF2).

Immunohistochemistry of Organoid Sections

Immunohistochemistry was performed on isogenic lines from 3 different mutation carriers (R406W1, V337M2, V337M3), and in one or two differentiation batches per line (R406W1: 2 batches, V337M2: 2 batches, V337M3: 1 batch using ctrl2 as isogenic control, see "Cell Lines and Maintenance of Human Induced Pluripotent Stem Cells (iPSCs)" subsection for full line names. 3-4 organoids were evaluated for co-expression of HMGCS1 and GFAP for each cell line and condition.

Organoids were fixed overnight in 4% paraformaldehyde (Santa Cruz), sunk in 30% sucrose, embedded in OCT compound and rapidly frozen before tissue was cryosectioned at 20 µm thickness, with a Leica cryostat model CM3050. All solutions were used at approximately 500uL/slide. Slides were thawed to room temperature (RT), blocked in 10% Normal Goat Serum (NGS), 3% Bovine Serum Albumin (BSA), 0.3% Triton-X 100 in phosphate buffered saline (PBS) for 1 hour at RT. Slides were incubated with anti-HMGCS1 (Abcam ab155787) 1:100 in block solution overnight at 4°C. Sections were then incubated with goat anti-rabbit IgG-488 (Invitrogen) antibody diluted 1:1000 in block solution for 1 hour at RT. Slides were co-labeled with GFAP (Millipore MAB-3402) 1:400 in block overnight at 4°C. GFAP was revealed by incubating with secondary antibody Goat anti mouse IgG_1-546 (Invitrogen) at 1:1000 in block for 1 hour at RT. Slides were incubated with DAPI (Sigma D-1306) at 1:1000 in PBS 10 min at RT to label nuclei. High power images were taken with a Zeiss Axio-Observer Z1 epifluorescence microscope. GFAP+ cells that were co-stained for HMGCS1 were quantified. Images of 4 sections per organoid were taken; Sections were randomly chosen to be representative of the whole organoid - top to bottom and left to right.

Lipidomics

For free sterol/oxysterol analysis, individual snap-frozen cerebral organoids were homogenized into 200uL of 10% methanol in water. An internal standard mix of 25-Hydroxycholesterol-d6, Desmosterol-d6, and Campesterol-d6 (Avanti Polar Lipids) was added to 180uL of homogenate. Samples were extraction using a modified BUME extraction (Löfgren et al., 2016). Extracts were brought to dryness and taken up in 90% methanol in water and run on a Waters Acquity UPLC interfaced with an AB Sciex 6500 QTrap mass spectrometer equipped with an APCI probe. Source settings were: Curtain Gas=20, Collision Gas=Medium,

Ion Spray Voltage=5500, Temperature=400, GS1=25, GS2=15. A Phenomenex Kinetex C18 1.7uM 2.1mm x 150mm column was used for chromatographic separation. A 30min step gradient was employed using 70/30 Acetonitrile/Water with 5mM Ammonium Acetate as Buffer A and 50/50 acetonitrile/water with 5mM Ammonium Acetate as Buffer B with a flow of 0.5 mL/min. The gradient started at 0%B for 2 minutes, ramped to 10%B over 4 minutes, 15%B over 9 minutes, 50%B over 11 minutes, 100%B over 2 minutes, then held at 100%B for 2 minutes. Sterol species were identified by mass spectrometry on a 6500 Qtrap instrument (Sciex) using 30 MRMs (Multiple Reaction Monitoring) in positive mode. Standard curves were obtained in parallel using identical conditions. Data analysis was performed with Analyst and Mulitquant software packages.

Supplemental References

Butler, A., Hoffman, P., Smibert, P., Papalexi, E., and Satija, R. (2018). Integrating single-cell transcriptomic data across different conditions, technologies, and species. *Nat Biotechnol*, *36*(5), 411–420. 10.1038/nbt.4096

Finak, G., McDavid, A., Yajima, M., Deng, J., Gersuk, V., Shalek, A. K., Slichter, C. K., Miller, H. W., McElrath, M. J., Prlic, M., et al. (2015). MAST: a flexible statistical framework for assessing transcriptional changes and characterizing heterogeneity in single-cell RNA sequencing data. *Genome Biol*, *16*, 278. 10.1186/s13059-015-0844-5

Giandomenico, S. L., Mierau, S. B., Gibbons, G. M., Wenger, L., Masullo, L., Sit, T., Sutcliffe, M., Boulanger, J., Tripodi, M., Derivery, E., et al. (2019). Cerebral organoids at the air-liquid interface generate diverse nerve tracts with functional output. *Nat Neurosci*, *22*(4), 669–679. 10.1038/s41593-019-0350-2

Gregory, J. A., Hoelzli, E., Abdelaal, R., Braine, C., Cuevas, M., Halpern, M., Barretto, N., Schrode, N., Akbalik, G., Kang, K., et al. (2020). Cell Type-Specific In Vitro Gene Expression Profiling of Stem Cell-Derived Neural Models. *Cells*, *9*(6), 1406. 10.3390/cells9061406

Huang, M., Wang, J., Torre, E., Dueck, H., Shaffer, S., Bonasio, R., Murray, J. I., Raj, A., Li, M., and Zhang, N. R. (2018). SAVER: gene expression recovery for single-cell RNA sequencing. *Nat Methods*, *15*(7), 539–542. 10.1038/s41592-018-0033-z

Karch, C. M., Kao, A. W., Karydas, A., Onanuga, K., Martinez, R., Argouarch, A., Wang, C., Huang, C., Sohn, P. D., Bowles, K. R., et al. (2019). A Comprehensive Resource for Induced Pluripotent Stem Cells from Patients with Primary Tauopathies. *Stem Cell Reports*, *13*(5), 10.1016/j.stemcr.2019.09.006

Löfgren, L., Forsberg, G. B., and Ståhlman, M. (2016). The BUME method: a new rapid and simple chloroform-free method for total lipid extraction of animal tissue. *Sci Rep*, *6*, 27688. 10.1038/srep27688

Macosko, E. Z., Basu, A., Satija, R., Nemesh, J., Shekhar, K., Goldman, M., Tirosh, I., Bialas, A. R., Kamitaki, N., Martersteck, E. M., et al. (2015). Highly Parallel Genome-wide Expression Profiling of Individual Cells Using Nanoliter Droplets. *Cell*, *161*(5), 1202–1214. 10.1016/j.cell.2015.05.002

McGinnis, C. S., Murrow, L. M., and Gartner, Z. J. (2019). DoubletFinder: Doublet Detection in Single-Cell RNA Sequencing Data Using Artificial Nearest Neighbors. *Cell Syst*, *8*(4), 329–337.e4. 10.1016/j.cels.2019.03.003

Polioudakis, D., de la Torre-Ubieta, L., Langerman, J., Elkins, A. G., Shi, X., Stein, J. L., Vuong, C. K., Nichterwitz, S., Gevorgian, M., Opland, C. K., et al. (2019). A Single-Cell Transcriptomic Atlas of Human Neocortical Development during Mid-gestation. *Neuron*, *103*(5), 785–801.e8. 10.1016/j.neuron.2019.06.011

Quadrato, G., Nguyen, T., Macosko, E. Z., Sherwood, J. L., Min Yang, S., Berger, D. R., Maria, N., Scholvin, J., Goldman, M., Kinney, J. P., et al. (2017). Cell diversity and network dynamics in photosensitive human brain organoids. *Nature*, *545*(7652), 48–53. 10.1038/nature22047

Stuart, T., Butler, A., Hoffman, P., Hafemeister, C., Papalexi, E., Mauck, W. M., 3rd, Hao, Y., Stoeckius, M., Smibert, P., and Satija, R. (2019). Comprehensive Integration of Single-Cell Data. *Cell*, *177*(7), 1888–1902.e21. 10.1016/j.cell.2019.05.031

Velmeshev, D., Schirmer, L., Jung, D., Haeussler, M., Perez, Y., Mayer, S., Bhaduri, A., Goyal, N., Rowitch, D. H., and Kriegstein, A. R. (2019). Single-cell genomics identifies cell type-specific molecular changes in autism. *Science*, *364*(6441), 685–689. 10.1126/science.aav8130

Yoon, S. J., Elahi, L. S., Pașca, A. M., Marton, R. M., Gordon, A., Revah, O., Miura, Y., Walczak, E. M., Holdgate, G. M., Fan, H. C., et al. (2019). Reliability of human cortical organoid generation. *Nat Methods*, *16*(1), 75–78. 10.1038/s41592-018-0255-0

Zhou, Y., Zhou, B., Pache, L., Chang, M., Khodabakhshi, A. H., Tanaseichuk, O., Benner, C., and Chanda, S. K. (2019). Metascape provides a biologist-oriented resource for the analysis of systems-level datasets. *Nat Commun*, *10*(1), 1523. 10.1038/s41467-019-09234-6
